# Genome-wide identification and characterization analysis of *RWP-RK* family genes reveal their role in flowering time of *Chrysanthemum lavandulifolium*

**DOI:** 10.1186/s12870-023-04201-2

**Published:** 2023-04-15

**Authors:** Qiuling Zhang, Junzhuo Li, Xiaohui Wen, Chengyan Deng, Xiuzhen Yang, Silan Dai

**Affiliations:** 1 Beijing Key Laboratory of Ornamental Plants Germplasm Innovation & Molecular Breeding, National Engineering Research Center for Floriculture, Beijing Laboratory of Urban and Rural Ecological Environment, Key Laboratory of Genetics and Breeding in Forest Trees and Ornamental Plants of Education Ministry, School of Landscape Architecture, Beijing Forestry University, Beijing, 100083 China; 2 Zhejiang University, No. 866 Yuhangtang Road, Hangzhou, 310058 China; 3 Linyi University, Shandong, 276000 China; 4 School of Landscape Architecture, Beijing Forestry University, Beijing, 100083 China

**Keywords:** Nitrogen, Nitrate, RWP-RK, Photoperiod pathway, Flowering time

## Abstract

**Background:**

RWP-RKs are plant specific transcription factors, which are widely distributed in plants in the form of polygenic families and play key role in nitrogen absorption and utilization, and are crucial to plant growth and development. However, the genome-wide identification and function of RWP-RK in Compositae plants are widely unknown.

**Results:**

In this study, 101 RWP-RKs in *Chrysanthemum lavandulifolium* were identified and tandem repeat was an important way for the expansion of RWP-RKs in Compositae species. 101 RWP-RKs contain 38 NIN-like proteins (NLPs) and 31 RWP- RK domain proteins (RKDs), as well as 32 specific expansion members. qRT-PCR results showed that 7 *ClNLPs* in leaves were up-regulated at the floral transition stage, 10 *ClNLPs* were negatively regulated by low nitrate conditions, and 3 of them were up-regulated by optimal nitrate conditions. In addition, the flowering time of *Chrysanthemum lavandulifolium* was advanced under optimal nitrate conditions, the expression level of *Cryptochromes* (*ClCRYs*), *phytochrome C* (*ClPHYC*) and the floral integration genes *GIGANTEA* (*ClGI*), *CONSTANS-LIKE* (*ClCOL1*, *ClCOL4*, *ClCOL5*), *FLOWERING LOCUS T* (*ClFT*), *FLOWERING LOCUS C* (*ClFLC*), *SUPPRESSOR OF OVER-EXPRESSION OF CONSTANS 1* (*ClSOC1*) also were up-regulated. The expression level of *ClCRY1a*, *ClCRY1c*, *ClCRY2a* and *ClCRY2c* in the vegetative growth stage induced by optimal nitrate reached the expression level induced by short-day in the reproductive growth stage, which supplemented the induction effect of short-day on the transcription level of floral-related genes in advance.

**Conclusions:**

It was speculated that *ClNLPs* may act on the photoperiodic pathway under optimal nitrate environment, and ultimately regulate the flowering time by up-regulating the transcription level of *ClCRYs*. These results provide new perspective for exploring the mechanism of nitrate/nitrogen affecting flowering in higher plants.

**Supplementary Information:**

The online version contains supplementary material available at 10.1186/s12870-023-04201-2.

## Background

As an important part of life components such as nucleic acids, amino acids, and proteins, nitrogen (N) plays an important role in the growth and development of plants and is one of the indispensable nutrient elements for plants [22]. As nutrient and signaling molecule in plants, plants need to constantly sense available N in the environment and respond accordingly [3, 36]. However, the N fertilizer utilization efficiency of plants is not high and most of the N fertilizer is lost to the environment, which eventually leads to soil, water and air pollution [23]. Therefore, more and more attention has been paid to improving nitrogen use efficiency (NUE), reducing N fertilizer application, and cultivating high-quality ornamental plants with high N use efficiency.

The utilization of N by plants is often inseparable from the participation of various transporters. The transportation of N absorbed by roots is mainly completed by nitrate transporters, such as the first nitrate transporter nitrate transporter1.1 (NRT1.1) discovered in *Arabidopsis thaliana*, protein kinase 8 (CIPK8) and CIPK23 can regulate high-affinity or low-affinity nitrate reaction by phosphorylating NRT1.1 [34]. Plant assimilation of N also involves the participation of a series of proteins, such as ammonium transporters (AMTs) and nitrate reductases (NRs) [2]. Transcription factors (TFs) involved in N signaling pathway also play a key role in N absorption and assimilation [10]. RWP-RKs are a class of TFs that can sense nitrate signals and control N absorption and utilization, which contains two subfamilies: RKDs (RWP-RK domain proteins) and NLPs (NIN-like proteins) [27]. RKDs only have a conserved domain: RWP-RK, which can bind to DNA. In addition to the RWP-RK domain, NLPs also have a functional PB1 domain with protein-protein interaction at the carboxyl end, and a GAF-like domain at the amino end. Currently, 14 *RWP-RK* homologous genes(5 *RKDs* and 9 *NLPs*)have been identified in *A. thaliana* [25]. Studies have shown that *NLPs* mainly regulate the tissue-specific expression of NUE-related genes [5, 13, 14, 44]. Transcriptional activation by *NLPs* to regulate N assimilation and metabolism or inhibit downstream genes by binding to key N pathway genes [21], *RKDs* mainly regulate the expression of genes related to gametogenesis/embryogenesis [5, 17].

With the rapid development of RNA-seq technology, genome-wide identification and functional studies of *RWP-RKs* or *NLPs* in *A. thaliana* [13, 40], rice (*Oryza sativa*) [11], wheat (*Triticum aestivum*) [16], maize (*Zea mays*) [8], *Brassica napus* [19] and other species have been performed. Among them, functional studies have shown that *AtNLPs* can bind to Nitrate-responsive elements (NREs) in target gene promoters, thereby playing a key role in coordinating primary N responses [4, 12, 13]. Among non-nodulating plants, the most studied member is NODULEINCEP TION like protein 7 (NLP7), which can respond to nitrate signaling, regulate gene expression, and participate in N metabolism [4, 24]. Overexpression of *AtNLP7* can promote plant growth by enhancing N and carbon assimilation [44]. In addition, *AtNLP8* promotes seed germination by regulating nitrate and directly binds to the promoter of abscisic acid (ABA) catabolic enzyme gene to reduce ABA level in a nitrate-dependent manner [41]. Under N starvation and continuous nitrate treatment, *AtNLP6* and *AtNLP7* can interact with TEOSINTE BRANCHED1/CYCLOIDEA/PROLIFERATING CELL FACTOR1-20 (TCP1-20) [9]; overexpression of rice *OsNLP1* gene can promote rice growth, as well as improve N use efficiency and yield [1].

As traditional famous flower in China, chrysanthemum (*Chrysanthemum* ×*morifolium*) has a very high ornamental value and a wide range of applications. Previous studies have found that in addition to responding to photoperiod regulation [42, 43], the application of optimal N fertilizer under the same photoperiod conditions can promote the flowering period of chrysanthemums, and both high N or N starvation conditions delay flowering, indicating that N has plays an important role in flowering of chrysanthemum [30, 32, 46, 47] The *RWP-RKs* gene plays a key role in N absorption and utilization, and are extremely important for plant growth and development [5, 7, 27]. However, there is a lack of research on *RWP-RKs* gene in chrysanthemum, especially the relationship between *RWP-RKs* gene and flowering has not been reported. In this study, based on the genome database of *C. lavandulifolium*, a diploid species of the genus Compositae, 101 *RWP-RKs* genes were first identified, then conserved motifs, gene structure and promoter elements were preliminarily analyzed. At the same time, the expression patterns of key genes were analyzed based on 45 *RWP-RKs* genes identified in the *C. lavandulifolium* transcriptome database. The flowering of *C. lavandulifolium* under different nitrate conditions was further compared, and qRT-PCR was used to verify the expression of key floral-related genes in the photoperiod pathway. The potential regulation mechanism between RWP-RKs and flowering time was constructed finally. This study not only provided an in-depth understanding of the *RWP-RK* gene in chrysanthemum, but also laid an important theoretical foundation for the subsequent analysis of the relationship between N signaling pathway and floral regulation pathway.

## Results

### Identification and phylogenetic tree analysis of RWP-RKs

**Fig. 1:**
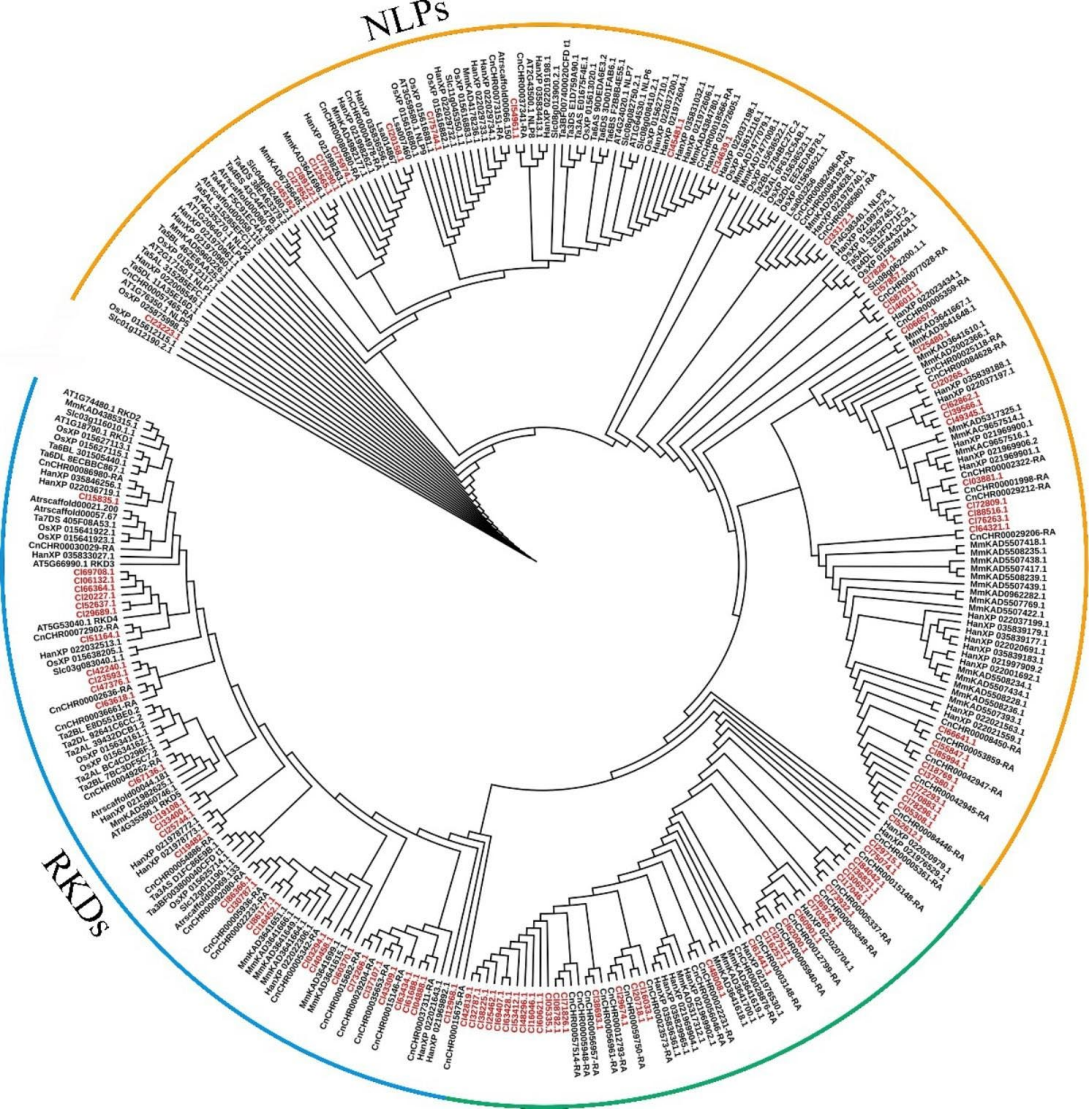
Phylogenetic analysis of the RWP-RK from *C. lavandulifolium*, *A. thaliana* and other representative species used TBtools with the ML method. Members marked in red represent *C. lavandulifolium* ClRWP-RK. Orange line part represents NLPs subfamily, blue line part represents RKDs subfamily, and green line part represents specific distribution branch

Based on the genome of *C. lavandulifolium*, some genomes of other Compositae plants, and online databases search for RWP-RK members. According to the results of multiple sequence alignment and the conserved domain results of the NCBI website, a total of 101 RWP-RK were finally identified in the *C. lavandulifolium* genome and different numbers of RWP-RKs were identified in other Compositae plants. The distribution of RWP-RKs in *C. lavandulifolium* is the most, followed by 56, 52, and 41 in *Chrysanthemum nankingense*, *Helianthus annuus* and *Mikania micrantha*, indicating that members of RWP-RKs expanded in Compositae plants. The distribution of RWP-RKs in *Lactuca sativa* was relatively small, only 27. Unlike the distribution of members in Compositae species was quite different, generally fewer RWP-RKs were identified in representative species such as *Arabidopsis thaliana, Amborella trichopoda*, *Oryza sativa*, *Solanum lycopersicum*, and *Triticum aestivum*, 14, 6, 22, 10, 26 RWP-RKs members were identified respectively (Fig. 1, Table. S1). The widespread distribution of RWP-RKs may involve the large-scale worldwide coverage of Compositae species in a short time.

In order to understand the early history and evolutionary relationship of RWP-RK in plant lineages, as well as the changes in shrinkage/expansion of *RWP-RK* gene family in Compositae plants, an ML tree was constructed for phylogenetic analysis by using RWP-RKs obtained from *C. lavandulifolium*, 4 Compositae species and 5 other representative species (Fig. 1). According to the evolutionary relationship of *RWP-RK* gene family members in representative species, *RWP-RKs* genes were divided into two representative subfamilies: RKDs and NLPs. In addition, ClRKDs and ClNLPs in Compositae plants had member specific expansion events. Through the phylogenetic tree, we found the specificity of the distribution of some members (green line part). This branch does not contain RWP-RK of the representative species, and the conserved domain of the distributed genes contain two types, indicating that RWP-RK in Compositae species have been specifically divided (Table. S1). Among them, there are 38 ClNLPs and 31 ClRKDs, as well as 32 specific expansion members in *C. lavandulifolium*.

### Chromosomal localization analysis of RWP-RKs

**Fig. 2:**
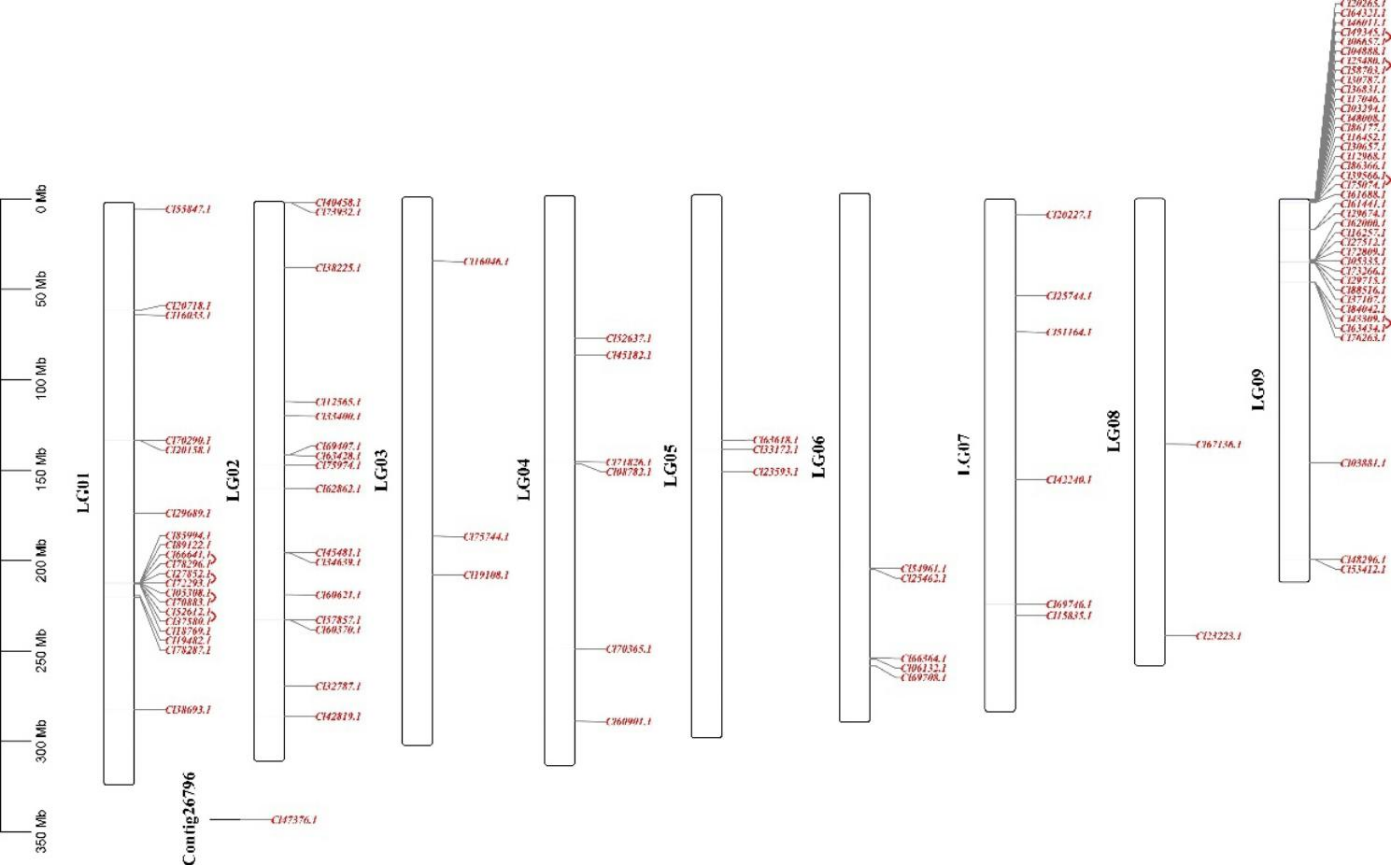
Distribution of 101 *ClRWP-RK* genes on 9 chromosomes. Chromosomes are represented by black hollow vertical bars. *ClRWP-RK* genes are written in red colour

The chromosomal localization information related to RWP-RKs was retrieved from the *C. lavandulifolium* genome database, and the chromosomal localization distribution map was made by using TBtools. As shown in Figs. 2 and 100 of the 101 *ClRWP-RKs* genes were mapped to 9 chromosomes of *C. lavandulifolium*, and one *ClRWP-RK* gene (Cl47376.1) was not assembled on the chromosome. These genes were unevenly distributed on chromosomes. The number of genes on chromosome 9 was the largest, reaching 39, followed by 20 and 16 genes on chromosomes 1 and 2, respectively. The other chromosomes have fewer members, with the number on chromosomes 8 only 2 genes, and only 3 genes on chromosomes 3 and 5. In addition to chromosomes 3 and chromosomes 8, tandem duplications occurred on other chromosomes, especially on chromosomes 1 and 9, which formed distinct gene clusters, suggesting that tandem duplication events were an important way for the expansion of *RWP-RKs* in *C. lavandulifolium*.

### Gene structures, conserved motifs, conserved domains and promoter cis-elements analysis of RWP-RKs

**Fig. 3:**
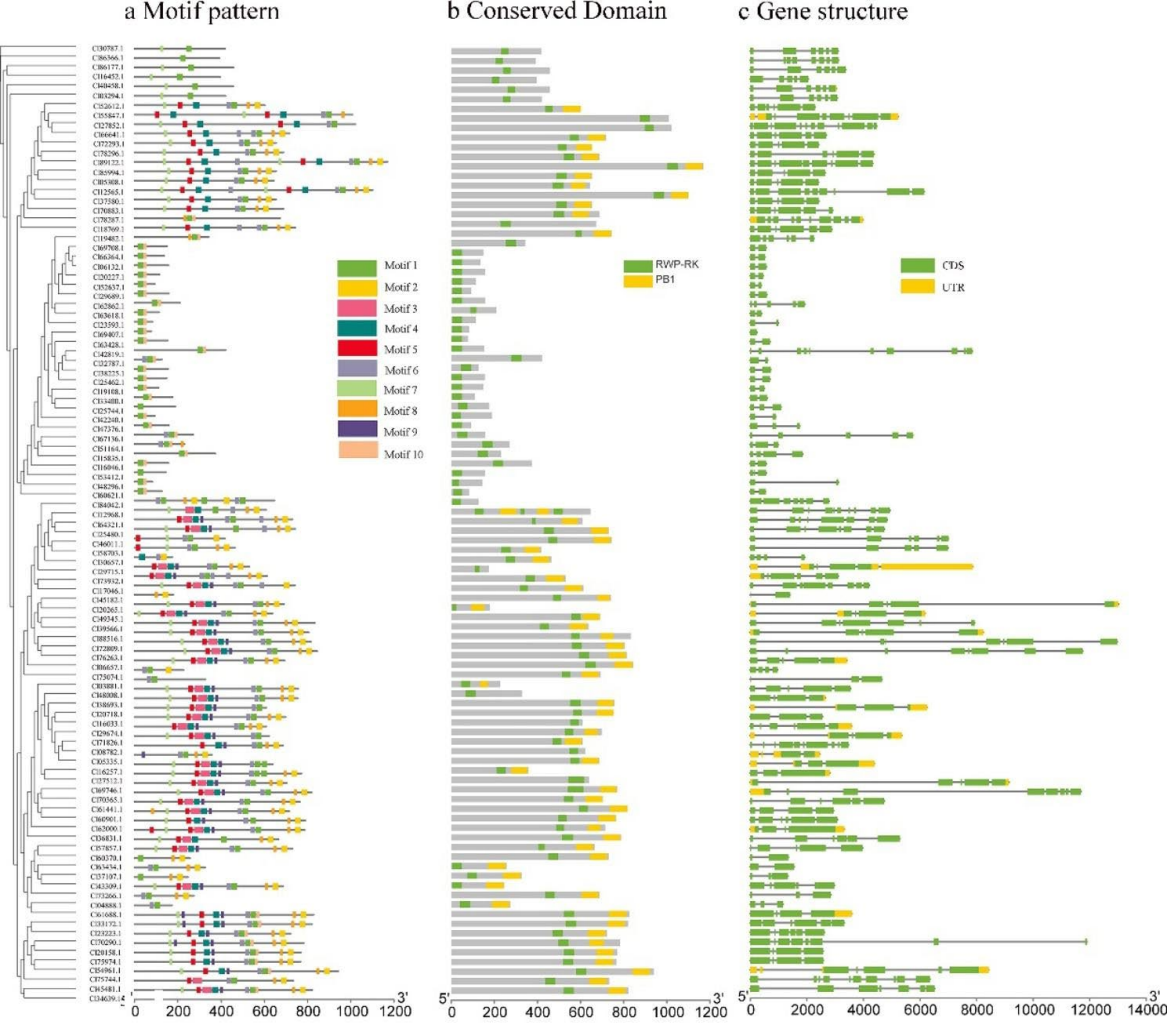
Motif composition, conserved domains and gene structures analysis of ClRWP-RKs. **a** Display different motif components of different protein sequences according to different colors. **b** Conserved domain of ClRWP-RK showing distribution of RWP-RK domain (solid green bars) and PBI domain (solid orange bars). **c** Structure of *ClRWP-RK* genes showing distribution of CDS (solid green bars), introns (black lines), UTR regions (solid orange bars)

Using MEME online tool to predict the conserved motifs of ClRWP-RK, 10 conserved motifs were finally identified and labeled as Motif 1–10 (Fig. 3a). ClRWP-RK proteins had 2–10 conserved motifs, and all ClRWP-RK proteins contain Motif 1.

The conserved domains of ClRWP-RK protein were verified by the CDD database of NCBI. The results showed that members of the ClRKDs subfamily contained one RWP-RK domain, while the ClNLPs subfamily contained one RWP-RK and one PBI domain, indicating that RWP-RK family of *C. lavandulifolium* were more conservative (Fig. 3b). Overall, the PBI domains of ClNLPs subfamily were mostly at the N-terminus. Compared with the *A. thaliana* (Fig. S1), the ClRWP-RK protein domain was evolutionarily conserved.

According to the corresponding ClRWP-RK protein coding sequence and genome sequence, the *ClRWP-RKs* gene structure map was constructed (Fig. 3c). The number of introns and exons of *ClRWP-RK* varied greatly, with the number of introns at least 1 and at most 11, as well as the number of exons at least 1 and at most 12. There were certain differences in the size and position of introns and exons in different genes.

The cis-acting elements on the *ClRWP-RK* promoter were predicted and visualized (Fig. S2a). In addition to containing the TATA-box and CAAT-box elements necessary for growth, there were a variety of response elements in the promoter region, including light response elements, hormone response elements (MeJA, ABA, IAA, GA, salicylic acid), abiotic stress response elements (dehydration, low temperature, salt stress). In addition, a N-responsive element GCN4 was also predicted in the promoter regions of 17 genes, which was speculated to play a role in N stress response. A circadian regulatory element (CAAAGATATC) was predicted in the promoter regions of 31 genes. When counting the number of promoter elements, it was found that the most predictive cis-acting element were light response elements, followed by Methyl jasmonate (MeJA) and abscisic acid-related (ABA) elements (Fig. S2b). This provides important clues to subsequent in-depth analyze the function of *ClRWP-RK* genes.

### Expression pattern analysis of ***RWP-RK*** in leaves and apical meristems of ***C. lavandulifolium***

**Fig. 4:**
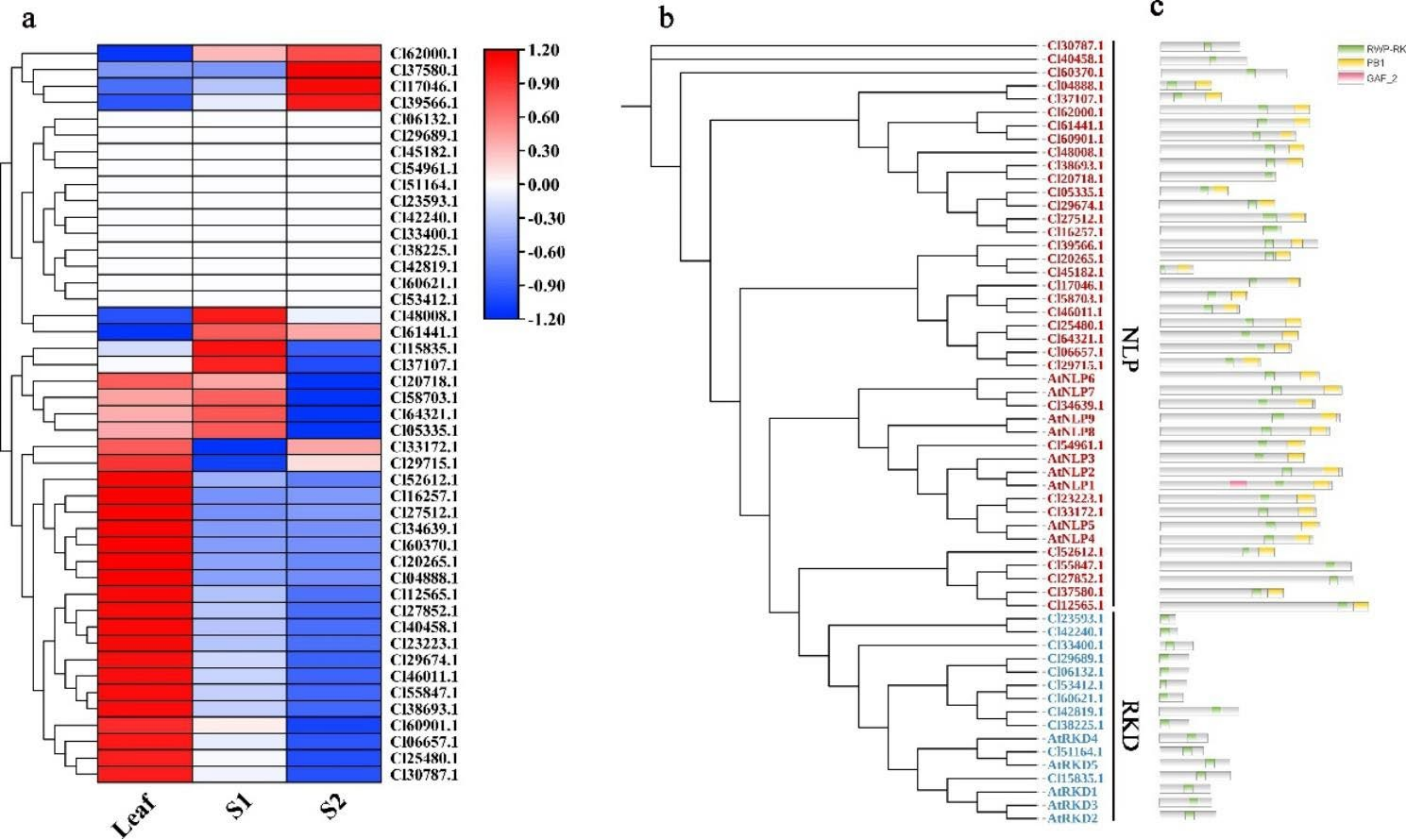
Expression of *ClRWP-RKs* in leaves and apical meristems before and under floral transition. **a** Construction of gene expression heat map based on FPKM values in the *C. lavandulifolium* transcriptome dataset. Leaf represents leaves of *C. lavandulifolium*, S1 and S2 represent the apical meristem before and under floral transition in *C. lavandulifolium*, respectively. **b** Phylogenetic tree of *ClRWP-RKs* identified in the *C. lavandulifolium* transcriptome and *AtRWP-RKs* genes. **c** Conserved domains of ClRWP-RKs and AtRWP-RKs

Based on the *C. lavandulifolium* transcriptome dataset [37, 38], the expression pattern of *RWP-RKs* was analyzed. A total of 45 *ClRWP-RK* genes were identified in the leaves and apical meristems of before and under floral transition, of which 33 *ClRWP-RK* genes were differentially expressed in different tissues and 12 were not expressed (Fig. 4a, Table S3). These 45 genes were classified by combining evolutionary relationships and distributions of conserved domain (Fig. 4b and c). Among them, only one gene (Cl15835.1, ClRKD1) was identified in the *ClRKDs* branch, which was expressed in leaves (Leaf) and apical meristem before floral transition stage (S1), with the highest expression in S1 stage, while other members of *ClRKDs* were not expressed (Fig. 4a-c), indicating that *ClNLPs* gene may play a major role in the apical development. According to the expression pattern of *ClRWP-RK* gene, it could be divided into the following five patterns: high expression in Leaf only, low expression in apical meristem (S1, S2); high expression in L and S1, low expression in the apical meristem under floral transition stage (S2); high expression in S1 and S2, low expression in Leaf; high expression in S2 only; no expression in three tissues. The different expression patterns of these genes suggested that they may play different functions during the development of apical meristem, thus ultimately affecting the flowering process of *C. lavandulifolium*.

### Expression pattern analysis of ***ClNLPs***

#### Tissue specific expression pattern of ***ClNLPs***

**Fig. 5:**
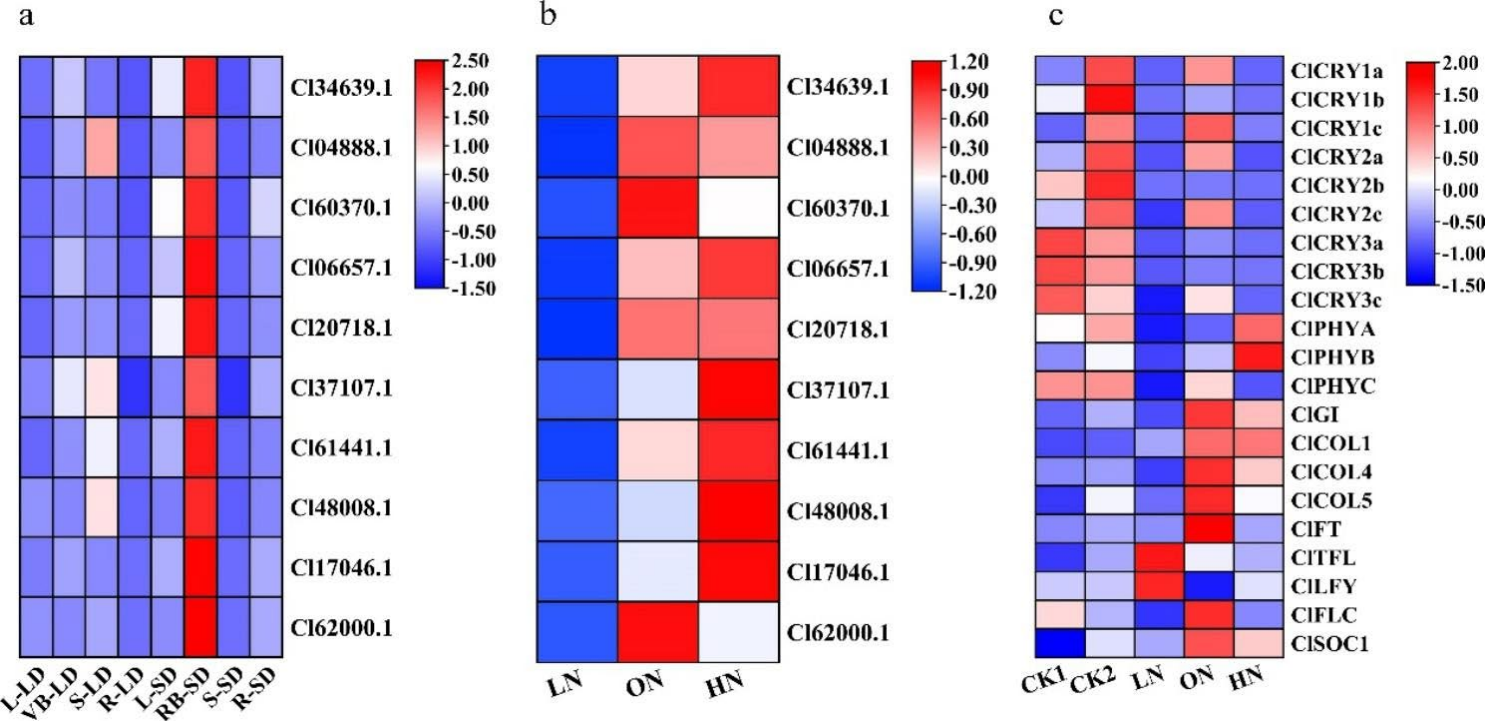
Expression pattern analysis of *ClNLPs* genes. **a** Tissue-specific expression pattern of *ClNLPs* in L-LD (leaf at long-day stage), VB-LD (apical meristem at long-day stage), S-LD (stem at long-day stage), R-LD (root at long-day stage), L-SD (leaf at short -day stage), VB-SD (apical meristem at short-day stage), S-SD (stem at short -day stage), and R-SD (root at short -day stage). **b** Expression patterns of *ClNLPs* genes under different nitrate conditions. Low nitrate, LN, 0.1 mM KNO_3_; optimal nitrate, ON, 1.5 mM KNO_3_; high nitrate, HN, 3.0 mM KNO_3_. **c** Expression patterns of floral-related genes of *C. lavandulifolium* photoperiod pathway under different nitrate conditions. CK1 and CK2 represent *C. lavandulifolium* leaves at long-day and short-day stages, respectively, under normal water and fertilizer management without metering; low nitrate, LN, 0.1 mM KNO_3_; optimal nitrate, ON, 1.5 mM KNO_3_; high nitrate, HN, 3.0 mM KNO_3_

Based on the expression analysis of *RWP-RKs* genes in the transcriptome of *C. lavandulifolium*, except for the genes that were not expressed in the three samples, two representative genes with high expression were screened in the other four expression patterns. Because the most genes were expressed in leaves, four representative genes were selected. The phylogenetic tree showed that these 10 *ClRWP-RK* genes were clustered into *ClNLP* subfamily members (Fig. 4b), most of which were unique to *C. lavandulifolium*. The tissue specificity of the expression levels of 10 *ClNLP* genes before and under floral transition stages were validated (Fig. 5a, S3). qRT-PCR results showed that the expression levels of all 10 *ClNLP* genes were the highest in apical meristem (RB) at the floral transition stage. Before floral transition (vegetative growth stage), only the Cl04888.1 and Cl37107.1 were highly expressed in stem tissue (S), followed by apical meristem (VB), and all genes were expressed lowest in root (R). Entering the floral induction stage, the expression levels of 10 *ClNLP* genes in RB and R were induced to increase, and the expression levels in S were down regulated, among which expression levels of 7 genes (Cl34639.1, Cl04888.1, Cl60370.1, Cl06657.1, Cl20718.1, Cl61441.1, Cl17046.1) were induced to increase in L. It was speculated that these *ClNLP* genes may play an important role in the floral transition stage of *C. lavandulifolium*.

#### Expression patterns of ***ClNLPs*** under different nitrate concentrations

The expression of 10 *ClNLP* genes under different nitrate concentrations was verified (Fig. 5b, S4). qRT-PCR results showed that the expression levels of all *ClNLP* genes under low nitrate (LN) conditions were lower than those under optimal nitrate (ON) and high nitrate conditions (HN), indicating that their expressions were all negatively regulated by LN conditions. Under ON conditions, Cl04888.1, Cl60370.1 and Cl62000.1 showed high expression, indicating that they were induced by ON conditions. Cl34639.1 (*ClNLP7*), Cl06657.1, Cl37107.1, Cl61441 0.1, Cl48008.1, Cl17046.1 showed high expression under HN conditions, indicating that their expression was induced by HN conditions. Cl20718.1 was induced to express at ON and HN conditions, and only showed inhibition of expression at LN conditions. *ClNLP* genes showed differential expression pattern under different nitrate conditions, indicating that different *ClNLP* genes may play different regulatory roles in the development of *C. lavandulifolium*.

### Flowering time and expression patterns of floral-related genes under different nitrate concentrations of ***C. lavandulifolium***

**Fig. 6:**
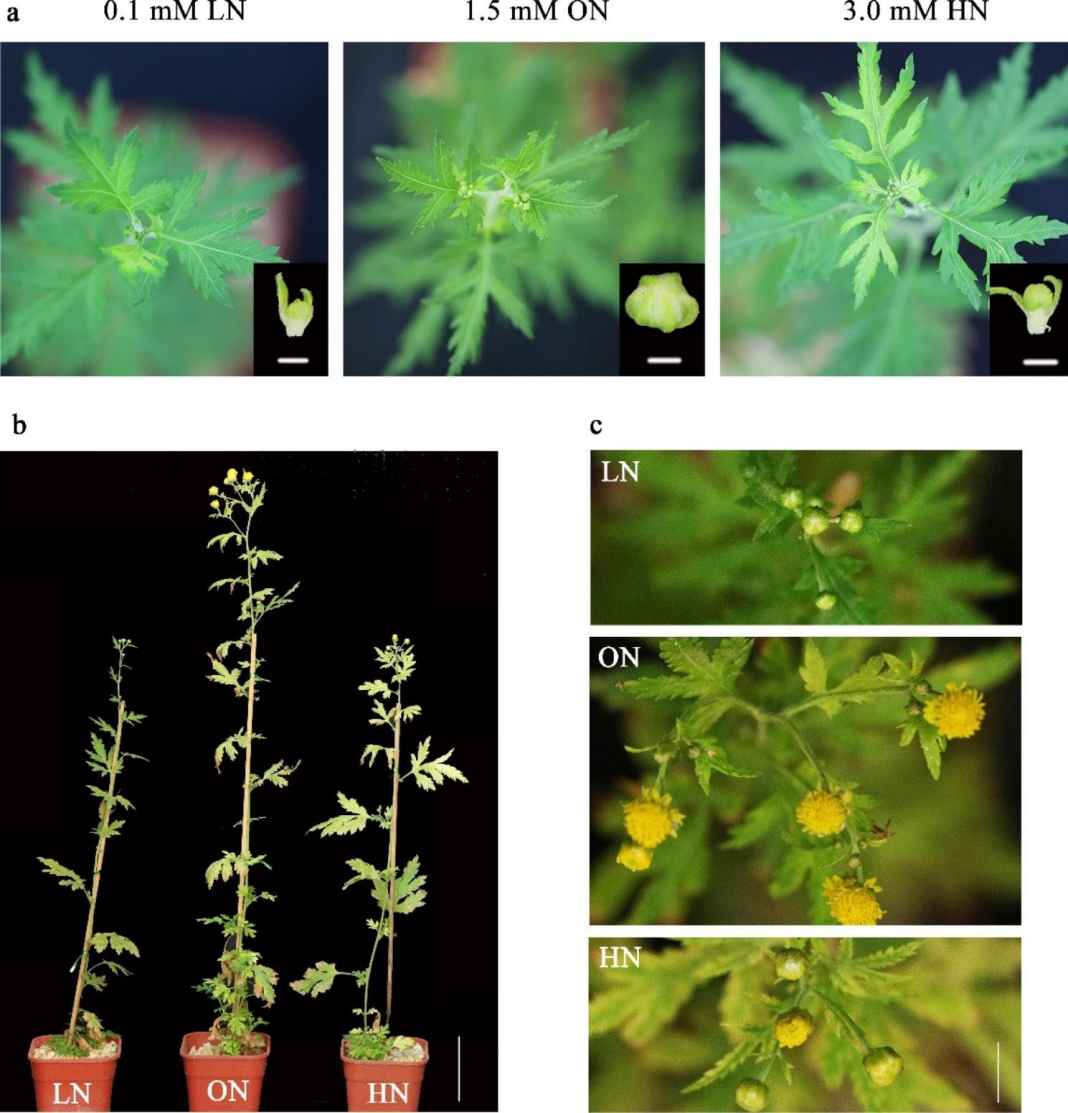
Flowering time of *C. lavandulifolium* under different nitrate concentration. **a** Phenotype of *C. lavandulifolium* at budding stage (Bar = 1 mm). **b** Phenotype of *C. lavandulifolium* at flowering stage (Bar = 5 cm). **c** Apical inflorescence phenotype of *C. lavandulifolium* at flowering stage (Bar = 1 cm). low nitrate, LN, 0.1 mM KNO_3_; optimal nitrate, ON, 1.5 mM KNO_3_; high nitrate, HN, 3.0 mM KNO_3_

As a typical short-day plant, *C. lavandulifolium* needs to complete vegetative growth under long-day conditions, complete the floral transition through short-day induction, and finally reach flowering state [48]. Overexpression of the photoreceptor genes *ClCRY1s* and *ClCRY2* can significantly advance flowering time, and the *ClCRYs* gene play key role in floral transition of *C. lavandulifolium* [42, 43]. In the observation of the flowering time under different nitrate concentrations (Fig. 6, S7), it was found that flowering time was advanced under ON conditions, but delayed under LN and HN conditions. It indicated that nitrate played an important role in the flowering time of *C. lavandulifolium*.

Taking the leaves at the vegetative growth stage (long-day, CK1) and reproductive growth stage (short-day, CK2) under normal water and fertilizer management cultivation conditions (no statistics on nitrate/nitrogen application) as the CK, combined with leaves under different nitrate concentration treatments (vegetative growth stage), qRT-PCR was used for comparative analysis. The results showed that the photoreceptor genes *ClCRY1s*, *ClCRY2s*, *ClPHYs* and the floral integration genes *ClGI*, *ClCOLs*, *ClFT* and *ClSOC1* in the photoperiod pathway were all up-regulated by short-day induction at the short-day induction stage (CK2) (Fig. 5c, S5, S6). In addition, *ClCRYs*, *ClPHYC*, *ClGI*, *ClCOL4*, *ClCOL5*, *ClFT*, *ClFLC* and *ClSOC1* were all up-regulated under ON conditions and suppressed under LN conditions. Among them, *ClPHYA* and *ClPHYB* were highly expressed under HN conditions, while the flower formation inhibition gene *ClTFL* was down-regulated under ON conditions (Fig. 5c, S6, S6). Combined with the short-day flowering characteristics [42, 43, 48] and the difference analysis of flowering time under different nitrate conditions (Fig. 6, S7), it can be seen that ON conditions can induce the up-regulated expression of *ClCRY1a*, *ClCRY1c*, *ClCRY2a* and *ClCRY2c* genes, so that they could reach the expression level of short-day induction stage at the vegetative growth stage, which made up the required expression level of *cryptochrome* after *C. lavandulifolium* entering the floral transition stage in advance.

## Discussions

### Distribution and characteristics of ***RWP-RKs*** in ***C. lavandulifolium***

Predecessors have identified 14 *RWP-RKs* (9 *AtNLPs* and 5 *AtRKDs*) in *A. thaliana* [25], 37 *RWP-RKs* (18 *TaNLPs* and 19 *TaRKDs*) in wheat [17], 31 *BnaNLPs* in *Brassica napus* [19], 6 *SlNLPs* in *S. lycopersicum* and 14 *PtNLPs* in *Populus trichocarpa* [39]. In this study, a total of 101 *RWP-RKs* were identified. Compared with the number of genes in other species, the *RWP-RK* gene family of *C. lavandulifolium* underwent a marked expansion of members (Figs. 1 and 2). Besides, 56, 52 and 41 RWP-RKs were identified in *C. nankingense*, *H. annuus*, and *M. micrantha*, respectively, indicating that RWP-RKs have expanded in Compositae species. Combined with the phylogenetic tree, the *RWP-RK* genes had a specific distribution branch in Compositae species (Fig. 1), indicating that the gene has undergone specific differentiation, which may be involved in the worldwide distribution of Compositae plants in a short time.

Among the known identifications, the *RWP-RKs* gene contains two subfamilies, namely *NLPs* and *RKDs*. *RKDs* have a conserved domain RWP-RK, while *NLPs* have a RWP-RK domain and a PB1 domain, as well as a GAF-like structure domain [27]. Comparing the domains of *C. lavandulifolium*, it can be found that *ClRWP-RKs* is highly conserved (Fig. 3). *ClNLPs* contain two domains (RWP-RK and PB1), but some members (such as Cl30787.1, Cl40458.1, Cl60370.1) have only one RWP-RK domain. The deletion of PB1 domain at C-terminal, and it is speculated to be caused by the loss of sequence fragments or sequencing errors in the evolution of the *C. lavandulifolium* genome. No GAF domain was found in the *ClRWP-RKs* gene. In addition, the diversity of gene structure determines its function and plays an important role in the evolution of gene families [29, 35]. The *ClRWP-RKs* gene structure was diverse, and the sizes and positions of introns and exons of different genes varied greatly (Fig. 3c), indicating that there was structural diversity between the two subfamilies of *ClRWP-RKs*. Combined with the specific differentiation in *C. lavandulifolium*, the rich biological functions of *ClRWP-RKs* deserve further study.

### Differential expression of ***RWP-RKs*** in ***C. lavandulifolium***

*AtRKD1*-*AtRKD4* were highly expressed in reproductive organs compared with other species, and *AtRKD5* had pleiotropic effects on hormonal pathways [15, 31]; *AtNLP8* and *AtNLP9* were preferentially expressed in aging leaves and seeds, but were low expressed in other organs [5]. *OsNLP1* and *OsNLP3* were highly expressed in the source organs of rice [5]. *PtNLPs* had higher transcript accumulation in leaves, roots and male catkins of *Populus trichocarpa* [39]. In *C. lavandulifolium*, 44 *ClRWP-RKs* genes were identified in its leaves and apical meristems, and only 33 genes were expressed. The expression of *ClNLPs* genes was more extensive, especially in leaves (Fig. 4). The accumulation of *NLP* gene expression in leaves can store N to coordinate leaf expansion and photosynthesis, which could leaf growth [20]. The large distribution of *ClNLPs* subfamily genes in *C. lavandulifolium* leaves may play an important role in N assimilation during leaf development. *NLPs* also play a key role in response to N deficiency [20], and N deficiency treatment induces changes in gene transcript levels in a wide range of cellular and physiological processes [28, 33]. All 10 *ClNLPs* genes were negatively regulated by LN conditions (Fig. 5, S4), 3 were induced by ON conditions, and 6 were induced by HN conditions. Among them, Cl34639.1 (*ClNLP7*) was induced by HN, which was different from *NLP7* identified in other species that mainly regulates nitrate response and N starvation response [4], suggesting that the specific distribution of *ClNLPs* in *C. lavandulifolium* leads to a different mechanism of N utilization in other species such as *A. thaliana*. Combined with the differentiation relationship of *C. lavandulifolium* in the whole evolution (Fig. 1), *ClNLPs* subfamily has undergone a large number of expansion and sub-functionalization in the evolution process, prompting it to have different types of molecular mechanisms to respond to external nitrate/N environmental change. At the same time, combined with gene expression trend and promoter cis-element analysis, it was found that the expression of most genes in leaves increased after entering the flowering stage (Fig. 5a), which may be related to the existence of a large number of light response elements on the *ClRWP-RKs* promoter. In addition, there are a large number of hormone response elements in the *ClRWP-RKs* promoter (Fig S2), and it is also an interesting question worth exploring whether these hormone response elements connect the network between the *RWP-RK* gene and the hormone signal.

In conclusion, there were various expression patterns of *RWP-RKs* in *C. lavandulifolium*. Most *ClNLPs* genes were highly expressed in leaves, and some genes were highly expressed in apical meristems before and under floral transition stage. This provides a new insight for the follow-up research of the biological function of *NLPs* in regulating the development of flowering in response to changes in nitrate/N conditions.

### ***ClNLPs*** involved in regulating the flowering time under ON conditions

As a vital element of plants, the importance of N to plant growth and development has always been the focus of botanists. Previous studies have shown that nitrate/N plays an important role in regulating flowering [18, 36]. Under different nitrate concentrations, the flowering time of *A. thaliana* showed U-shaped curve pattern. The optimum nitrate/N concentrations promoted flowering, while higher or lower concentrations delayed flowering [18]. As one of the important characteristics of ornamental plants, it is of great significance to improve the flowering time of chrysanthemum to meet market demand. From the cultivation experiments, it can be found that the flowering time of *C. lavandulifolium* under different nitrate concentrations was significantly different. The flowering time was advanced under ON conditions, and it was delayed under LN and HN conditions (Fig. 6, S7), indicating that nitrate played an important role in the flowering process of *C. lavandulifolium*. There is no clear study that nitrate/N is involved in a single regulatory pathway in plant flowering, but more and more studies have shown that nitrate/N plays a key role in flowering. For example, N can affect the expression of the central oscillator *CIRCADIAN CLOCK ASSOCIATED 1* (*CCA1*), *LATE ELONGATED HYPOCOTYL* (*LHY*) and *TIMING OF CAB OF EXPRESSION 1* (*TOC1*) genes through the photoperiod pathway, and affect the normal flowering signal by interfering with the circadian clock output; LN conditions induce the expression levels of *CRY1*, *CO* and *GI*, and HN conditions inhibit expression levels [45]. Based on the analysis of extensive transcriptome data, genes in photoperiodic and autonomous pathways have great potential in regulating flowering time in response to N availability [36].

As a typical short-day plant, the genes of the photoperiod pathway of *C. lavandulifolium* play an important role in the completion of the floral transition [42, 43, 48]. Combined with the qRT-PCR results of the floral-related genes under different nitrate conditions, there were differences in the transcription levels of photoreceptor genes and integration genes in the photoperiod pathway of *C. lavandulifolium*. Among them, *ClCRYs*, *ClPHYC*, *ClGI*, *ClCOL4*, *ClCOL5*, *ClFT*, *ClFLC*, *ClSOC1* were up-regulated under ON conditions, and *ClTFL* was down-regulated (Fig. 5, S5, S6). As photoreceptors upstream of the photoperiod pathway, even under long-day conditions, the expression levels of *ClCRY1a*, *ClCRY1c*, *ClCRY2a* and *ClCRY2c* can reach to the expression levels at short-day induction stage after being induced by ON conditions. Moreover, the expression of downstream floral integration genes was also significantly up-regulated, indicating that the floral-related genes can respond to external nitrate/N availability. Compared with other floral-related genes, *CRYs* may have greater potential to respond to external nitrate/N availability. In addition, nitrate/N signal can complement the induction of light signal on the transcriptional level of floral-related genes, but cannot completely replace light signal to induce floral transition. However, there is no direct conclusion about how nitrate/N affect the flowering time of *C. lavandulifolium* and how nitrate/N signals mediate the flowering process.

**Fig. 7:**
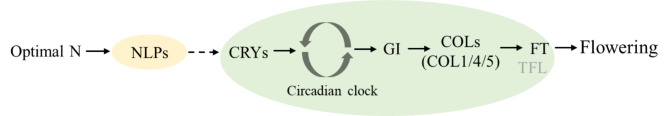
Prediction model of mechanism of *NLPs* regulating floral-related genes in photoperiod pathway. Black solid arrows represent known regulation, black dashed arrows represent predictive regulation, and gray genes represent inhibition of floral transition

In the above results, it can be seen that the expression levels of some *ClNLPs* genes (Cl34639.1, Cl04888.1, Cl60370.1, Cl06657.1, Cl20718.1, Cl61441.1, Cl17046.1) in the leaves were up-regulated when plant entered the floral transition stage (Fig. 5a). Among them, three *ClNLPs* genes (Cl04888.1, Cl60370.1, Cl62000.1) were induced to be up-regulated under ON conditions, which was basically consistent with the expression patterns of photoreceptor genes and floral integration genes. In addition, the expansion of *ClNLPs* genes in *C. lavandulifolium* also promoted the possible sub-functionalization of family members, and there were different types of response mechanisms to changes in the external nitrate/N environment. It is speculated that nitrate/N may first affect the flowering process of *C. lavandulifolium* by regulating the regulate the transcription level of photoreceptor gene *CRYs*, resulting in differences in flowering time of *C. lavandulifolium* (Fig. 7). This conclusion also provides key candidate factors for the subsequent exploration of the mechanism of blue light-cryptochrome-N nutrition.

## Conclusions

RWP-RKs in ***C. lavandulifolium*** showed obvious specific expansion. A total of 101 members were identified, including 38 *ClNLPs*, 31 *ClRKDs*, and 32 specific distribution genes. Among the 101 members 100 genes were located on 9 chromosomes of *C. lavandulifolium*. Then 44 *RWP-RKs* genes were identified in the transcriptomes of *C. lavandulifolium*, mainly *ClNLPs* genes. All 10 *ClNLPs* genes were highly expressed in apical meristems at the floral transition stage, among which 7 *ClNLPs* genes (Cl34639.1, Cl04888.1, Cl60370.1, Cl06657.1, Cl20718.1, Cl61441.1, Cl17046.1) in leaves were up-regulated after entering floral transition stage. The flowering time of *C. lavandulifolium* was advanced under ON conditions and delayed under nitrate stress conditions. The expression levels of 10 *ClNLPs* genes were all inhibited by LN conditions, among which three *ClNLPs* (Cl04888.1, Cl60370.1, Cl62000.1) were up-regulated by ON conditions. Photoperiod pathway *cryptochromes* (*ClCRYs*), *phytochromes* (*ClPHYC*), and floral integration genes were induced to express by ON conditions, while the expression level of *ClCRY1a*, *ClCRY1c*, *ClCRY2a* and *ClCRY2c* in the vegetative growth stage induced by optimal nitrate reach the expression level induced by short-day in the reproductive growth stage. It was speculated that *ClNLPs* may act on photoperiodic pathway under optimal nitrate/nitrogen environment, and ultimately regulate the flowering by up-regulating the transcription level of *ClCRYs* (Fig. 7).

## Materials and methods

### Collection of genomic data and identification of RWP-RKs

The ID of AtRWP-RK TFs was queried in the Plant Transcription Factor Database (PlantTFDB, http://planttfdb.gao-lab.org/download.php), and download the corresponding sequence as reference in the Arabidopsis Information Repository (TAIR, https://www.arabidopsis.org/) for identifying RWP-RKs in genome databases of other species. The genome data of *C. lavandulifolium* (GCA_022545495.1), *O. sativa* (GCA_014636035.1), *H. annuus* (GCA_002127325.2) and *M. micrantha* (GCA_009363875.1) were downloaded from NCBI (https://www.ncbi.nlm.nih.gov/genome/?term=). The RWP-RKs protein sequences of *L. sativa*, *T. aestivum*, *A. trichopoda* and *S. lycopersicum* were downloaded from PlantTFDB (http://planttfdb.gao-lab.org/download.php), the genome data of *C. nankingense* was downloaded from Chrysanthemum Genome Database (http://www.amwayabrc.com/download.htm). For member identification, the first round of BLAST (e-value < 1e-5) was performed using the AtRWP-RK sequence. All protein sequences were analyzed for conserved structural domains and motifs to ensure that they had similar structures to AtRWP-RK, in order to avoid errors in the annotation. ID of *C. lavandulifolium* transcriptome: SRR14723013 ~ SRR14723015, SRR14723028 ~ SRR14723033 [38].

### Phylogenetic tree construction and chromosomal localization of RWP-RKs

ML maximum likelihood method and 1000 bootstrap repetitions were used to construct the phylogenetic tree of RWP-RK by TBtools software [6], then the phylogenetic tree was visually partitioned using iTOL online software (https://itol.embl.de/tree/202204120156262211655887181).

According to the annotation file of *C. lavandulifolium* genome database, the chromosomal localization information of ClRWP-RKs were retrieved, and used TBtools to draw their location on *C. lavandulifolium* chromosome.

### Physicochemical properties analysis and subcellular localization prediction of RWP-RKs

The physicochemical properties of RWP-RKs protein were analyzed using Expasy ProtParam online software (https://web.expasy.org/protparam/), and the subcellular localization were predicted using Expasy Protscale online tool (https://web.expasy.org/protscale/).

### Gene structures, conserved motifs, conserved domains and promoter cis elements analysis

Motif identification of RWP-RKs was performed using Em for Motif Elicitation online tool (MEME, https://meme-suite.org/meme/tools/meme). The maximum width of the motif was set to 100 amino acids (aa), and the number of the motif was set to 10. The Conserved Domains Search database (https://www.ncbi.nlm.nih.gov/cdd/?term=) in NCBI database was used for RWP-RKs protein conserved domain prediction. The identified motifs, conserved domains, and gene structures were visualized using TBtools based on the known *C. lavandulifolium* genome annotation files and the RWP-RKs coding sequence.

Based on *C. lavandulifolium* genomic database, the 2000 bp sequences upstream of the coding sequence of RWP-RKs were searched. PlantCARE online software (http://bioinformatics.psb.ugent.be/webtools/plantcare/html/) was used to predict the cis-acting elements of the promoter sequences, and TBtools software was used to visualize the cis elements composition.

### Plant material and treatment

The plant materials were taken from the tissue culture room of Beijing Forestry University, and were the strain for *C. lavandulifolium* genome sequencing [37, 38]. When the tissue culture seedlings of *C. lavandulifolium* grow to just take root (5–6 leaves) in the bottle, selected that with consistent growth move into 10 × 9 cm plastic pots and transferred them into the long-day artificial climate chamber, one plant per pot. The cultivate substrate was perlite and nitrate treatments with different concentrations were started after transplanted for 7 days. Nitrate was provided using potassium nitrate (KNO_3_), with 0.1 mM KNO_3_ for low nitrate conditions (LN), 1.5 mM KNO_3_ for optimal nitrate conditions (ON), and 3.0 mM KNO_3_ for high nitrate conditions (HN), total of 3 treatments. The contents of other elements referred to the modified Hoagland nutrient solution. They were treated once a week until flowering, and the flowering time under different treatments was observed.

The temperature of artificial climate chamber was 24 ± 2 °C, long-day conditions was16 h light/8 h dark (16 h L/8 h D) and short-day conditions was 12 h light/12 h dark (12 h L/12 h D), respectively. The light source was incandescent lamp with an average light intensity of 3000 lx. After grown to 14 leaves, they were uniformly placed under short-day condition for short-day induction [48].

After cultivated with conventional water and fertilizer management, collecting the organs (leaf, stem, root, apical meristem) at the vegetative growth stage under long-day conditions and the organs at the reproductive growth stage under short-day conditions. For different nitrate treatments, the leaves at the vegetative growth stage were collected after four treatments for analysis the key gene expression response to nitrate. After harvested, all samples were quickly frozen in liquid nitrogen and then transferred to an ultra-low temperature refrigerator for subsequent analysis.

### Expression analysis of *RWP-RKs*

Used the RNA-seq database of reproductive-stage leaves, vegetative buds and reproductive buds of *C. lavandulifolium* [38], the *RWP-RKs* genes were screened. The TBtools software was used to construct heat map based on the fragments per kilobase of transcript per million mapped reads (FPKM) value.

Total RNA was extracted from the collected plant materials using Plant RNA Rapid Extraction Kit (HUAYUEYANG Biotechnology, Beijing, China) and treated with RNase-free DNaseI to digest DNA. Gene expression analysis was performed by Real-time quantitative reverse transcription-PCR (qRT-PCR), which performed using a Mini Opticon Real-time PCR System (Bio-Rad Laboratories Inc., Hercules, CA, USA) based on the SYBR Premix Ex Taq (Takara Bio Inc., Shiga, Japan). *SAND* was used as internal control gene for qRT-PCR with three biological replicates [26]. The primers for qRT-PCR were shown in Table S4.

### Statistical analysis

Each organ of *C. lavandulifolium* was harvested at least three repetitions. qRT-PCR experiment was performed in three biological replicates. The data processing was performed using Excel 2010 and SPSS 20.0. Photographs were created using Origin 9.0 and TBtools.

## Electronic supplementary material

Below is the link to the electronic supplementary material.

Supplementary Material 1

Supplementary Material 2

## Data Availability

The databases used in the study includes Plant Transcription Factor Database (PlantTFDB) (http://planttfdb.gao-lab.org/download.php), the Arabidopsis Information Resource (TAIR) (https://www.arabidopsis.org/), National Center for Biotechnology Information (NCBI) (https://www.ncbi.nlm.nih.gov/genome/?term=), Chrysanthemum Genome Database (http://www.amwayabrc.com/download.htm), iTOL (https://itol.embl.de/tree/202204120156262211655887181), Expasy ProtParam (https://web.expasy.org/protparam/), Expasy Protscale (https://web.expasy.org/protscale/), PSORT Prediction (http://www.genscript.com/psort.html), MEME (https://meme-suite.org/meme/tools/meme), Conserved Domains Search database (CDD) (https://www.ncbi.nlm.nih.gov/cdd/?term=), PlantCARE (http://bioinformatics.psb.ugent.be/webtools/plantcare/html/). The public access to all these databases is open. The data that support the findings of this study are available from the corresponding author upon reasonable request.

## Abbreviations

N: Nitrogen
NLP: NIN-LIKE PROTEINS
RKDs: RWP-RK domain proteins
PB1: Phox and Bem1
NUE: nitrogen use efficiency.
