# Supplementary material for: Genome-wide identification and characterization analysis of RWP-RK family genes reveal their role in flowering time of Chrysanthemum lavandulifolium
Source: BMC Plant Biol. 2023 Apr 15;23:197. doi: 10.1186/s12870-023-04201-2 (PMC10105424; doi:10.1186/s12870-023-04201-2)
Supplement: Supplementary file 1 — Supplementary Material 1 [file 12870_2023_4201_MOESM1_ESM.docx]

>Cl47376.1

MCSLYTHQSSYLFTNTYSLSIFKLMEFSREVITQYFNIPIARAAKEFNVGLTIFKRNCRDVGIQRWPCRKLMSLQALINRFQVDNGGAKAKGSTEEIIRTPKEQKRRCRRKPDFRFPKVPKVETKTIYPTSESDYEEMDDSYGSDYDEKSQINIDLEM

>Cl55847.1

MHGSVHHEVGPLLHSAFQEGILDSFAMIPICCPSQTSSSSDCIGVIECSSWYFQSDVELFNQMNMTIKELGLGVYSVQDRIPYKSINGLKHATDQIQEALKIVCQSHQIALAQVWIASLDESHVHFSSSWEEAQTSQLLGLKLTGYKSNDENSEIKYRKFRTYQDACDYFPLQPGQELVMKTFQDYKPRFCKNISQLVTYKLMGLRSAAVDGTKSLTICMRSIHTGDLNYVFEFLWPDNSFNPILLEEILLNIKRCLPSFKFASSTEIGDKLDVIEVDNSTDKEIKKFDIFQSKRLSPIPEALKEGKKQVAVDYIAPLDTKCKIPPVLIPQQVIEQQFDTSIKEDFLENIFGNRNLWNDPDDERLSEFLETLPTYILHKKPETEGALWVFCSKDEGSQNNSSSDVLGTRMIYDKIKSAFSKVSNSGELIVQFWAPVTSGNRLVLSTSGQPFAVSDFGEGLKEYRGRCVGYECNIDMNNNNKVIKDENEQH

GDRHPMTMISGPPTNAFLNHLPEAVLDTKNHREGSLMRYASDCGVWTSYSLPIGCPSQYDSCIGVVECSSISSINLEIVNTMNRALEQEGLNVFNVQDRIPYNTINDLTLVRDEIQVAMKSVCESHKIGIAQVWISYEDENYMPFSFSSEDTQTKRRLALKLTGYNSVNEDSTACHLRFKEYCDACDMVPLKMGEELVEKTLQDYQPRFCENISQLGTDTLMAWISTDDVACSGFTICMSIDTGDFSCAFEFIWHHNPDYVLLLEALLLTLKRHLPRFKFASGAEPDQLRVIDVENSTKSETKSFKIFKEKQLSPIPEAMNKGKKAIVVNYNAPIPEAMNKGKKAIVDNYNAPIPEAKNKGKKAIFVNYNELSKEKRVTKEIELSREEIEQHYGKTEKQAAKELRVSLSTLKRKRNKLGMSWWQGPDLPQRKAYNSNKNRNKESHTHEKDNGVIQDPSSVKRNENIVIKAEYADDIIKIRLGNSEATFVTVENEIGKVFKLKHGILRSSTLMKMKSGY

>Cl20718.1

MDGRSSVSSYKKSSYSGSHSVSTDKTDVWSKVSRTDGSRYTWVSRNDKYRAIVRIRAVKARHVVWSRVTGRHTTDGSVANCYRRDSNTVDKDDDKDVSARVRGWTSDITNYKHYDSAIRCNHGYAVDSTTRCAGVMSSKNSSYAVAHKVKMNTISVDSTTKVTCRDVKIGIKSVCDTHNATWAVSSTSVSHDKIIDKSCGSDIKCIGKICMHATDHVRDGTWRACIHDKSCSVGRAARGSSYCDVTTYAHNAHVSKITSCAIHSGNGYVYMKDGSYVYVTKKVDASGKGDISSTGRDAGYSSNIKSIISTATTANTAGMSSDSGSANVVTDSTNVSCSSKHDNDMSDNVNMAGSRDDNANYSIVINTTSDTITDARNSIKRGRKRKIDSSMKAIHVGKISVASGVSKSTKRCRNGISSWKIRAKTCHATDSRCK

>Cl16033.1

MDGRSSVSSYKKSSYSGSHSVSTDKTDWSKVSRTDSGSRYTRWVSRNDKYRAIVRIRAVKARHVVWSRVTGRHTTDGGVANCYRRDSRNTVDRDDKDVSARVRGWTSDITNYKHYHSAIRCNHGYAVDSTTRCAGVMSSKNSSYAVVHKAKVTCRDVKIGIKSVCDTHNSATWAVSSTSVSHDKIIDKSCGSTKCIGKICMHTTDHVRDGTWRACIHDKSCSVGRAARGSSYCDVTTKYVHNAHMSKITSCAIHSGNGYMKDGSYVYVTKKVDASGKGDISSTGRDAGYSNMKSIISTATTANTVGMSSDSGSANVVTDSTNVSCSSKHGNDMSDNVNMVGSRDGNANYSIVINTTSDTITDARNSRKRGRKRKIDSSMKAIHVGKISVASGVSKSTKRCRNGISSWKISKKTCHATDKMSVVSSSRSSSGMHSCTSKSDSIYAAYMVKAIKDDMIKRITSGNVARDKGKRSIKYKDNDITCDDDHSRNARNTTAKVDSN

>Cl70290.1

MDYRTTTMASVKNDDVVNMSDDAWNSNMSCSTMDSSSYDSADVYMTCDNVCTIDMKDYSDDRIVIYNRCVDTDVWVVRNGRVTTNMVNSDSTNSCYRVSKNYAAYDSNTIGGRVKTTITRRRRRRVTHANSIGCNSDGTCVGVVVVTTSNVNRASIKADIHRKKGVRSICDTKATWGSKGRSRDVDSISIISASYVDIDACSIMGGIAGKAGTNCTNIDDCRADYGARMGNCAIIRHSTYTGDNMRNSIASMTKVGACVIKGVVASIKTNTSDSWISHMANRGKTSCWDVINYYHTAKNTYIWGSKSRIASSGTKRTGKSRVKTRNISVYGSKDAAKSIGVCTTKRICRHGIMRWSRKIKKVSHSKKVIDSVGADGMKGSYTNNSISKKVNDRVNHKTATSNSSSSCSRDSSSSTGNAIHTAKTRGVHTVRSNVIVHAKKSSVSNDSVRVKASYGDKIRKMSKHWGGDHRITKRNIYDMGNITYIDDSWVACDADCMDHTSTKNTIKVHSSHHSC

>Cl20158.1

MDYRTTTMASVKNDDVVNMSDDAWNSNMSCSTMDSSSYDSADVYMTCDNVCTIDMKDYSDDRIVIYNRCVDTDVWVVRNGRVTTNMVNSDSTNSCYRVSKNYAAYDSNTIGGRVKTTTDRVAGSDRVTHAKNIYGCNYDGTCVGVVVVTTSNVNRASIKAVVDRSSDIHRKKDSYVAIRDVRSICDTKATWGSKGRSRDVDSISIISASYVDIDACSIMGGIAGKAGTNCTNIDDCRADYGARMGNCAIAIRHSTYTGDVYSWSHVIKDVVASIKTNTRDSWISHMANRGKNVISMGCHKDVINYYHTAKNTYIWGSKSRIASSGTKRTGKSRVKTRNISVYGSKDAAKSIGVCTTKRICRHGIMRWSRKIKKVSHSKKVIDSVKISKKVNDRVNHKTATSNSSSSCSRDSSSSTGNAIHTAKTRGVHTVRSNVIVHAKKSSVSNDSVRVKASYGDKIRKMSKHWGGDHRITKRNIYDMGNITYIDDSWVACDADKADVHGNVDTDVTSNDCHANTRRKRRASDDACINIIIMTVNHMKRANDADGSRHVACNSR

>Cl29689.1

MASRVIAYDKITKVVKGNIGTVKRCRDGIRWHRKKSIAIDSIAYNGGAHDYIIRTKKKIKNDARDTKRWRVNKAKYKRKVMAISTNKTATNVDTVSSKNDATIDIDGYCKHCAD

>Cl85994.1

MKAANIDKGDVSDTTCIHKKTGAWVCSDGSKNSSGDGTRMIHNRIKSASKVSNGGIVWAITSGNRVTISGAVSVGGKYRGRCVGYCNIDINNDNNKVIKDDIHDHHMTMISGTNANRAVDTKNHRGSMRYASDCGWTSYTIGCSYYSSCIGVVCSSISSINIVNAMNRAGNVNVDRMYNTINGKVRDIVAKSVCSHKIGVAVWISYDNHVSSSDTTTRRAKTGYNSVDDSTAHWRKYYDACDMVKKGVKTDYRRCNISGTDMMAWVSTDDVACSGTICTSIDTGDNCAIWHNNYVATKRCRNASGANGDHVIDVDNSTKSTKKIKKRSIAINKGKKAMVVNYNARSKVKRDTTISRDIHYGKTMKAAGVSSTKRKHNKGMSGWGDSRKAYNSNKNRKSHTHKDNGVIDSVKRYNTVIKAYADDIIIHIISATVTVYIGKKKDGTKIKDDWIMTSDDSYCTNRNVDRSVVRRVHN

>Cl89122.1

MADSGDDTGSTYRHSKRSGDGWVSNKDGYSNSYSSDDVGTRMMIYDIKSASKIHNNYGIAWAVTIGGKSTSGAVNYHSKYRRRCVYYDIDVNSKRGTASANRVIAMHGRVYHVDRNACSDSSVMIICSTSSSDCIGVICSSWHSRAINMNMKIKVGGVYNIDRIYKTINGKARDIAKIVCSHIAAVWIASDNHVHSSSWATRGKTGYNNIDDSAYCIIYHDTCDMIVMKTDYRCNISVTYKIVGIAGVNGIRSTICMRSIHTGDNYVWNHNSNIIIKRCSKAGTIGDKHVIDVDKSIGKINKKDIKRIAKGNKVAMDYIVDTNCKTAVIVIGTSTTKNVINTSMNAANICNRNWNDDDRSTTYIHKKKTGGWVCSKDGSHNISSGDGTRMIHDKIKSASKVCNGGIVWAITSGNRVSTSGAVSVGGYRGRCVGYCKTNMNNDNNKVIKNDIHDHHMTMISGTNANRAVDTKNHRGSMRYASDCGWTSYTIGCSYYSCIGVVCSSISSINIVNTMNRAGNVNVDRIYNTINGTVRDIVAKSVCSHKIGVAVWISYDNHVSSSDTTTRRAKTGYNSVDDSTVSNWRKYYDACDMVKKGVKTDYRCNISGTDTMGWVSTDDVASGTICMSIGTGDNCAIWHHNNYVAMKRCSKASGAGDHVIDVDNSTKSTKKIKKRSIIDKGKKAMVVNYNARSKKHDTTIKSRDIYGKTMKKAAKDGVSSTKRKYNKGMSGWGDRKAYNNKKRNSHTHKDNGVIDSVKRNITVIKAYADDIIKHRISATVTVNIGKKKKHGTKIKYDDWIMTSDDSDCINSRTVDRSAVRCVHN

>Cl66641.1

MADSRDKKYSGSTYRHSSGDGWVSNKDGYNDVGTRMMIRAKISAYKIHNDHVNSYVIAWAVTIGGKHSTSGAVNNGSNYKKYRRRCVYYDIDVNSKRSTASANRIASMYTSVHHMDRYAHCGRDSMMIYCSTSSSCDCIGVICSSWTNCTGIINMKIKVGGVYNKDHIYKTINGKVRDIAKIVCSHHITAVWIASDNYMHSSSWATRGKTGYNIDDNRCWNYYDACDIGIKTDYRCKNISKGYKMGWWNWYAATDRSMGTICMRSIHTGDNYVIWKHNNSIIKRCSKASGTIGDKHVIVNSSTDKIKMDIKRAIGAKGKKVAVDYIADTNCKTTVVVIGTSTTDAANIGGDRMADRDDRSSMNKGKKAMVVNYNAHKKRDTSKKSRNIKHYGKTKAAKDGVSSTKRKRNKGMSGWGDSHRNAYSSNKNSKSHTHKDNGVIDSVKRNNTVIKAYADDIIRHISATVTVNIGKKKHGTKIKYRDDWIMKTDDNDCISSRNVDRSVVRRVRHN

>Cl78296.1

MADWKGSGSTYRHSKRSNGWISNKDGYSNSYSSDDVVTRMMIYDKIKSASKMNYHSGIIIWAVTIGSKHSTSGAVSYGVKYRRCVHVYDIDVNSKRGTASANRIAYKHSVHHVDSNACCTISIMMIYCSTSSSCDCIGVICSSWTKCTDIIMNMKIKGSMRYASDCGWTSYTIGCSYYSSCIGVVCSSISSINIVNTMNRAGNVNVDRIYNTINGTVRDIVAKSVCSHKIGVAVWISYDNHVSSSDTTTRRAKTGYNSVDDSTVNWRKYYDACDMVKKGVKTDYRCNISGTDTMGWVSTDVASGTICMSIGTGDNCAIWHHNNYVMATKRCRNASGTHVIDVDNSTKSTKKIKKRSITVDKGKNSVVVNYNVHSKKHDTTIKSRDIHYGKTMKKAAKDGVSSTKRKHNKGMSGWGDRKAYNSNKKRNSHTHKDNGVIDSKRNNTVIKAYADDIIKHRISATVTVNIGKKKKHGTKIKYDDWIMTSDDSDCINSRTVDRSAVRRVHN

>Cl27852.1

MKIIRIHVIDRNAGSIHHHIYYVGTRMMIYDKIISASKMNYHHGIAWAVTIGGKHSTSNAASYGSSKYRRRVHYDIDVNSNNSKRGTASANRIASMHGSVYHVDHYACSMTSMIICCSTSSSDCIGVICSSWTIDRVHNSMNMKIKVGSVYNVDHIYKTINGKARDIAKIVCHITAVWIASDNHVHSSSWATRGKTGCIDARNCWIHYYDACDSGVKTDYRCKNISHGIYKMGWHWSASMGTICMRSIHTGDNYVWKHNSNIINIKRCSNASGTIGDKHVIVDDSTYKIKKDISKRSIAKGKKVAADSIGTKCRAVTVIGISTTNVTVIGISTANVTVIGISTTNVAANIGNNMADRDDRSTSYIHKKTGAWVCSDDDGTRMICDKIKSASKVKNDGIVWAVTSGNRRVSTSGAVSVVGYRGRCVGYCNIDMNNDNNKGNVNVDRIYNTINGTVRDIVAKSVCSHKIGVAVWISYANHVSSSDTTTRAKVTGYNSVDHSTSSHWRKYYDACDMVKKGVKTDYRCNISGTDMMAWVSADDVVCSGTICTSIDTGDNYAIWHNNYVATKRCRKASGTNGDHVIDVDNSTKSTKKIKKRSIAINKGKKAMVVNYNARSKVKRDTTIKSRDIHYGKTKAAKDGVSSTKRKRNKGMSGWGDSRNAYSSNKNSRSHTHKNGVMDSVRNIKTTVTVNIGKKKHGTKIKYRDDVDINN

>Cl72293.1

MKAANIDNGNMADRDDRSTTCIHKKTGAWVCSDGSNNSSSDVGTWMIHDKIKSASKVGNSGIVWAVTSGNRVSTSGAVSDVAVVYRGRCMGYKCNIDMNNNNKVIKDIHGDRMTMISGTNANRAVDTKNHHGSMRYASDCGWTSYTIGCYYSSCIGVVCSSISSINIVNTMNRAGNVNVDRIYNTINGTVRDIVAKSVCSHKIGVAVWISYDNHVSSSDSTTRRAKTGYNSVDDSTSNWRKYYDACDMVKKGVKTDYRCNISGTDTMGWVSTDDVASGTICMSIGTGDNCAIWHHNNYVATKRCRKASGAGDHVIDVDNSTKSTKKIKKRSIIDKGKKAMVVNYNARSKKHDTTIKSRDIHYGKTMKKAAKDGVSSTKRKHNKGMSGWGDSKVYNSNKKRNSYTHKDNGVIDSSVKRNNSVIKAYADDIIKIRGNSATTVNIGKVKKHGNKIKYDDWIMTSDDSCINSRRRVHN

>Cl05308.1

MRGSTTCIHKKTGAWVCSDGSNNSSIDDGTRMIYDKIKSASKVNNGIVWAITSGNRVTSGAVSDGAVYRGRCMGYCNIDMNNDNNKVIKDIHGDRHMTMISGTNANRAVVNTKNHRGSMRYASDCGWTSYTIGCSYYSSCIGVVCSINIMASINKIVNNMNRADGNVNVDRIYNTINGKVRDIKVAKSVCSHKIGGAVWISYDNHVSSSDTTTRRAKTGYNSVDDSTAHWRKYYDACDMVKKGVKTDYRRSNISGSDTMGWVSTNDVACSGTICMSIDTGDNCAIWHHNDYARKASGAGDHVIDVDNSTSTKKIKKRSIAIDKGKKAMGVNYNARSKKHDTTIKSRDIHYGKTMKKAAGVSSTKRKHNKGMSGWGDRKVYKSNKNSKSHTHKDNGIDSVKRNNSVIKAYADDIIKHRISATVTVNIGKKKMKHGTKIKYDDWIMTSDDSYCINSRNVDDCVCYTTNSRSCMCSI

>Cl70883.1

MSGSGDDSGSTYRHSKRSVWVSSKDGYNNSYSDDAGTRTMIYDKIKSASKIDYYIAWAVTIGGKSTYGAVSDSSKYRRRCVYNDIDVNCNNSKRGTASVNRITISMHTIVHHVDWNACGTTSMMIYCSTSSSCDCIGVICSTWTNCTDIMNMKMKVGGVYNVDRIYKTINGKARDITKIVCSHIAAVWASDNHVHSSATRRGKTGYNSDDNAKDKWTRNYHDACDIGVMKTKDHKRCKNISVTYKMGGSADGIKSTICMRSIHTGDCNYVWNHNSYIKIKSCSKYASGTIGDKHVTVDNSTDKVKKDIKRSIDSMNKGKKVMVVKYNASKKRDTTIKSRIHYGKTMKKAAGVSSTKRKHNKGMSRWGDRKAYNSNKNSKSHTHKDNGVIDSVKRNNTVKAYADDIIKHRISATVTVNIGKKKKHGTKIKYDDWIMTSDDSDCINRNVDRTAVRRVHSHVNSVMTAGSIAIDMIKYCHKRHSHTVNCII

>Cl52612.1

MVDNDKDSGSTYRHSKRSCSVWVSSKDRYNNSYSDDAGTWTMINDKIKSASKMNGHIIAWAVTIGGKHSTSGAVSYHKDIKYRRRVHVYDIDVNSNNSKRGTASANRGSIVAIIYRYTSSSSDCIGVICSSWKWVWVYNVDRIYKTMKGKVRDIAKIVCSHNIGVAAWISYDYHVSSSDTTTRRAKTGYNYYYSTASSRNYYACDMVMGGVKTDYRCNISGYDTIGSVSTYRACSGTICTSIDTGDYNCAVWHHNNYVATKRCHNASGAGDHVIDVDNSTKSTKKINKRSSIKIDNGKKAMVINYNAHARSKKRDTTISRDIHYGKTMKKAAKGVSSTKRKHNIGMSGGDRKAYKSNKYSKSHTHKDNGVIDSSVKRNNTDIKAYDDIIKIHGNSATVTVNIGKKKKHGTKIKYRDDWIMTSDDSDCINSRNVDRSAVRRVHN

>Cl37580.1

MKAANIDKGDVSDTTCIHKKTGAWVCSDGSKNSSGDGTRMIHNKIKSASKVSNGGIVWAITSGNRVTTSGAVSVGGKYRGRCVGYCNIGINNDNNKVIKDDIHDHHMTMISGSNANRAVDTKNHRGSMRCASDCGWTSYTIGCSYYSSCIGVVCSSISSINIVNTMNRAGNVNVDRIYNTINGTVRDIVAKSVCSHKIGVAVWISYDNHVSSSDTTTRRAKTGYNSVDDSTSNWRKYYDACDMVKKGVKTDYCCNISGTDTMSWVSTDDVACSGTICTSIGTGDNCAIWHHNNYVSTKRHRMKASGAKGDRVIDIDNSTKSRKIKKRSIAMNKGKKAIVVNYNASKKRDTKISRDIHYGKTMRKAAGVSSTKRKHNKGMSGWGDRKAYKSNKNINSHIHKDNGVIDSVKRNNTVIKVYADDIIKIRYNSATVTVNIGKKKKHGTKIKYRDDWIMTSDDMSDCINSRNVDRSAVRHVHN

>Cl18769.1

MADWGSSTYHNNNSDWVSSKDGYSNSYSDDVVAMMIYDKIKSASKMNCYCAIAWAVTIGGKHSTSGAVSCTSAKKHRRRCVHVYDIDMNSKKGTVSANRITISTSVHHMDRYACGSNSMMIYCSTSSSSDCIGVICSTWCSVNNMNKIKVGSVHNADRIYKTINGKARDIAKIVCSHITAVWIASDNHVDSSSATRRGKTGYKNIDDTIDDTIDDSIDNNRCNYYDACDIGIKTDRCKNISKGIYKMGDWCGGTDKSMGTICMRSIHTGDNYVWTHDSNIINIKRCNKASGTIGDKHVIVDNSTDKIKKDISKRSIAKDGNKVAVNYITDTNCKTAVVIGTSTTDGSRNASGAGDVIDVDSSTSTKISKKRSIAIDKGKKAMVVNYNTRSKRKRDTTKKNSRIRHYGKTMKAAKGVSVSTKRWHNNCKSGWGDRKAYNSNKNSKSHTHKNNGVIDSVKKNNTVKAYADDKIIHISATVTVKNIGRKDGTKIKDKDWIMDSDDSYCINSRNVDRSARVHN

>Cl19482.1

MDTNAIYSHRYHMHDNDTISTSIDMGDDDVDDSIYNDDGTNTTVNDTNVGSSGNIHSSWVYTCSCCNRISHTNGVDITKIHGRGVISHAVKYSVDTTNTHKYKMDCKSIVRVKVYCNRKTNGYIMDSSYAVCVGDWDNTVTDDIDDDSGHMIISDVTSGARSNRSTSIRRTKTMKDTVYHIIAAAKKINCTVIKRVCRKHGSRWYRKIRSIKKISMRATYTSVDVKRGRAAIDTRIANIYSIYNA

>Cl78287.1

MHDNDTISTSIDMGDDDVDDSIYNDDGTNTTVNDTNVGSSGNIHSSWVYTCSCCNRISHTNGVDITKIHGRGVISHAVKYSVDTTNTHYKMDCKSIVRVKVYCNRKTNGYIMDSSYAVCVGDWDNVTDDIDDDSGHIIISDVTSCGARSNRSTSIRRTGKTMKDTVYHIIAAAKKINCTVIKRVCRKHGSRWYRKIRSIKKISMRATYTSVDVKRDSNMKSNMSYSTVCVKVKAVWDDSMGDIIGIAADGCTGVDKVIKGKSKSRRISKRNADGVWKVRRGDMVNMRVCAVKRRRKSRAVSNKHVAVDDDDCTMSRKSVSIVSRGNAIKYNWRRKAATYDHRSTVVSSIRANHDIDSITIRAMAWSAAVSSGTIVVNITIISSDKGNSTRTHGVRWGIIIISGNRGIDIKRTDGVCYAVTKDSAADRAGRKNATTGHMVVSRGKSVMTMVGSDSHCCDNSDIRTVGAVDTIHIMSWTTSGSRTVGATRMS

>Cl38693.1

MDHACHSVDSRDTSCSGSSVSKKITWNKSRTVSSGMRYTWVSRNWDYRADNNVHNKKKIRAAKTRRVVWSRVVGKHTAIDGGVIGCSYRRDSRNVVDKDHADISARVRGWTDINYKDCAIRCNHGYAVDSTTNCVGVIMSSKTTYAVVHKAKKSTSVDAINVSNRIDDICGIRSVCDSHSAAWAVSSASVSHDNIIDKTCSSDTKCIGKDCMSTNGHVDMGMWRACKHHDKSRSVGKAARGSKYCDVTTYVKAHNYKIASCAIHSSGNDYVYKDGSYVYVTKKVDASGGISTVTGKDASSSSIKYTISAVTAANTYGMDSSYSVANVAKTDSADVSCSSRNYNDMSDNINMVGSRIVNATSYSIVTNKMSDTTTNAGVKSTTKRGRKRKIDSRMAVHVGKINAASGVSRSTKRCRNGIMSWKSSTKSSHATDKMSVVSSSRSSSGWARRVVVIRVKRSSGRHSYSKSGSIYVATVKATKGDMIKRISSGSKNVAHRNKGTKSIRYKDKDDIITCDDDHTASNTTVKVACE

>Cl40458.1

MACSTMSAGWTDTRSVYWNKVSGSIKSSNSISIVVNIWYNSDIVTNVKKKIISIARNSVNICWAKKIGKSIVTTVCGSGVNKVYRKGCKYKYVHGKAVGAGRVNCIHTDMHNYKDRCDAISRIWGSIAVVINGCVVVDTMDSYDNIIAVCRNAGSSITDYSRRICINTNINGRKRMRRTRSHTYYRVYGMSSADAAMGIKKGTRNVCRNVGIWGIKNTRAASAIVSTISDTSSCGTVDDAVSSRNGTSSRATTVKASNHVNTVTSNANDSCWWDGGVYGTIITNTYATINIVDTSDADSHDGIYGNTMAIDDHRVDSDYIDMMTWAA

>Cl73932.1

MISNSGCCVGVVTSSSNYVDYAVISRAKNKSNVDIYKVSNRRCAIGIAKTVCDTHKATWSSGKNSVANSGTCSSKKSCIGKICMSTANYVRDVSMWGRACIRHKSRGVVGRSTCGSWCKDVTDDDYVARMSRTSCAIYIKSIASYVITSTNDIVKTVKIKKVKCVTSTNIGAVNISSTIAVSVRMSVNINSNSGVVGACVVYKGVTYRKRKSSSSISIKKHGKVGAAAYNVSRSTKRICNDYGIRWYKMKRTDISIGTSTMKVIDRVRMSTSWMSTSDSSCNSGGVGTSHGVVYWKKRTTRRRKRSRVITISHTGKIDGATAINDSISMYWHGDRGTRNTHDATVAHGKNSTHHKNMDVRAKTSVIYITTAANTVKDTVKATYNTVKISNGAKKIATRRSGSKKYDKGDMIVACDTDASVGDSRVDTVIRVIDRSS

>Cl38225.1

MSSRDVVSHYHMHRNAKCNVGSSKVHCRSVGIKSWYRKSKINYGKDGDSNTNNMIITKKRRIKDAITNNTKKRRRRATYRKKKHINSAIVDVVIYNNHASYDISYGSNYKQ

>Cl12565.1

MAYWSDTYRHSKRSRSGWVSSKDGYSNSYSDDVGTRMMIYDKIKSASKMDNRYGIAWAVTIGGKSTSGAVNYKDMKYRKTCVYGYDISIRGTASANRIIAMHGNVYHVDHYDCSTTSMMIYCSTSSSSCIGVICSSWHTNVNMNMKIKSINGKATDIAKVVCSHIDAVWIASDNHVHSSSWASRRSGKTGYTSIDGNSARNCWINYYDACDIGVKTHDYRCNISHGNYKMRWGVDGIRSTICMRSIHTGDNYVWNHNSYIIKIKKCSKASGTIGDKHVIVDNSTDKIRKDISKRSIAKGKKVAVDYIADTNCKTADVIIGTSTTNAANIGNGNMADRDDRSKTTYMHKKTGAWVCIDDGSYNSSSDDGTRMIYDKIKSASKVNNGGIVWAVTSGNRRVSTSGAVSDGAVYRGHYKIHGDRHMTMISDANSNRAIDTKNHRSSMRYASDCGWTSYTIGCSYYSCIGVVCSSISSINIVNTMNRAVDNVNVDRISYNTIKGKVRDIVAKIVCSHNIGVAVWISYDNHVSSDTTTRMAKIGYNSVNSTACHWRKYYDACDMVKKGVKTDYRCDISGTDTMAWSTDDVACSGTICMSIDTGDNCAIWHHNNYVATKSRRKASGAGHVIDVKSTKSTKKIRKRSTIAMNKGKKAMVNYNAKRDTTISRDIKHYGKTMRKAAGVSSTKRKHNKGMSGWGYRKAYNSNKNSNKSHTHKDNGVIDSKINNTVIKAYADDIIKRGISDTKVTVNIGKKKKHGTKIKYDDKWIMTSDDMSDCVSSRNIVRRVHN

>Cl33400.1

MNGSTTTISNITDARHNRSNITSNTVIAYHIAACKNVGTVKCRDKGKRWYRKRSDISTNGGAIVDDYTKVIITRKRRRVNNYKSTTKKRRRNKARYKRKHRTTTIATRVIYSNISDSKMDGDYDGDNTD

>Cl69407.1

MSFSREVISHYFHMPLHQAAKYFNVGLSVFKVHCRSVGIQCWPYRKLPSLERLINGFQVSNQLTLTFDSEFHTTSLF

>Cl63428.1

MSFSREVISHYFHMPLHQAAKYFNVGLSVFKVHCRSVGVQCWPYRKLLSLERLINCFQGKVGDSKTNEKMQEIITKLQEEKRRIEENPELPITKNTKKLRQRLFKANHIKINLELSLATPIATDVEPLQVIYPNNHASYEEIEFPYGSDYEEQ

>Cl75974.1

MDDGSTKTISMGAIKSSISVDDKSYDSSNSDSSSSYHSYNSRCDDTDVKRTSVDYTDTNDTKRTAIKYNTRDTIIWVRKGGCVTTDINSNSKVGYRHISKSHATDDSAGSVKKMCTGGKDVNSNMNCGVVDVRSGTCGVIIVTISNVNYRHDNISKAADRSSIHKDSNGSYVVAIRVKSVCDTHGATWSMCAGRGGCSAACISVIKSASYVDNVGYACSHGGIAGKAGTNCATDITGSRAYSDHAKGSGAVAIRRSTYTGTDIRDCRNDDAKMSSISYVIVSKSHVNNDIDSYSVKGSDSSWISYMMARGSVVSMGHKNVINWDYRSTSGRGKKIDKKGRKRGIKRSDKKRIKAKNISVYGSKDAAKSIGVCTTKRICRHGIMRWSRKIKKVGHSKKIIDSVGAGTIGSYTNSSTKTNDSAHASKTSGSSSCCSDISGGISKVNTYAKSGHNKADGKSKKDVRVKVVNGKKIRMCMSRDWNRAIMMRNIDVMNSVTKYDDDSWVTCDADCMDINIASKKCTINSHSRSSTGNGSY

>Cl62862.1

MIADDVNTDKKKKRDIDSDAKIDMTADYDGTSAIVKKKMDWHIKCMKTDVSKNGKMCINDCINIGITIKKRCRDGIRWYRKTSIINTIANRGAKVDGNTIITRKKKCVNDTKSTKRRHIYKSKYKRRKSKSISTNITSAATHYSYSSSAH

>Cl45481.1

MTDNKSNNIIRVMMTMDDDGSWTDISSVDSSSIDSSWASDDNNGTNDDGGNVNDGGDAKVAMVHNVVKKSSNDVKRRIDIRSYDASVWAVKNKGRNVTTSASWTNVYYSDKYRNHASYNVRGTAVSGSCVGVITSKINYAVDKVCKAAVNKSSNIDSNTICNSRAAIVAAVCSHNATWVCRHRSVAYGGGKKSCSSDGSCMGVCMSTTDVAYVVDAHMWGRACAHHKGGNMVDCKDTMGSTSMKHRSTVASGIGDGRMVIIKASVNGDVIDSGSIRSSVISNGGIIKNTSNGKDTVTTKKSRKRGKAKSISVHAGSKDAAKNGVCTTMKRICRHGISRWSRKINKSVGGGTTISTTIHGVDSSWAANGSTNGSGSKTYTTSKNGTNTSGSRASTGSNSNSHRSCGTSNAVVSDAAANRGMIDAGSSHDTNCVAKVTKVTVRTMKTITIKASYRDIIRRMAANSGIITKVAKRKDVGTDIKYDDDHWVIVCDADCVSISSGCNIVRVHDSTNGSSCSSD

>Cl34639.1

MTDNKSNNIIRVMMTMDDDGSWTDISSVDSSSIDSSWASDDNNGTNDDGGNVNDGGDAKVAGNGATSDRVSRVSDNVVKKSSNDVKRRIDIRSYDACVIKRMTMARYIGKHVAVWAVKNKGRNVTTSGVDNCTGHYRMASMYVSDGTDGGGRVRHKWTNVYYSDKYRNHASYNVRGTAVSGSCVGVITSKINYAVDKVCKARSASHNATWVCRHRSVAYGGGKKSCSSDGSCMGVCMSTTDVAYVVDAHMWGRACAHHKGGVAGRATRSSCCNITHSKTYVHYARGVASAICRSSHTGDDDYINMVDCKDTMGSTSMKHRSTVASGIGDGRMVIIKASVNGDVIDSGSIRSSVISNGGIIKNTSNGKDTKSISVHAGSKDAAKNGVCTTMKRICRHGISRWSRKINKVNRSTKKRVISVGGGTTISTTIRGVDSSWAANGSTNGSGSKTYTTSKNGTNTSGSRASTGSNSNSHRSCSHVKANAVVSDAAANRGMIDAGSSHDTNCVASKRTMKTITIKASYRDIIRRMAANSGIITKVAKRKDVGTDIKYDDDHWVIVCDADCVSISSGCNIVRVHDSTNGSSCSSD

>Cl60621.1

MAYSKETIIQYFHKTIRQAAKELNVGLSTLKYNCRDVGIQRWPYRKLKKIIKMLKKEKIQLENNPDLQLARTTKKLRQCNFKAKYQERKTMILVFSLSNQTTTNVYCCSIYLLPITQNMKKWMMLM

>Cl57857.1

MSHITKDRKITKKNATKNDCSNVTKDTINTVRDVIWSCKGKHVKTIDIDIANDGIYRRSIMAVDDTCNDNVVRVKRRWTCNITSHGYIVDSTMCVGVIVTIKYTDAYVHRIKTNKSTVGASNVNDKTRIKDICDTHKATWTVSTSVVTGRNINKTCSSSSSCIGKTCMSTNYVDNRYVHRACSHHKARGVVGRASHGSCCGDVTKDDCAIYRSIHDAYVVDMKIADNMNKHTSSGGDNTTIGVSTVSKSSAVDIIHTSTAVDTRTSAIDCKNADSRSTKRVCRDGITWHRKKKYAGIYTAAVRKRWSCSSNHHGSRWWTCDKHKDSKSKMSRANDSTMVTSAIIAACTSSKGIYAAGKRWRKRSAIMRTYNDSAIGRSNTIDKHASKDVGHTNKVTVKAIDHDTVKISSGKTVARIRSSRDKYRDDDDIIACDADRNMSATSGSNITKSVMVHDKKI

>Cl60370.1

MDDSSTITIGSNVTKDTINTVRDVIWSCKGKYVTIDIDIANDRNYRRSIMAVDDTCNDNVVRVKRRWTCNITSHGYIVDSTMYVGVIVTIKYTDAYVHRIKTNKSTVGASNVNDKTRIKDICDTHKATWTVSTSVVTGRNINKTCSSSSSCIGKTCMSTNYVDNRYVHRACSHHKARGVVGRASSHGSCCGDVTKDYSVHMARATDTSCAINRSIHDAYVVDMKIADNMNKHTSSGGDNTTIGVSTVSKSSAVDIIHTSTAVDTRTSAIDCKNADRDSGNDYYNVASINRISVGYGSVKKSVKSRRKRKSTIDTYRMGASRSGVSRSTKRVCRDGITWHRKKKYAGIYTAVVRKRWSCSSNHHGSRWWTCDKHKDVSKSKMRANDSTRVTSAIIACTSSKGIYAAGKRWRKRSAIMRTYNDSAIGRSNTIDMHASKDVGHTNNKVTVKATDHDTVKISSGKTVARIRSSRDKYRDDDDIIACDADRNMSATSGSNITKSVMVHDKKI

>Cl32787.1

MPFINFKSVQKFDETFKNQLISVIYTSQLLFYHTEIRYPHSKTMSFSRDVVSHYFHMPLHRAAKCFHVGSSLFKVHCRSVGIKSWPYRKLLSLEKLINYFQGKEGDSNTNENMQDIITKLQEEKGG

>Cl42819.1

MISDAVWKTIIVKKSCGTNATNSYGVVTIGYKISHASTSDINKTIHSRTVINDSGKMSCGRMTNVGSTGSHSVGTTDMTNVASTSKIIYKKRRSAGTSHRAAKIIHGSAHASAACAVWRSYDIHYRIHNSIITAKSSCNHTSNWAKDIHKKRKMDYNTDRNYCISNSCGRSGDDDMTNMDNRIRAAYDMKKIIYHSKTMSSRDVVSHYHMHRAAKCHVSSSKVCRSVGIKSWYRKSKINYGKGDSNTNNMDIITKIRAITNNTKKRRRKATYRKKKRINDSSIVDVVIYNNYAICHISYGSNYT

>Cl16046.1

MAYNKEKISQYFDMTIRQAEKELNVGLTALKYNCRDAGIQRWPYRKLKSIKVMINNFQAENGGAKPNDNKREIIRRLQEEKSQLEEKPNLQLAKNTKKMRQCYFKAKNKKRKTMGLLLSLSNQTPTEAIPLQSVSPTNDSNYEEMDDAYGSRCDKQ

>Cl75744.1

MYSKDKIGWASRAMDNSVSMDGARADNSSMNDTYAGWCTSSAADMASATAGYSSYNVKSGIGTVGNSINGGGVNSNDNGGSCKNVNGTGIIHRSSAKMKASKDSSGAGIAVWVIKGDTYISTCYDMAGYRVSRGKSAVKGHGGRVTSKVWTSNVAHYNKGYRIHASHIRGSIAIDDSYNSCCAVVTMKKSDSAMDHVCRASVNKSVARHKCSKTMAATITDVRAVCHAHRVATWICRYNKVGDSINVRVGGCNNNSTDKGICITDACYVSRMGVACSHHGGIAGKASNHDVKYHISYVHARKYGNAAVAIRRSTYTGDDDYIASMKGSTNNSSTMRICKSRTVSDASGVGGSDVGGDSGSRSGSKYMSSIGDVSGNAIDSTDIIGARDTAGGSRRSKKRSTAKNVSSVYSGSKDAAKAIGVCTTKRICRHGISRWSRKINKVNRSKKITVDSVGVGGKDTTGGVAANTVIDDAKSTSNNSGASRSSCNSNIVKDDCYMGIDNRSNSVTTSMGCSDSKMAADSAKTWTSKGCSIDGMKVDKSNRNSHSWSSIAAGDDMDTSMNDNDAIDDHNSTSSGMTSSNGSMNGSSSSSSSGKNKTTSGDGSKTVKATYKDTVRKDSTCGCYVAKRKITGTKYKDDWVVCSDWNCIDYGTRSVKVRDATAMGSSGSSNCCMGGS

>Cl19108.1

MELSKTVIAQYFHLPIAEASKELNVGLTVLKQFCRDMGFQRWPYRKLSSLDLLISTLQAENGGATIDDYTKVIITRLKEERRRVQENRLQPFSNHKTVETEKLQSRTQQK

>Cl52637.1

MAFSEEVIAQYFDKPITKDAKELNIELTVFKRQCRDLGIQCWPHRKLKIIQALIDRIEADNGGAQPDEYIQEILRNLKEEKKQIKEKPYLQLA

>Cl45182.1

MQLGTIRATLKRLSRSFGVSRWPYETDKSDSLNSIFKGSNEPHFTTNITHSPATFSKHGKHSSTSIRHQQVQTNLPDELAQPEITNRNSMTLLTIKATYKEDMVRFSFKLSDGFIKLEEEIATRFQLKLGNFSLKYKDIDGDMILIACDRSLSVSVGDFRLPEGRQTVIRLVVCLLAR

>Cl71826.1

MALFGKSDSVDSAYREKSSCSDPRSVLSTDQNKDLPRNKVSLEPPTVSSSGESRYLTRLWVSRNEEPLNELYKSIRAVLKQLVLRAQHVLVQFWSPLVVGKHQLLTTLDQPFGLGLPVEELYFYRKESERNLFLVCMDDEERDISPPARVFRRGLPEWTSDLTNYLPKHFPQQDCAIRCNLHGYLALPIFDSTTALCIGVLELLMSSKNTDYTFEVQQVHKALKLQNLTCPQTFDCATPQVLSERRQNVWDKICCILKSVCDIHNLPLAQTWAVFPPNSFVSHEKTLQKSCSSFDTKCIGKVCMSTTSLPFYVRDMGMWPFREACKMHHLDKSCSFVGKALLAHSSSYCEDITQLCEEEYPLVHNARMSGLTGCFTIFLHSIEGDNGDYVLEFFLPLNSKDSRHVLNLVQTLKQMIVVASGFELGEISPIQITGSPRDESCLSLSVETQSIHISSTTMAKTLAFGMDSTDSESVLANIVKTDSADDQSQCSSKENYTNDMSDNVNIVNSRENDNVASYSIVTNQNPSDTITDAGEKSKKRGRKRKIDSLTMEAVVQHVGKPINQAAESFGVSRSTLKRFCRENGILIWPKPCQSKKLGMLPNQNQIVYTVAQLMVKAILKVI

>Cl08782.1

MSATHKDNVIVYDNVRYNTVSAASHDDHDWRGCNAAATVYIYSSISMAGKSDSVDSAYRKSSCSDRSVSTDNKDRNKVSTVSSSGSRYTRWVSRNNYKSIRAVKVRAHVVHWSVVGKHTTDGGVYYRKSRNVCMDDRDIKWTSDTNYKHDCAIRCNHGYAIDSTTACIGVMSSKNTDYTVVHKAKNTCTDCATYYRSVNAGKMWDKICCIKSVCDIHNATWAVNSVSHKYRRVVVVTNASGKACYHMTWNVAACKMHHDKSCSVGKAAHGSSYCDITCYVHNARMSGTGCTIHSIGDNGDYVNSKDGRHVNVTKMIVVASGGISIITGSRDSCSSVTSIHISSTTMAKTAGMDSTDSSVANIVKTDSMTKANKRAKRGRKRKIDSTMAVVHVGKINAASGVSRSTKRCRNGIIWKYSMYVAMVKAIKGDMIKRISSGNVARDKGKTIIKYKDNDWRITCDDDHNASNTTIKVASK

>Cl70365.1

MGSCSHSNSSDGKTGGSVSGIDVVGINSKNISISVSGDSRYTRAVRNDDSSAMITSGITIVITHTMKHKGGDKINHKKIIDKIIVAKTRHVIVWSHAHVVGKHVTTMDGGVITTSYRKSHHNDVVDNDHSARVRRRWTDVANYSKKDCAIRHGHGYAVVDTSSCVGVITSSKYISYAYIVNTKTANITTAVDCTHRNKIHNIKCNTHATWAMSTAASHDIKKSCNSHTRCGKVCVSTDAYYVRDRWSRKACKHDKSCSVGRASHGSCCDVTKSYVHYAMNSSCAIHSISNNDYVHRYGRYIHKVTMKHSIIASGGDTIVIVMDTNSTIISSITTTNTKITVSSDSSIVADVAKTSYTHVVNSTDNNNGGTITSTIVVHDDVIRDDDNTTSNSDMIMITSTITDAGKSNRKRGRKRKIDSTMDAVKKHVKIAAAKSVVISRSTKRCRDNKMTSWKHNKNRHRKVTTSRSKNWTSNVVVNVMGRNWWSCGKHSHKSIYTKDHSTKNKNVDRKDSNYASNIKSNVSDTRKVTVKATKDDMIKIIISSRNVARRTKKSAGRKYKDDGDIACDADNGGYSTSNNTTIRIIADD

>Cl60901.1

MGSSSDGKYDSDTTMKVSGSRYTRCVGNSSSAIITSGRDDKITDKIRGATRNVIIWSAHVVGKHVTTMDGGITTSYRKSHNYVGDNDHSARVRRWTDVANYSSKKSAIRRGHGYAVVDTRSCVGVITSSKYISYAYVVRSKAHNAHRNMIHNVKSVCNTHRATWTTSTAASHDKKSCSSHTKCGKVCMSTAGYYVDNWSRKACKHDKSCGVGKASHGSCCDVTKSHYVHDAHISGITSCAIYHSVSNADYVKNIGRHVNVTKHIMASVGDKSVVVGSIASSVSNTGSNNVIGDDIDIVGTGNDNGTSNHDVSMIAINTVTGARSNRKRGRKSIHSTMAVKHVGKINAAKSGVRRSTKHCRDHISKHKTARVVDSKSRIGSSSVRNYAVVIVGVKRWNCSSKNHGSGRWWICGKHSYISKDSMYNRDHTDNTKKCKTNIRKIVARVASDVRKVTVKATKDIKTSSGMNVAWIKNKRRKYADDDDMIACDADHNGSISNNCIIKSIVDE

>Cl63618.1

MAFTREIIAQYYYMPIAKAAKKFNIGITIFKKRCRELGIQRWPYRKLTSIQLLIKTIQAENGGAKVDGNTQEIITRIQEKKKREEENLDLEFTKSTKRLRQFYFKSKYKEGKA

>Cl33172.1

MDNVTNSDSMDDYMDDGCWATDGSINNNTSASNDSVWKYSNTNNDNAGNSSKSHVKSITNSNASKRWWIAASSGSISVMRIYAIDNIKHYTVDKNVIVWTIKGKKYSTSRSNCSNYRNISTYIAGDAKSVGGRVKKVWTDVRKIYRVSHAAGVRGSVAVVYDNSCIGVVIMTTKSNYSSISVCKAADRSSDSNTKIKVNNGYHASICAKSACTIHNATWICIDNKGGCRHSNNIRCISTVDSASYVSDRKDACSHHIDGVVGKTTTMGCYSSDVTYKKTDYAHHASIDHAAVAIRRTTYAATVDVVDCKNTDGIINSIIIKVCGSRVTMGVNDGIVGSDTTMRVIDGGGSGSGSGSGSGVKRRAGAKKTITMRHAGSKDAAKNMGVCTTKRICRHGIRWSRKIKKVGHSRKIVMDSVHGGSGSISYSNKASDSKTNSTTKITDKTTDGATSGSSSSSGHSSGRHVCDDTGDGAHKRIKSDTGGTHKRTKSDTDIHTCASNDDDDDAKSTKCKKRIKGHWRVKVTGNIRRMKGWGYNVTAKRSNDISGYRYDDDSWVTCDADVCIDVYRSKGGTIRTRRVGSSGSNAL

>Cl23593.1

MALKREVITQYSHMPIAQAVKELNVGLTAFERHCRDVGIWYWANRKLKSTKTLIKNFESLSPNNHSDYEEMNDGYVSDYDDQE

>Cl54961.1

MDDASTKASAMDSIKSGIANDDTSYSSSNDSSSSYNSYNSRYDDKTDVKGASHVDYTNTDNTHKKTAIYKATRDVDVIWVRKRGCVTTDINSNNKGGYRDISKSNATDNSAGSVKKMCTSGGKDANNDNCGDVVGSGTCGVIIVTISNVNYRHNNIRKAADRVSIHKDYNGSYVAIRVRSVCDTHGATWSCAGRGGCSAACISVMSCASYVDNVGCSHGGIVGAGTNCATDITRSTTYSDYAKRSGAVAIRRSTYTGTDIRDCRNNDKMSSISYVINVSKSHVINGDGVYSSVKTGSSWISYMMAMGSAIMASKKYDYRSASGKNIDMKRKGKKSGIRKSDKKRSRAKNISVHSGSKDAAKSIGVCTTKKICRGHGIMRWSRKIKKVGHSGKIIDSVGAKGTIASANSSSTKTNDSANTSKTTYDSSSCGSRSSGSSTCCSKVNTYAKSGNTAGGKSVKDVRVKVIYGKKIRIRMSRNWDGRIMVRNIDDMKSITKYDDDSWITCDGDCMDINTSKKCIINSIHSRSYTGNGSS

>Cl25462.1

MSFSRDVISNYFHMPLHQAAKCFNVGSSLFKVHCRSVGIKSWPSRKLLSLEKLINYFQGKDGGSNTNENMQEIITKLQEKKRRIKEDPELSIINNTKKLRRRFFKATYRKKKHINLELSLAQPIVLDVEPLQVIYPNNHESYEDIDFS

>Cl66364.1

MAFTREVIAEYFNKPINVAAKELNVGLTILKRQCRDLGIQRWPHRKLNSIKALIDNIEVENGGAQVNEYIQDILKSLKEEKKQIKENPHLQLALDTKRWRQINFKAQYEKRKAMALILSLINKQTTNVDTGCNLVS

>Cl06132.1

MAFTREVIAEYFNKPINVAAKELNVGLTILKRQCRDLGIQRWPHRKLNSIKALIDNIEVENGGAQVNEYIQDILKSLKEEKKQIKENPHLQLALDTKRWRQINFKAQYEKRKAMALILSLINKTATNVDTQVQSQSLNNNATTDETEDGYCKHYAE

>Cl69708.1

MAFTREVIAEYFNKPINVAAKELNVGLTILKRQCRDLGIQRWPHRKLNSIKALIDSIEVENGGAQVNEYIQDILKSLKEEKKQIKENPHLQLALDTKRWRQINFKAQYEKRKAMALILSLINKQRTNVDTRVQSRLLTITQLSMKSRRLL

>Cl20227.1

MAFSREVIAQYFDKPITTTAKELNIGLTVLKRQCRDLGIKRWPHLKLKSIQALIESIEAGNGGAQADEYMQDILQTLKEEKKQIKENPDLQLAPDTKRWRQVNFKAQYKKEKLWH

>Cl25744.1

MELSKTVIAQYFHLPIAEASKELNVGLTVLKQFCRDMGFQHWPYRENGGATIDDYTKVIITRLKEERRRVQENLDFNLSPITKQLRQRNFKADTTKEENGGAIVDDYKVIITRLKEERRRVQENPNYKLSPTTKKLRRRNFKARYKRLKHELRPTTTIEATPLRVIYPSNISDSKEMDGDYDGDNETKD

>Cl51164.1

MDLLTNDQDLIDMDYWLHYFHLDDQLPFGDDTLFTWLDKQSKDNISALEFSYFDPIFVEDLSLWDCFNSSLPLLCSKDDNTTQSHFSSGEAYSSSTTTITTSTTTAIVDHGDRSGRTRVRPLELEEIRKHFEMPIAMAAKELNVGLTILKKRCRELNIKRWPHRKLMSLKCLIRNVKELGLKEEVEMLEGQKRMMEKQPEMEFNLRTKKLRQACFKATYKNKNRRFNSPN

>Cl42240.1

MELSRDVIAQYFHMHIAQASKKINVVLTLVKRHFRAVGIQRWHYRKIISLQALINHFRATNEPTPLQTIFPTKYSDYEEMDDSYSSDCDEQV

>Cl69746.1

MDSHQKPDGSSFSSDSSSGSDGSDYGSGSDSGMVNINDTPNKNISLELQTLSPSTASTDPVRLLTVTSGFERRGENIHPHLKIISDKIIAVMKSLTFREPCVLVQFWSPNVAEKHQIIMTTDQPFGLGVVEGPFCSYRKDTKHNLYIVDNDHEEDHNPPARVYRRGLPEWTPDVANYSPKHFPEQEHAIRCNVHGYLALPVFDLFTKSCVGVLEILTSSKFLSYADEIRQIQSELKKTFLTTTQASDCPTIIVDNEHRQKELEKISRILKLVCDTHRLPLAQTWAMSPLTTFASHEQILKKSCNSFHTRCFGKDCMSTNTLPFYVENSGLDSFRKACEDQHLDRSCSFVGKALLSRGSCFCDDITKLSVEEYPLAQYAHSRGLTSCLAIFFHSTQSNEDYVLEFFLPLHRENGRYEQQLVQTLKHGIGIVSEFELGVSTQVDEPFVDLSSKKESATIQTSSHMLDRIPQNTAIDFDMEELFLLFLEWLQANTNSTHVRNKLSPPKKFPNNGDSITYRKRKLDSLTVKAVKKHGGKPIDEAAKSLGVSRFALKRFCRNHKLYSRPSTKHNTKTNNLNMPQRSQSKPDLTPKNKSKVDLKLPESNFVDASLKQIMASVVPDVRMVRVKAYFKNDMIKFQIPVSSGMFELRNEVELRIKLGKMVRLKYRDEDGDMIHLISDHDVQHRLWSLANYNSTLYLYIETDD

>Cl15835.1

MSNSMTIWSNHVNSAKDDVSINHGDIGCNTYDHMDHYSGAYMDIISNDYSSDIVSNCTDHMTYTDDMSVYGINNGSNDMSIVNSTGDNGMIAYTDTNAGNMNSMRNHNMKIDNAKSRDAANSRNIRKKGRGDHNGSSYTSKSRTISYYMITAAKNVGTKKRCRGIRRWHRKMSTINNVGKSSGAADKMRAVMIRKKMIDRNTKRRACKANYKKRRTMGVVASSANSTSINGSSSSCSSTCSINVGYVVDDDCGDDVMKSMSDCASSNNSIF

>Cl67136.1

MAASSKAVARNICISGKVTKVSHGTNGVWCIVDKDRWSVGDIDRTVTISRNHKKTITANDTIISSSIKCTSDSAGGKDKNCKNRHRYGKKKKRAATKVIASGDSKYDVIIASKSKVGTVKKKCRGIRWHRKIKSDGSNVARDTGAAKAVAMRMISKTIKKMDRTKKIRQDIFKKRHRAKALEGQGQTLALF

>Cl23223.1

MDDGVSNNMGNADSMMDYMDVDGCWATDGSYINNNNINNIDTIWDNNIDRVDVRAKSSIKNSVSSTDGISKSVSNSGNSNSSTSRRWWIKYKTGAKNTIIWKTKVSTSDHVRIAHNIIDTYKVIKVIKIITRRVYMGKVWTDVRKDDYRVTYAYDVRGSVAVIDNSSCGVIVVMTTKNNYTIGVCKAAVDRSSSSSSKSMTDGYRAAIMIKSACKTHNATWICTGKKGGCRHSDTNIHCISTVDACYVHDRYKDACSHHKGGVVGRAKTNCYDVKMTKTYSHHARINCGVVAIRRSTTANVDYVVDCTDKSNSSVIINVCRSRIVTDKAGRVIVKVITDITMDIKTNSGSGGSVTDNVKRRRRGGSKTKTITMRYAGSKDAAKHGVCTTKRICRHGIRWSRKIKKVGHSKIVMDSVHGASGSISYSNNASTDKTNKVDSKIAVTKSVSSSSTSSSGSSSSGTHHSTSAASTSDAVHVSYSDDKRKVRSHSHKSTTTKNGNNARDTVRVKITYGKVRRKDWGYNVIAKRSININHKYDDDSWVTCDADLEECIEVYRSCNSGTIKLALFEPHQVGGSLGSNVAW

>Cl20265.1

MSIRDNRSKVIDSNSRYSCHDSDGSTSRGRRCSWVYGNVDIKSSKDDNIIDSIIKDKVTAASGRRRVVWSITRKRCTTDGGAVDGYYRSRMVVDGHRDIGGRVYRKWSDINMSTRNVKDWAASYNIHGYINVDSGCCVGVITSSNYVDYAVVSRAKKSNVDINVGDRKHDICAKTICDVDIATWGSGYSSVVANSGNRTCSSYNRKCIGKVCMSTYGYYARNKMWGVACTHDKSKGIVGRSRSHGSCYCDVTKGDYGYYARVNGITSCAVYSVRDVYVIVHKANGADSKTVKVIKASSVKGIMSSVIGGNWNSSITDKGVDGNVDNTVTISKDSVVAVNVHIHDYNSVAAAAAGTSSVCVDSDINKGKTRTNHNSSNKGRKRKGTDGSISDISKHYGKTMDAAVIHVSRSTKRICRSGVRWYKNGDKSDSIKATYKNTVSSMSDGVKITSKKGSSKYDRDGDMILIACDSDLRESVDDFKLPDGQTVIKLGLATC

>Cl64321.1

MKVSDAVGNSSKSKTKSSNSDVDSMKSRYCIYMNSNATDVDADDRRYTSVGTRDIDGSASSNMMRNIIDHIHKRIASARNIRRNGVVWSVVRNCWTTWDWYCDVHAGNNMYGYIDVSSGDSCKNRSNIDSRCVADRRRHHIKVKAVCDIHNATWASGHTSVAKGHTCSSNKSCIGRACMSTDDYVRDSMWDHKACRTHGKSGVVGTSSSCGTRYRDVTDDDYAARMSGITSCVAIYITSYDDYVITCNANAKTKTVRKNTSCVDIMAKTKNIYSSNSVSVGSSVYKGIADDTNTGTRMKGKRSKSSICIKHGKTIDAAAINVSRSTKRICRNGIRWYRYSITSTIGVMDSNIDNRNDTNKSDINIKKRTDISVGSINHSADNVKDMVMSNSDNSSNSGSVGTSGVVRYRGKGIRKRKRSGRVIGKIKHGKMIDAASIMVNSMSMVGHSTNTNDGRANINTATYIVNTIKASYKNTVISDGVKIATKGGSKIKYDSDGMIIACDSDISVGDSKQPVDPTVLTLLVFARC

>Cl46011.1

MKSGTKVDAKSRYRISKADAGRDNDGRYTRWVGTHDDHSDSSKMIDNTIDMITISASARRGRHVVWSATVRNRCTTWDAGVVDDGYSYRMDSRTVVDGHRGARVYSKWSDITTRSVDGASYNIYGYVVDSRCVGVVISSNYVDAVGVSRAKKKRKRSSSISIKKHGKSIDAAAINVSRSTKRICRNGIGWRDNGSVGVTSDHYRAVKVNVDSRARAAVSRKIRTRGINYGNGDKSWRDTISRSDGMVVAARGGSKKYVDDDDMIIACDTDMAGDRSDNTVIKVVVHQSPDA

>Cl49345.1

MVVDAGHRIGGRVYRKWSNAHTTRSVDSSASHNTHGYIVSGCCVGVITSSHYVDYAVISRAKVNKCNVYDTYTHVGDRRNDIIAKAICNVHDIATWASGYGGAVAISGSTCSSNSDCIGKVCMSTYGYVRASMWRYHDCRRHDKSGVVGKSSSCGCCDVTKGDYAYAHMNGITSCAIYKSIAGDAYVIVDYTNSDSMKTVKIHNASSKGIVSIVIGGDNWNISSITITDVGKSNDRDDVDDVRSKDSVVGYHMDDNNSSDSAADGTNNVVCDAGADSSDIKKGKMKRKRKRSKSIRISKHYGKTINAAKNHVSRSTKRICRTHGIRWYKSGSDKSDSKSNTDVVAVHAYGATIVASSVTTNVSDHTNHTHGKSSTVHTNTAGSATSTKINAAAANVMKNVTIKATYKDNTVRTISDGVNNVATRGVGSRKYDADGDMIIACDSDMGSVGTRDDAVIRVVHHSPDA

>Cl06657.1

MKHVSNVNSSKSGSSNSDVASMKSRYCIYMNSNATKDVDDADRRYTSWVGTRDIDSSSNMMVNIDHMIKKITSAKGRRKGVVWSVAVKKRWTTWDGVCVADYSYRKSRAIVVDGDNVGVGRVYSRKSWSTCKIYGYINVSGNSCVGVIITSSNYVDYAVHVSKAKKNKSNVDHSYISDKRHDGIKAKMVCDSHDATWASYGGVAKSGHTCSSNKSCIGRCMSIDDCVRDSMWHVACRTHMGKSGVVGRSSSRRSWCRDVTDDYVARMSGNSCAIYKSSIYVIARSAAADRMKTVKINNSCMDTMAVIGGAKWNSITSKKMNSNSVAAGKSSVIHSKGVGDINGKKRRKRKRSRSIIRKHNKTMDAAANHVSRSTKRICRNGIRWYKIDKTDSVIKDTDKTHDVSTNGKDSHRKKSSTIIANNVYVTIKATYNNNVKSDGKKIARKHGSSRKYDDNDISITSDDMVTTTKKAVIVVVATSNA

>Cl04888.1

MRSNMSSNTHTTSIYIAKRVSAKRRRKKMSVTMDIHIGKIGASKRGVSRSTTRICRKNISWKRRNMKYKHSIHVVVIKVKRWSCSSNRHGARWWICGKHSAYTIAHGSSMIASSSKSDDITRIARSDMASKKKAKVSDIRTVTVKVTYKDDMIKSSGKVARRFMFESTRLHLKYWDEDGDLILVACDADLSILIMPYSATTVGNNTIKLIVEIADD

>Cl25480.1

MKSRYCIYMNSNATDVDADDRRYTSWSIYCDHVSTGSNMMRNIIDHIHKRIASARNIRRNGVVWSVVRNRWTTWDGIGVADAGYAYRKSRAIVVDGDRGGRVYSKWSDVHAGNNMYGYIDVSSGDSCVGVIITSSNYVDYAVVSKAKKNRSNIDSRCVADRRRHHIKVKAVCDIHNATWASGHTSVAKGHTCSSNKSCIGRACMSTDDYVRDSMWDHKACRTHGKSGVVGTSSSCGTRYRDVTDDDYAARMSGITSCVAIYITSYDDYVITCNANAKTKTVRKNTSCVDIMAKTKNIYSSNSVSVGSSRDRVRVWRKSKHGKTIDAAAINVSRSTKRICRNGIRWYRYSITSTIGVMDSNIDNRNDTNKSDINIKKRTDISVGSINHSADNVKDMVMSNSDNSSNSGSVGTSGVVRYRGKGIRKRKRSGRVIGKIKHGKMIDAAAIHNDSMSMVGHSTNTNDGRANINTATYIVNTIKASYKNTVISDGVKIATKGGSKIKYDSDGEMILIACDSDLIESVGDSKQPVDPTVLTLLVFARC

>Cl58703.1

MKSGTKVDAKSRYRISKADAGRDNDGRYTRWVGTHDDHSDSSKMIDNTIDMITISASARRGRHVVWSATVRNRCTTWDAGVVDDGYSYRMDSRTVVDGHRGARVYSKWSDITTRSVDGASYNIYGYVVDSRCVGVRMSNIKRGSNSGAVRTSHDIGYSKVINDNTGTIKKKKRKRSSSISIKKHGKSIDAAAINVSRSTKRICRNGIGWRDNGSVGVTSDHYRAVKVNVDSRARAAVSRKIRTRGINYGNGDKKGTTNITTHGKHCTADDNWTIATYRNTISRSDGMVVAARGGSKKYVDDDDMIIACDTDLMELAGDFRQSDNTVIKLLVLPVVHQSPDA

>Cl30787.1

MAAYSTNTCTSKGAWKKISDGDSRRANHYICVTRKIKSTIRNYGHSVWAATKIGKTVTTTGGSGTNYRGCDHKYVHKRIAVGGRVNSTTNVHNINDRCVDATITRIWSSAVVIVDACVGVDVMDTTHSYDSIIGKAGCSIKTDCKTCVSNSKIRIRRTSSSYYRVYGSSVVAAHRKKGTRNTCRKAGIWGSTNTRASSADTRVSTVSDTSSVGTVDDSVSSRDGISTIASSTINTVSTSNDDNSCWDGGVYGMTNASATTINIASDTSTANHDGIYGITKAVDHYVMDSDTIDALMI

>Cl36831.1

MDCSHSVDSDMADITAVSSRYTRVGNSRSTVTRGRTYNIDRNKIIDKIRGAKITRYVVWSRVVGNHTSVDGGVDRYRKDSVRNSVVDKGDDDTSIARVRGWTSDVTNYKDCAIRCNNGYAVDSDTRSCVGVITSSNCSSAYVRAKTTNTTTADSNINVVHYRNRISIKAVCTHATWAVSSSASHIDKTCNSDTRCGKTCMSTASYVDGWSRKACRHVDKACAVGRASCGSCCGNVTKSYVHNAMSGASCAIHSISNDDYVSNRDDGKHAHNVTKNMIAYGGDTSSIVVGTASSCITTTKTNTTASSDSSVGDVAKTNSTDVVNSKNNGGTITSTISSDDVRHNVNVACTKDNGNVMYDHDVSMHMTYVGKSDSKGSKRKYSTMAAKHGKTDAAKSGGKSSYRSSNVHGSSVVRIKRWSCSSKHHSGKWWCGKHSYKDSVYVARVSKRWRNRKSSMHAIHNRDHTVSINKYKNIIKYCHVNRKASSVYASKHIVASVTSDKKVTVKATYKDDMIKTSGNVARINKSKRIKYRDDDDIACDADHSRSSTSNNSVIKLIIVLADA

>Cl17046.1

MIDDKSRYSCISNDSRWVTSSKMMVDNSIDMIKKITSVSARRHVVWSVTVNRCMTNGGVVHDGIYHRKSDTDVDGSARVYRKKSWSDITSTRNKDSACYNIHGYIMISNSGCCVGVVTSSSNYVDYAVISRAKNKSNVDIYVSSIHTISISARKVSNRRCAIGIAKTVCDTHKATWSSGKNSVTSSGTCSSKKSCIGKICMSTTNYVRDMSMWGHACIRHKSCGVVGRSTCGSWCKDVTDDDYVARMSRTSCAIYIKSIASYVITGKNADIVKTKIKNVGITSTIDVSDIRMSVNINSNSGVVGACAVVYKGVTYRKRKSSSSISIKKHGKIGAAANNVSRSTKRNCKAHGIRWYKMKRTDISVGISCTMKVIDDRVRMSTSWMSTSDSSCNSGAVGTSHGVVYWKKRTTRRRKRSRVITINHTGKVDDATAINDSISYWHGDRGTRNTHDRVAHGKNNSTHTNMDVAKMSINTAANTVKYMKIATRSGSKKYDKGDMIVACDTDLEASVGDSRQPVDPTVIKLLVFARCPSEP

>Cl03294.1

MACSTMSSGWTDTRSVYWNKVSGSSSNHYIVTNKVKKIISIARNVSVNVCWAKKIGKSIVTTVCGSGVNKVYRKGCKHKYVHGKAVGAGRVNCIHTDMHNYKDRCDAISRIWGSIAVVINGCVVVDTMDSYDNIIAVCRNAGSSMKTDCRRTCINTNINSRKRMRRTRSHTYYRVHGSSAYAAMGIKKGTRNVCRNVGIWGTKNTRASSAIRVSTISIASRATTVKASNHVNTVTSNANDSCWDGGVYGTIITNTYATINIVDTSYTADSHDGIKYGNTMAIDDHHWGMDSDYIDAMMTWAA

>Cl48008.1

MVDMWKKNTTYKRDSDRYTTVSKNGSGSSYSVITCGGSKNIVHHTVMKIRAAKARDKHVVWWRDVGKHKTIVDGVGVADRCSYRRHSRNAIVDKDDGDIGARVRRGWTSDTNYKDCAIRCNHGYVAVDSNTSCVGVTSSKYMSAVVYKAKTNTSVHDSTKIYSRRDKIGIKAVCDIHNATWAVSSSSVSRNKVIKSCDSDIKCVGKVCMSTAAYVKDSVWSRACKNKHDKYHGVGRATCGSSYCDVTSYVHNARMSGMSCTVIHSVGDVDYVDSKDSRHADVTKKVVASGVGDISRVGSSIKSSITTANTAGMDSSNTANAVKTDTANVTHYSSKNDKCNITNSVAWNDVRRSINVAGSNKNDNATSTIVIKKTSDTITDAKSSGKRGRKRKIDIRMAVKHVGKIGAASVGVSRSTKRCRNGISWMRKKTSYVTDKSSGSKRRSSSCDCVVVVIKKRWSCTSKHDSGKWWRCGKHKYSISDTVYVVKTVKATNGDTIKRVISSGNVARGLWGKRFNLKYKDEENDLLLLTCEDALHTLPELLVSNTTIRLIVELACD

>Cl86177.1

MACSTMNCTDTTSVSDIVSGSYIWSGRNHYIRVTSGKKIISVIKNGNVVYWGAKRTGKAVTTTCYGYGINIYRKACHKYGYDTKAVAVGARVNSAHTNHNDYKDCCDAISRIWGSAVVIVDGCVGVDVMDTSMYSYDHIAVCRHGSSIRTDRRTCIKINTKKRMRRTKSHTSYVCVGSSAVAAMGTRGTRNTCRRAGWGISKNTRRASSADTRVNASDTSSIGTVDDISNCSDVISWSRKAIVASASATCINKVSTGHIADDTSYWDGGVGSVITTTTVACDTSYIAMDMYYSYDTISDADYTVAMKDMNYGYTTIMEDHNIQCLMDQLRFLLEDAPAYGQYGII

>Cl16452.1

MSVIKNGNVVYWGAKRIGKTVTTTCYGYGINIYRKACHKYGYDSKAVAVGARVNSAHTNHNDYKDCCDAISRIWGSAMVIVDGCVGVDVMDTSMYYYDDIAVCRHGSSIRTDRRTCIKINSKKRMRRTKSHTSYVCVGSSAIAAMGSRGTRNTCRRAGWGISKNTRRASSADTRVNASDTSSIGTVDDISSCSDVISWSRKAIVASASATCINKVSTGTGRGSVITTMTVACDTSYIAVDMYYSYDTIDADYTVAMKDMDYQGYKTKVMEDHNQQCLMDQLRFLLEDAPAYRQYGII

>Cl30657.1

MSTGDLPFYIRDMSRWEYHVASTERRLEKSRGVVGRSLLTCGSWFCKDTIQLATEYVVELFLPTHSTNKADLQSLVKTMKQQNVFHLGIMSSIQEIKVITHRKRRRSESSISLEEIKKHFGKPIDEAAAILHDDDMILIACDTDLMELAGDFRQSDNTVIKLLVLPVVHQSPDA

>Cl12968.1

MVKVSDAVGNSSKSRSSNSDVDSMKSRYCIYMSSNATDVDDADRRYTSWVGTRDIDGSARKNHICTNIRRGNGVVWSVVRNCWTTWDGIGVADGYAYRKSRAIVVDGDRGGRVYSKWSDVHAGNNMYGYIDVSSGDSCVGVIITSSNYIDYAVVSKAKKNRSNIDSSCVMAKGHTCSSNKSCIGRACMSTDYVRDSMWDHKACRTHGKSGVVGTSSSCGTRGDVTDDDYSISVMMMNMNIMAKTKNIYSSNSVSVGSSVIWRKGRSTKRICRNGIRWYSWGINHSADNVKDMVMSNSDNSSNSGSVGTSGVVRYGRDTTKKRKRSGRVIGKIKHGKMIDAAAIHNDSMSMVGHSTNTNDGRANINTATYIVNTIKASYKNMVISDGVKDIATKGGSKIKYDSDGMIIACDSALIESVGDSKQPVDPTVLRLLVLLVVNHNLAPRSYLA

>Cl86366.1

MAAYSTNTCTSKGAWKKISDGDSSRSNHYICVTRKIKSTIRNYGHNCTTTGGSGTNYRGCDHKYVHKRIAVGGRRCVDATITRIWSSAVVIVDACVGVDVMDTTHSYDSIIGKVCAAGCSIKTDCKTCVSNSKIRIRRTSSSYYRVYGSSVVAAHRKKGTRNTCRKAGIWGSTNTRASSADTRVSTVSTSSVGTVDDSVSSRDGISTIASSTINTVTSNDDNSCWDGGVYGMTNASATTINIASDTSTANHDGIYGITKAVEDFDHYMFMDSLDTIFEDAPLMI

>Cl39566.1

MNNNNINSTVNRSNKGSVDGGSRYTCSGNNINNNSNNRKRYVSRVYGNRVDDNSSADNIINIIKKITTAACRDSHVVWSVIVRKRCTTDGGVVDAYYRSRMVVDAGHRIGGRVYRKWSNAHTTRSVDSAASHNTHGYIVSGCCVGVITSSHYVDYAVISRAKVNKCNVYDTYTHVGDRRNDIIAKAICNVHDIATWASGYGGAVAISGNTCSSNSDCIGKVCMSTYGYVRASMWRYHDCRRHDKSGVVGKSSSCGCCDVTKGDYAYAHMNGITSCAIYKSIGDAYVIVDNTNADSMKTVKIHNASSKGIVSIVIGGDNWNISSITITDVGKSNDRDDVRSKDSVVAYHIDDNNSVSDSAVDGTNNVVCDAAADSSDIKKGKMKRKRKRSKSIRISKHYGKTINAAKNHVSRSTKRICRTHGIRWYKSGCDKSDSKSNTDVVTVHAGATIVASSVTTNVSDHTNHTHGKRSSTVHTNTAGSTTSTKINTAASNVMKITIKATYKDNTVRTISDGVKNVATRKSGSRKYDADGDMIVIACDSDMGTVDTRDDAAMSSKKNVRVNKKKTSDSVIHYGDNSFIRLRNEMLHVKYGIHLLAFSLCVDDIQKVYT

>Cl75074.1

MENEPSNSVAAGKSQSVIHSLEKGVGELDINPGKKRRKRKRSTESSIRFDEVKPHFGKPMDEAAANLHVSRSTLKRICRNLGIARWPYKIPDKTDSIIKLDQADKTHDLVSLTENGKDSPLLSIQSFGWIGKLKELIAERFQLKHGSLRLKYLDEDEDLISITSDLRLKAIGCSSLEYVDEDNDLIWIISDVDLMVFNNTSKSRHRNRYRIIILMESVFTESQKLL

>Cl61688.1

MEQRERKYDVSSTLLLLQQQQTTNQSRNLAISSSSALSYRHELPEESHMSSNTHTTTTGGIAEEKRVSAKRRRKPKMESVTMDDIKQNIGKPIGEASKRLGVSRSTLKRICRKLNIPGWPKPRRNMKNKHLSIHVVVLLLVKVSDGAVQVIGMAMKQGGGFVASIALSISND

>Cl61441.1

MGVLALSYEGEVGFFWVKGLIHPGFEHRDHNVYPHGLKIPDKIKLALKLSHFREARVLVQFWSPHITGDHKVLTTRDQPFGVGVIDEVLYSYRKDSECYRCVLDNDHKEEDHHPTARVFRHGLPEWTTNLTNYLPKHFPQQEYALQCNLHGYLALPVFNSSTPRECLGVLELLTSSKYRSYAYEVRQLYIALKAAGLTTTQAFDRPTFNLYEIHRLPLAQTWAISPPSSVASHEQILKKSCNSFDSRCLGKTCLSTAALPFHVQDLGVWPFRKACKELHLDKSCSFVGKVLSSRGSCFCEDVTKLSEEEYPLVHYAHLNGITSCFAIYLHSLESKADYVLEFFLPLNIEDSRHVQNLVQTLKQHIEMASVFELGDKSPIQVVGPSREARSLFLDTDPQSAIISSTITAMTKIPERVSSDSESLYFLDNNGGAITSSAIVIQDDVIRDDIDVVGAGNDENATSNHDVSMQIASNTVTGSRETSNGLKRGRK

RKIDSLTMEAVNKQVGKPIDEAAKILGVSRSTLKRFCRDHDMTSWPLPKHNKKTARVTDSKLSQKSRSIQPLQRSSSVRFGFFTAVILLVRVKRWSCSSKNHGFSGKWWSCGKHSYLPFKLDSMYQVARLILSLKRWKYKNSTKHVLQNRDHPTDSTEKPKFKANIINPGSSPVYASPKQIVARVVSDIKKVTVKATFKEDMLKFQFPTSSGLSELENEVAQRIKFKNKRVRLKYRDEDEDMILLACDADLHHLLGFSTSNNCIIKLSIQLVDE

>Cl29674.1

MACKIVTKSYVYTITTINKHHKASTCRKKYRGDNIGRRSTSCSSYRNSVSMGCSVDSVDRKSSSGSRTVYTDNRDWNKVSAVSGRYTWVSRNNYRAIINTSIDSATKVNCKKTWDRISIKTVCDVHNATWAVSTSVSYDKNIRKSCSSDTKCGKACMSTTAHVRDMGMWSRACKMKHDRSRSVGRTMAHGSSYCDVTCYVHNARMSGTSCTIHSIGDNDDYVNSKDSIHVNVTKIIVVVSGGISIITSRGTCHSVSISIISSTTTAKSAGMDITDSSVANVVKTDSADDSCSSKNYTNGMSNVNIVNSRNNNAASYSTVTNKSSDTITDAGKHIKRGRKRKIDSTMAVHVGKISAASGVSRSTKRCKGISWKSCSKKTSHATVAMVKATKGDMIKRISSGNVARDKGKRIIKYKNNDRRITCDDDLHNLPEFLASNTTIKLLVELPWN

>Cl62000.1

MAMKDMRNKSDAVVAGDSRYTRYVSGKGRSRSSSSAISGRADNTDRDNIRAAKTRNVVWSRVVGKHHTTVDYGVGAIDDRYMYRKDSVCNYVVDKDDGARVSRGWTSDTNYRKHCAIHCDHGYAVATTGCVGVIASKYTAAYVRAKSVNTTKHADSTNGRGKDSIKVVCTHRATWAMSSNGWRRSMSRTTGVMWCGRASHGSSCDVTKSYGSYARMSGTSCAIHSVKSNDGYVSNIDGRYVNVTKNTKMVSGIVGDKSTVVGTATSYDTDSTIIASTTTTKNKAIVSSDSSVADKARTDTHSGKSKMITNNDGGTIKSSIVVDDVKRDNINAVDARNNDDASDNHISTRITDVGTSNRVKRGRKRKIDNTMAVRHGKIDAAKSHGKSKVYYSRSTKRCRDNNMSSWKHNKKTAHVTDSKASSSSNIRSAVVVRVKRWSCSSKRHGSGKWWVCGKHSYISKSDSIYVAMVYNRWKNKSTKHAIKRDAVRSTKSHKSDVDKSSVYASKIVASAISDRKVTVKAAKDDIIKISSGIKVRIKSKRCKYMDDDDIACDADDYSASNNATTIKLIITLVDE

>Cl16257.1

MDSRRSSADSARKSSCSGSVSKDITDWNKTSKKTISDSRYIWVSNYRYYVSSSKSIITCIKDVVHKIKIRAAKNRHVVWSRVVGKHTATHGGVIDGTYRKNSNNVCVVKDDDSSARVRRGWTSDANYIKDRCAIRCNHGYAVDSTTGCVGVIMSSTNTTYAAVHKAKTVNTSIDSATVHNWRRDKVMGIKTVCDIYNATWAVSSTSVSHDKIIKSCSSDTRCIGKVCSTNAYVRDRVWRACKHDKRSVARAARGSCYCDVTSYVHNARMSGKSCTIHSNGNDDYIYSNIDGSYVTKIIVDASGGDISIIGSIISSTTTANTMAAGMNSSDSKSVANKIDNKGDISNNYNVASKTNDNGASHIVIKNKTTDTIVKRRRKRKIDSKMTVKHVGRIDAAKSGSKSSYVNSIISHGTIIVACSSVSRSTKRCRVNGSSWKSKKITNRHKITVLKPM

>Cl27512.1

MDSRRSSADSAKRKSSCSGSVSKDITDWNKTSKKTISDSRYIWVSNYRYSVSSSIITCIKDVVHKIKIRAAKNRHVVWSARVVGKHTTTHGGVIDGTYRKNSNNVCVVKDDDSSARVRRGWTSDANYIKDRCAIRCNHGYAVDSTTGCVGVMSSTNTTYAAVHKAKTVNTSVDSATVHNWRSDKIMGIKTVCDIYNATWAISSTSVSHDKIIKSCSSDTRCIGKVCSTNAYVRDRVWRACKHDKHSVARAARGSCYCDVTSYVHNARMSGKSCTIHSNGNDDYIYSNIDGSYVTKNIVASGGDISIIGSIISSTTTANTMAAGMNSSDSKSVANVKIDNKGDISNNYDVASKTNDNGASHIVIKKKTTDTIMKTGRKRKIDSKMAVKHVGRIDAAKSGSKSSYVNSIISHGTIIVACSSVSRSTKRCRVNGSSWKSKKTTNTNSKSSSSCKHIGVVVSVVIRVKRWSCSSKRHGSGKWWICGHIYIYYIYAAMIVKATKDDMIKRISSGRSNVARDKGKKRVKYKDNVITRDDDHDDSNTNIKIDACD

>Cl72809.1

MKSIDDVDKDASSGSNVNANSKYCCSTSASRDNDNGSRRYTVGSRVDDSSKNKNINSRHNTSYNKNNNISSRNTSVSMVVMSIWGNVKDCSKDNSIDRVKDKIRHASASRSHVVWSANDKRCTTDGGVVDGYYRSRRMVVNGDHKGGRVYRKWSDVTTIDDVSYNIYGYIDVDNGSCVGVIVSSSSYVDYAVVSRAKHNKSMADRYVGDRRHDIYSMKTVCDKRAMWASGYSSIVVNSGNSSCSSNKNCIGKVCMSTSDYVRDSMWGRACRIHDKSGIVGKSTRGCCKDVTKGKYMASYARGYITSCAIYKSISDIYVIVHDNADSGTIKIKNVSSVKGISSITTKVSRNDVSKDKSVAYVHMKDDNIMDNAAAGTSSIVCNAGIDVDIKKGKTTKIHHDKSNTKRKKKKTRSISVISKHYGKMAAVNHVSRSTKRICRSDIRWYKNDKSNYVKSNTDAVIRASGTIVGTSTSGTTNIYVTTHGKKGSIVVSHDKIVGSAKTTIRHIVNTATNTMIKATYKDIVKMSDGVKVATRSGSSKYDADGDMILLTCDLDLIELVADSRQPDNQTVIRLLVSHTAHQP

>Cl05335.1

MHDKSCSVGKAAHGSSYCDITCYVHNARMSGTGCTIHSIGDNGDYVNSKDSRHVNVTKMIVVASGGISIKITGSRDSCSTTMAKTAGMDSTDSSVANVVKTDSADDSCSSKNYTNDMSDNVNVNSRNDNVASYSIVTNNSDTITDAGKSKKRGRKRKIDSTMAVVHKINAASGVSRSTKRCRNGISWKCSKKTSHATKSKSDSIYVAMVKAIKGDMIKRISSGNVAKRDKGKTIIKYKDNDWRITCDDDHNASNTTIKVLASN

>Cl73266.1

MRAKNNAGCHGNDKIKIAKNRDMCYIWSYSIGKYKKTVNGGIYGCSYRKSRNWVDAKCHDIVVRVKRGWTSDVTKGYSRNHDCAIRSNHGYVVDSTMSCVGVSITSSKYDCAYVIHRAKAANTSADTSYVDYNNTHDIKTDICVAYKATWTISSKSSVATGRNIMTCSSNSNCIGKVCMSTTAYVHDRWRACKHHKSHGVVGRASSHGCCADVTKDDDYSVHNARMNGASCAIYHGVAHDSYVVDMKVADNVKVKHKSSVGDVSTTDDVSSMNSKDSAIVGKTNATHCINAKDSTSNNDKSWNNGTNTYVNGHKDSVTAINGKIWASKRGVNRSTKRVCRKNITSWHRHKKHAHSIYVVVVRMKRWSCSSNRHGSAKWWICGKHNADSKKASIAHGSSIISKSIIKDKIHSINDASITANTNVRDMVSKKNMAKVSADIRMIVKATYNDMIKVSSGSKNVARRKRNSRHYRDEDDDLILIACDTDLRTLIPFSGNSVSKNTVKLIVQMAND

>Cl29715.1

MGCRGWGNKYGYIDISSGDSCVGIIITSSNYVDYAVVSKAKKNKSNVDRSIADRRDDGIKVTVVCDSDATWSVSGYASSVASSGKISCSSNRSCIGKVCMSTYGYVRDSKWDHACSRHDSGVVGRSSSRGTWCSDVTKDDVSVITRMSGTSCAIYMKSDVYVITCSADKSNKMITMKHIKSACMDIMSTVIGGNWDITTKGVHDSDMDNMNSNSVDVGTSVYKIKNDTNTRKRRKRKRSSSITKDIKKHGKMDAAAINVSRSTKRICRNRGIRWYKSHTSNGATVSRVSSGITRDKGKHVISSRTNDKSIVANRVHVVIKVTYGNNTIRCIDGVKIATRKRSRKYRDADGDMIICDNDISDAILSTSEPPDSKAVIKLFVQPVGHQRLDA

>Cl88516.1

MICSNNTKGDNMNGRGSNRNVGKSKYNCNSSAGARGYTSRVYGNRVDVSSSKADNIIDIIKKITHASNRRDHVVWSVTVRKRCTTDGGAVDAYYRSRMVVDGHRGGRVYRKWSDVHNTGSSNHGYINVDSGCCVGVIIITSSTYVDYGVHVSRAKNKSDVNSYIINTRKSIAKSICDINIATWASGSSVVASSGNSCSSKRNCIGKTCMSTYDYVRDSMWHACRHDKSGVVGKSMSSGGCCDITKDDVYGSHAYVNGITSCAIYMIGISGVYVIVAHHANADSVTVKIKNVSVKGISHVIGGDNWNSSITINVGNVNINVNDVSKDSAAYVHMDDNIKIDNAAAGTSSVNVGIDADINKRKNDDKSNKKRKRKRARSISVISKHYGKMAAANHVSRSTKRICRSGITRWYKNKSDSAKNTDVVIHASDRAATVGTSIGATNIYVVGTHGNSTVSHKTNDGSAHKTTGDSTKITSAANTVKDTKSTHKYANTSAANTVKTTIKATYNTVKSSDGAKVATRSGSRKYADADGDMIIACDSDMGSVGDSRQPDHQTLIRLLVHDQNPDTN

>Cl37107.1

MVSHSSMDSKTVGINANIDSINAYRSDSNDDKNWIKMYANDVTNYKYDVVTKRRKHKMDSVTRAINGKIRASKAGVSRSTKRVCRKNISWHRRKKCVRSIYVAVVRVKRWSCSSRRHGSAKWWICGKHNADSRSKSIAYGSSISKAVKGKIDYSTKSDDITANNAASDASKKNVVKSAYIRMTVKATNDMIKHSSGKKVARRKKDSRHKYRDEDDDLILIACDTDLSNLIMSFSATTDGNNTIKLMVQMADA

>Cl84042.1

MNSVKINASCMDVMSAVIGGDISWDDTITTKVHDSDMNVISNSAVGTSSIAYHRINDTNGTVRKKRKRKRSGSSITVIKKHDKMDAAAINVSRSTKRICRRGIARWYKIGTDKSDMKSDTDIVVHTSDGSAVGVSGTTTVTDATTKDKHASTRTNTIVASRVNVVIKATYKNVVKYIDGVKKIATRKHGCRKYKDDGDMIICDNDISHAISSSSKIIIGYGKRKIWNYVSRSTKRICRRGIARWYKIGTDKSDMKSDTDIVVHTSDGSAVDVSGTTTVTDATTKDKHASTKIATRKHDCRKYKDDGDMIICDNDISHAISSSDSKAVINDTNGTVRKKRKRKRSSSITVIKKHDWMDAAAINVSRSTKRICRRGIARWYKIGTDKSDMKSDTDIVVHTSDGSAVGVSGTTTVTDATTKDKHASTTGKAVVASSTRKHSKTTTRTIVKGNMRAIISSKGKVRVVKHIVELNKNSFL

>Cl43309.1

MDSVTREGIQQNFGKPIREASKALGVSRSTLKRVFRKLNILSWPLPHRRKKCVCLSIYRWSCSSRRHGFSAKWWICGKHNAFDSRSKLSTEAYGSSLISKPLAVPPKGEKILEPLYLSQTPKESDDFIQTANPNAPQASFDMFPASPKKNVAKVSANIRMLTVKATFENDMIKFHFPLSSGLLELKKQVARRFKLKDSRLHLKYRDEDDDLILIACDTDLSNLIMSFSATTDGNNTIKLIVQMADA

>Cl63434.1

MDSVTREAIQQNFGKPIGEASKALGVSRSTLKRVCRKLNIPSWPLPHRRKKCARLSICFVVWLLVRVKRWSCSSRRHGFSAKWWICGKHNAFDSQSKLSIEAYGSSMISKPLAVSPKGEKILEPLYLSQTPKKSDDFIQTANPKAPQASFDMFPASPKKNVARVSVNIRMLTVKATFENDMIKFHFPLSSGLLELKNQVALRFKLKDSRIHLKYRDEDDDLILIACDTDLRNLLMPFSATTAGNNTIKLIVEMADA

>Cl76263.1

MKSIYDVDKDASSGSNVNANSKYCCSTSASRDNDNGSRRYTVGSRVDDSSKRHSTKSCKNMHIKTYTTRRIARNTSVSMVVMSIVIIHKTWMWGNVKDCSKDNSIDRVKDKIRHASASRSHVVWSANDKRCTTDGGVVDGYYRSRRMVVNGDHKGGRVYRKWSDVTTIDDVSYNIYGYIDVDNGSCVGVIVSSSSYVDYAVVSRAKHNKSMADRYVGDRRHDIYSMKTVCDKRAMWASGYSSIVVNSGNSSCSSNKNCIGKVCMSTSDYVRDSMWGRACRIHDKSGIVGKSTRGCCKDVTKGKYMASYARGYITSCAIYKSISDIYVIVHDNADSGTIKIKNVSSVKGISSITTKKSISSITTKSVNRNDVSKDKSVAYVHMKDDNIMDNAAAGTSIIVCNAGIDVDINKGKTTKIHHDKNTKRKKKKTMSISVKISKHYGKMAAGNGVSRSTKRICRSDIRWYKNDKSNYVKSNTDAVIRASGTIVGTSTSGTTNIYVTTHGKKSSIVVSHDKIVGSAKTTIRHIVNTATNTMIKATYKDIVKMSDGVKVATRSGSSKYDADGDMITCDDIVADSRDNAVIRVSHTAH

>Cl03881.1

MDHGVVVNVANSDDITNTVVNVANSDDITNTRAGKNVTDAGKNGNHRRHRKRKIRSVTAVRNYGKVAAKNGGKSTKRICRHGISWKKVSNKTAYVHSMCATDGSIGTGTMKTKNNDVSDVSNIANKDGARGVKRVTDKKSDSNVRVTYDSHSIYVARIVRKRRNMIKRGSVVCVSTGRDATDSRNSMSANDIDSHNMSSWSDWGDDDATNRRNSMSANDMDSLLEAQIMSESEWESDWVLDDEGNFM

>Cl48296.1

MAYNKYTITQYFHITIRQAAKELNVGLSILKYNCRDVGIKRWPYRKLKSLKTLINDFQAENGGAQPENDKREIIKMLKEEKR

>Cl53412.1

MAYNKDTICHYFHMTIRKAAKELNVGLSILKYNCRDLKSLKTLINDFQNGGAQPENDKGEIIKMLKEEKRQLEDNPDLQLARTTKMLRACNSKSKYQKRKNMSLVLSLSNQTATNAIPLLCLSPTNNSAHEEMDDVYGSDSDKHD
