# Supplementary material for: Genome-wide identification and characterization analysis of RWP-RK family genes reveal their role in flowering time of Chrysanthemum lavandulifolium
Source: BMC Plant Biol. 2023 Apr 15;23:197. doi: 10.1186/s12870-023-04201-2 (PMC10105424; doi:10.1186/s12870-023-04201-2)
Supplement: Supplementary file 2 — Supplementary Material 2 [file 12870_2023_4201_MOESM2_ESM.docx]

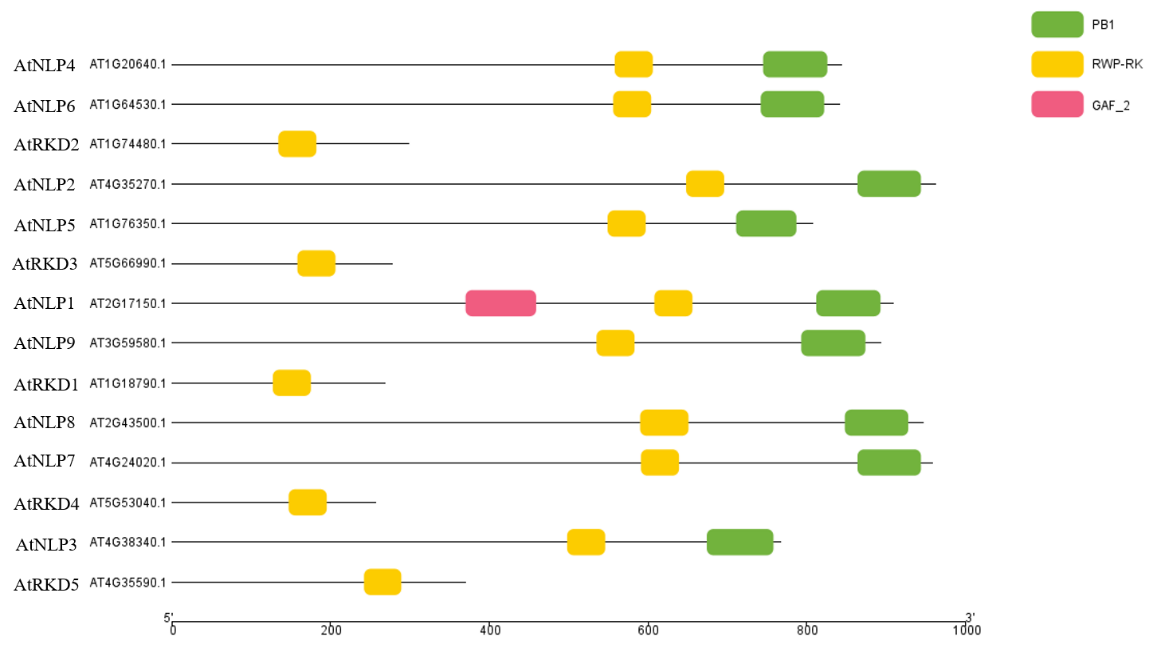


**Figure S1.** Conserved domains of AtRWP-RK


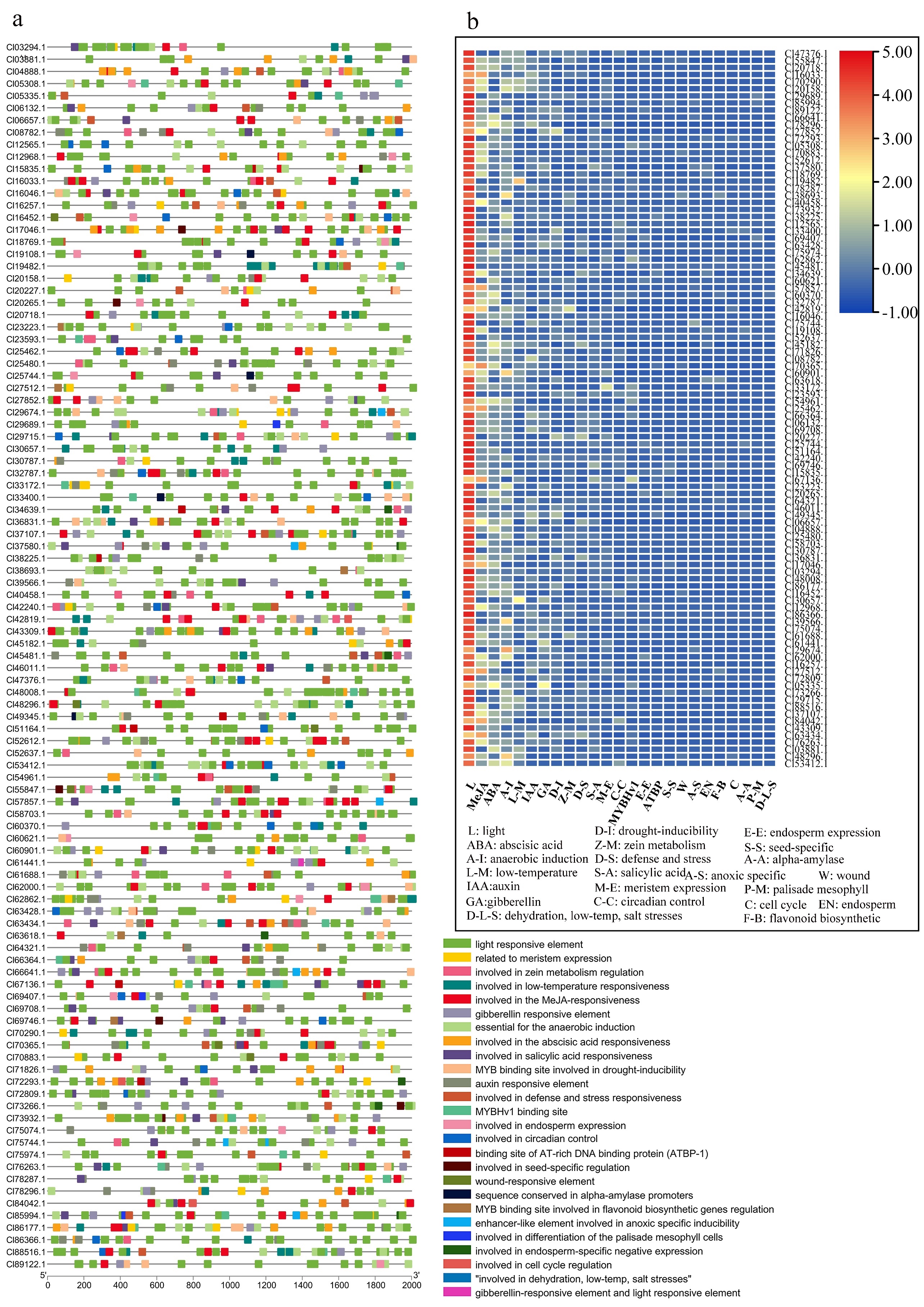


**Figure S2.** Cis-acting element on the promoter of *Chrysanthemum lavandulifolium ClRWP-RK*. **a** Cis-acting element chart based on TBtools software. **b** Heat map display of the number of cis-acting elements on the ClRWP-RK promoter.


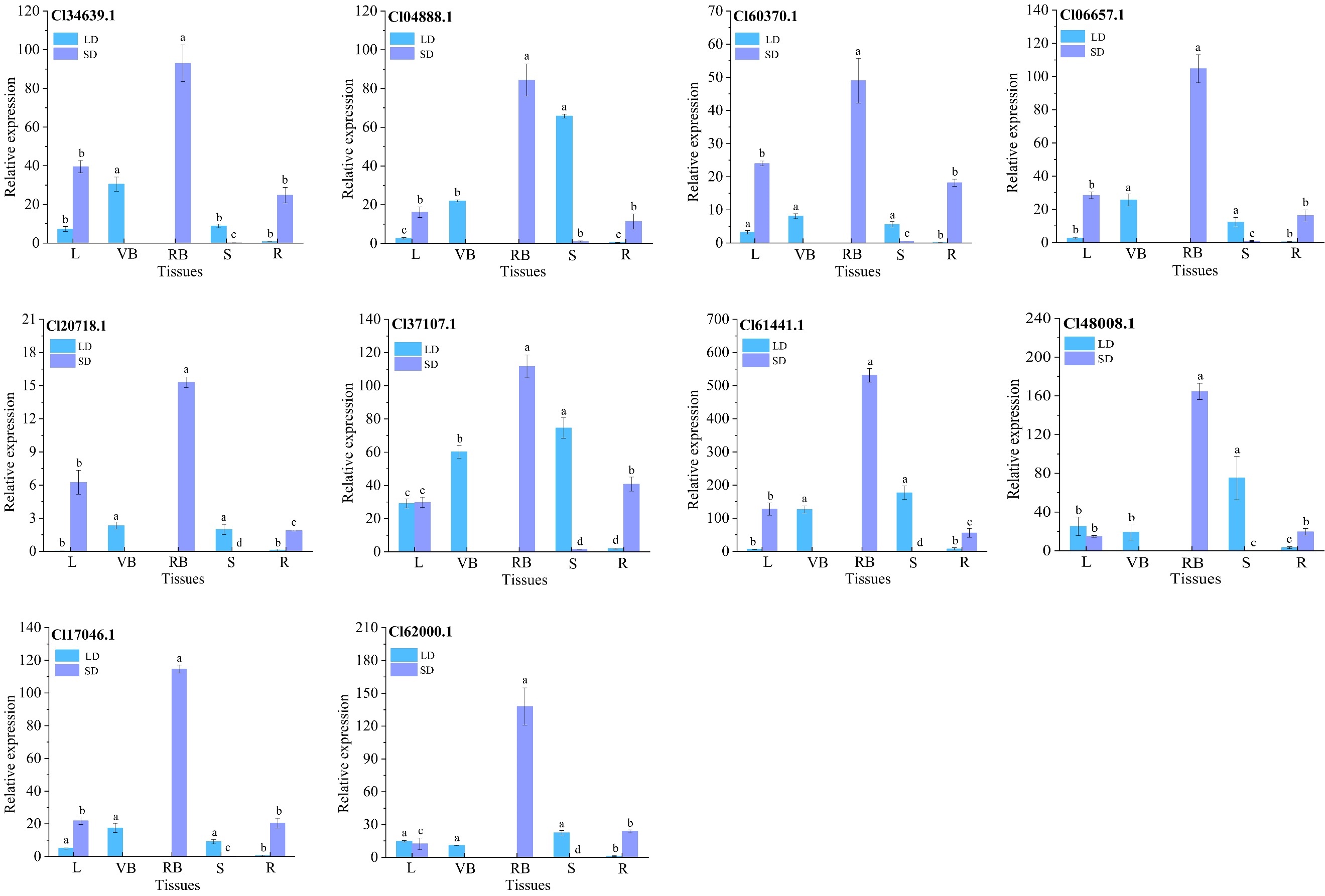


**Figure S3.** Tissue-specific expression pattern of *ClNLPs* genes in L (leaf), VB (apical meristem at long-day stage), VB (apical meristem at short-day stage), S (stem), R (root); the blue part represents long-day stage, the purple part represents short-day stage. Data shown as mean ± s.d. of three independent biological replicates, *p < 0.05.*


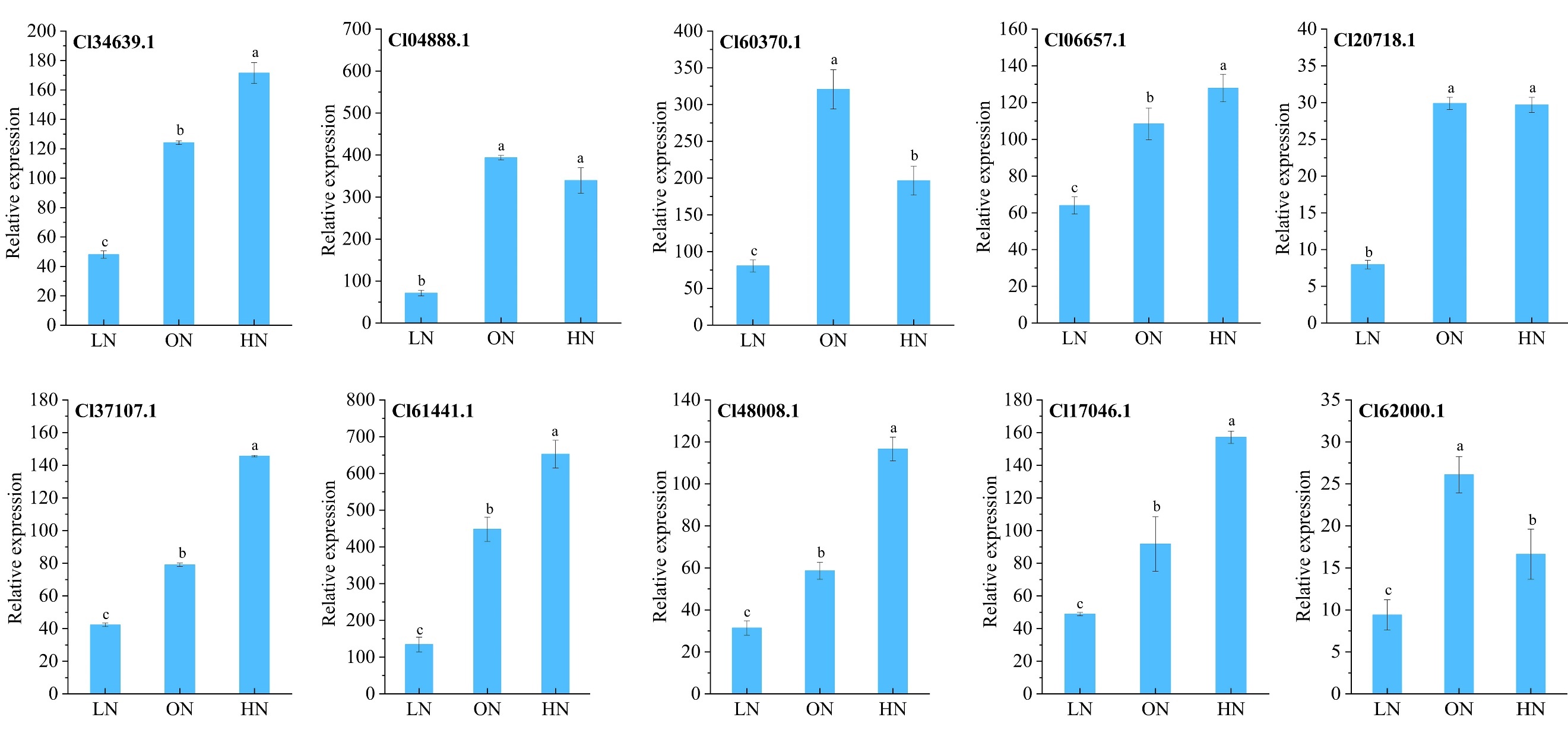


**Figure S4.** Expression verification of *ClNLPs* genes under different nitrate concentrations. Low nitrate, LN, 0.1 mM KNO₃; optimal nitrate, ON, 1.5 mM KNO₃; high nitrate, HN, 3.0 mM KNO₃. Data shown as mean ± s.d. of three independent biological replicates, *p < 0.05.*


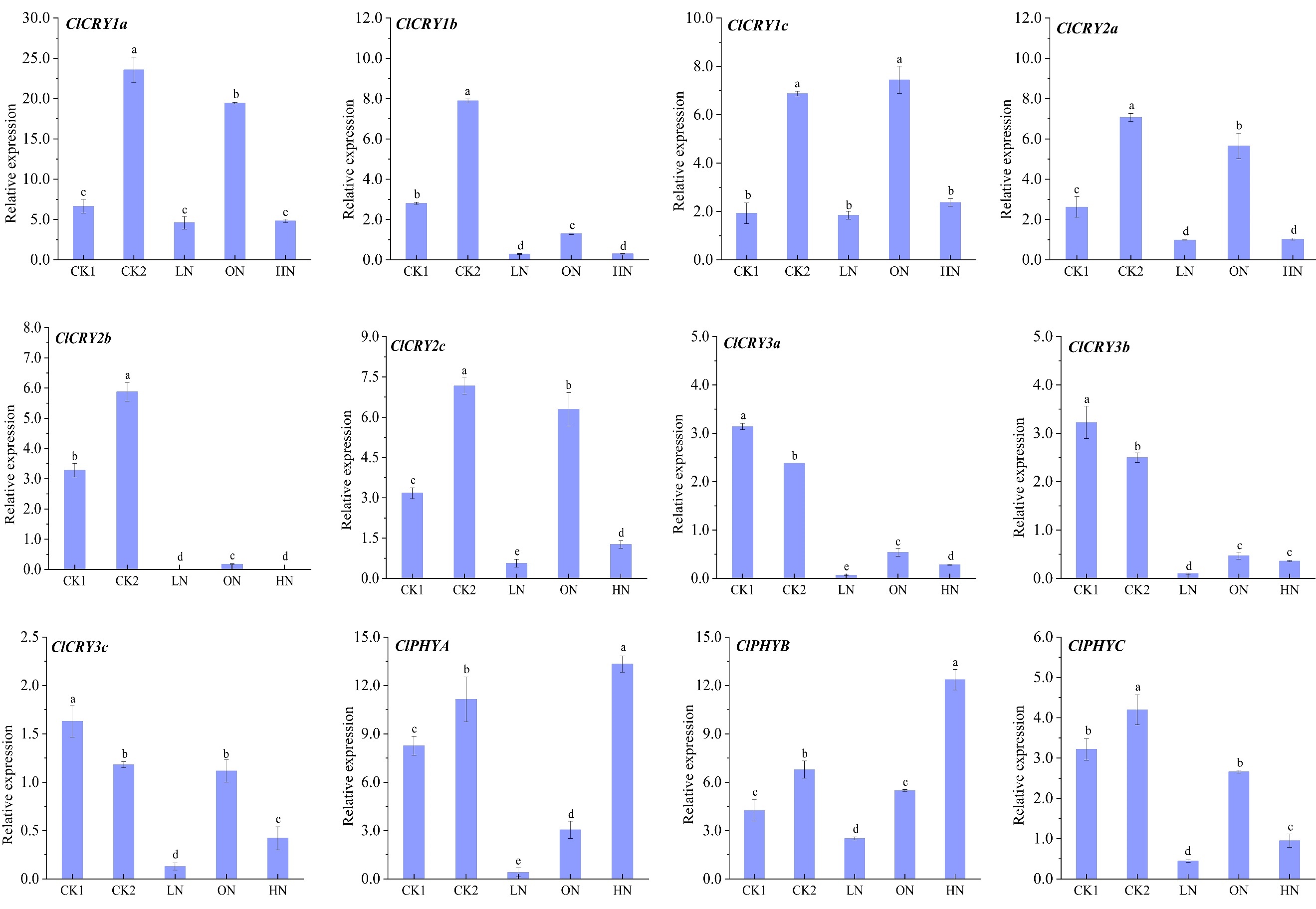


**Figure S5.** Expression verification of photoreceptor genes under different nitrate concentrations. CK1 and CK2 represent *C. lavandulifolium* leaves at long-day and short-day stages, respectively, under normal water and fertilizer management without metering; low nitrate, LN, 0.1 mM KNO₃; optimal nitrate, ON, 1.5 mM KNO₃; high nitrate, HN, 3.0 mM KNO₃. Data shown as mean ± s.d. of three independent biological replicates, *p < 0.05.*


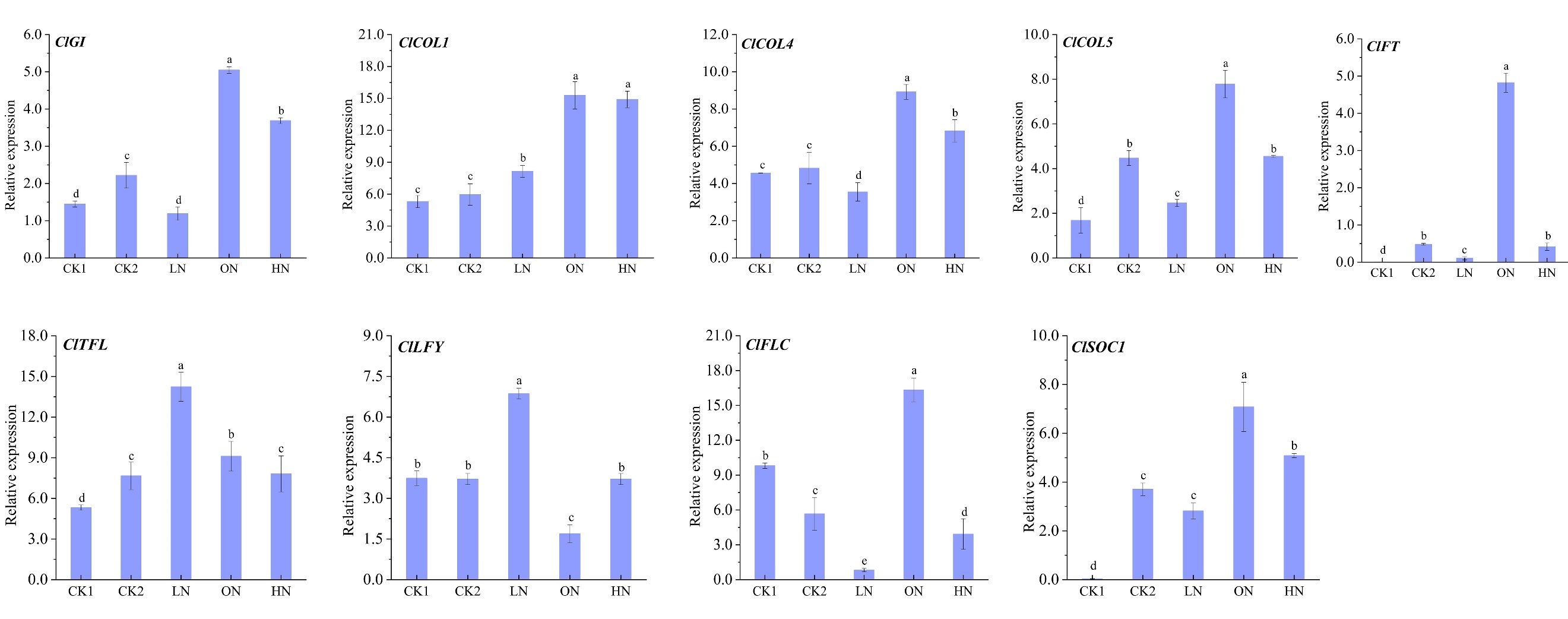


**Figure S6.** Expression verification of floral-related integrated genes under different nitrate concentrations. CK1 and CK2 represent leaves under long-day and short-day conditions, respectively, under normal water and fertilizer management without metering; low nitrate, LN, 0.1 mM KNO₃; optimal nitrate, ON, 1.5 mM KNO₃; high nitrate, HN, 3.0 mM KNO₃. Data shown as mean ± s.d. of three independent biological replicates, *p < 0.05.*

**Figure S7.** Budding time and flowering time of *C. lavandulifolium* under different nitrate concentrations. The blue part and purple part represent budding time and flowering time respectively; low nitrate, LN, 0.1 mM KNO₃; optimal nitrate, ON, 1.5 mM KNO₃; high nitrate, HN, 3.0 mM KNO₃. *p < 0.01.*

**Table S1** The distribution of *RWP-RK* genes in Compositae and other representative species

| **Family** | **Species name** | **Abbr.** | **Total number** | **Subclade** | | |
| --- | --- | --- | --- | --- | --- | --- |
|  |  |  |  | **RKDs** | **NLPs** | **Specific** |
| Composite  Cruciferae  Lauraceae  Gramineae  Solanaceae  Gramineae | *Chrysanthemum lavandulifolium*  *Chrysanthemum nankingense*  *Helianthus annuus*  *Mikania micrantha*  *Lactuca sativa*  *Arabidopsis thaliana*  *Amborella trichopoda*  *Oryza sativa*  *Solanum lycopersicum*  *Triticum aestivum* | Cl  Cn  Han  Mm  Ls  At  Atr  Os  Sl  Ta | 101  56  52  41  27  14  6  22  10  26 | 31  17  10  8  14  5  3  7  3  8 | 38  22  34  29  13  9  3  15  7  18 | 32  17  8  4  0  0  0  0  0  0 |

The predicted results of the physical and chemical properties of RWP-RKs protein were shown in supplementary table 2 (Table S2). RWP-RK protein encodes 77 to 1169 amino acids and molecular weights ranging from 0.76 to 133.22 kD, with large differences between proteins. The theoretical pI ranged from 0.35 to 10.46, all proteins were hydrophobic proteins, 83.17% of which had an index greater than 40% were unstable proteins, and the remaining 16.83% were stable proteins. The most abundant amino acid components of all ClNLPs were leucine or serine, which were relatively consistent in the same subfamily, while the most abundant amino acid components of ClRKDs were relatively inconsistent. The results of subcellular localization prediction showed that 73 members of 101 RWP-RK proteins were localized in the nucleus, which belonged to the typical characteristics of TFs.

**Table S2** Physical and chemical properties of ClRWP-PK protein

| **Subfamily** | **ID** | **Number of amino acids** | **Molecular weight (Kda)** | **hydrophobicity** | **Theoreti-cal pI** | **Formula** | **Instability index** | **Protein stability** | **The most abundant amino acid composition** | **Aliphatic index** | **Subcellular Localization Prediction** |
| --- | --- | --- | --- | --- | --- | --- | --- | --- | --- | --- | --- |
| RKDs | CL19482.1 | 342 | 39.38 | hydrophilic | 4.98 | C_1734_H_2697_N_469_O_547_S_16_ | 37.1 | stable | Asp (7.9%), Glu (7.9%) | 79.77 | Cytoplasmic, Nuclear |
|  | CL78287.1 | 672 | 75.97 | hydrophilic | 5.52 | C_3331_H_5289_N_929_O_1033_S_34_ | 44.02 | unstable | Ser (8.8%) | 83.78 | Nuclear |
|  | CL67136.1 | 271 | 30.96 | hydrophilic | 9.56 | C_1386_H_2260_N_386_O_396_S_9_ | 38.26 | stable | Leu (11.8%), Lys (11.8%) | 92.51 | Cytosol |
|  | CL51164.1 | 230 | 27.01 | hydrophilic | 6.61 | C_1192_H_1881_N_329_O_359_S_14_ | 52.92 | unstable | Leu (12.2%) | 75.48 | Nuclear |
|  | CL15835.1 | 373 | 42.31 | hydrophilic | 5.15 | C_1818_H_2855_N_515_O_595_S_27_ | 46.41 | unstable | Ser (11.0%) | 67.72 | Nuclear |
|  | CL23593.1 | 83 | 9.74 | hydrophilic | 5.5 | C_429_H_658_N_118_O_134_S_4_ | 23.5 | stable | Glu (8.4%) | 70.48 | Chloroplast |
|  | CL47376.1 | 158 | 18.58 | hydrophilic | 9.01 | C_822_H_1293_N_227_O_246_S_9_ | 44.12 | unstable | Arg (8.2%), Lys (8.2%), Ser (8.2%) | 66.65 | Chloroplast, Nuclear |
|  | CL42240.1 | 92 | 10.89 | hydrophilic | 6.96 | C_487_H_755_N_135_O_141_S_4_ | 53.2 | unstable | Ile (8.7%) | 84.78 | Cytosol |
|  | CL19108.1 | 110 | 12.85 | hydrophilic | 9.56 | C_568_H_926_N_164_O_169_S_3_ | 49.68 | unstable | Leu (11.8%) | 87.73 | cell wall |
|  | CL25744.1 | 189 | 22.27 | hydrophilic | 9.49 | C_978_H_1579_N_289_O_297_S_4_ | 47.12 | unstable | Arg (10.1%) | 73.76 | Cytosol |
|  | CL33400.1 | 176 | 20.47 | hydrophilic | 9.54 | C_895_H_1458_N_266_O_275_S_4_ | 66.89 | unstable | Leu (10.2%) | 81.99 | Cytosol |
|  | CL63428.1 | 153 | 17.97 | hydrophilic | 8.39 | C_808_H_1268_N_220_O_232_S_6_ | 55.32 | unstable | Glu (10.5%) | 84.71 | Chloroplast |
|  | CL69407.1 | 77 | 9.02 | hydrophilic | 9.18 | C_415_H_622_N_110_O_109_S_4_ | 35.95 | stable | Ser (11.7%) | 80.91 | Chloroplast |
|  | CL25462.1 | 148 | 17.36 | hydrophilic | 9.64 | C_778_H_1239_N_219_O_221_S_5_ | 45.53 | unstable | Lys (10.8%) | 84.93 | Chloroplast |
|  | CL38225.1 | 155 | 18.35 | hydrophilic | 9.49 | C_822_H_1287_N_233_O_235_S_5_ | 50.54 | unstable | Lys (9.7%) | 76.06 | Chloroplast |
|  | CL32787.1 | 126 | 14.83 | hydrophilic | 9.39 | C_674_H_1032_N_180_O_186_S_6_ | 35.99 | stable | Ser (10.3%) | 73.41 | Nuclear |
|  | CL42819.1 | 421 | 48.30 | hydrophilic | 9.25 | C_2158_H_3397_N_595_O_624_S_20_ | 44.83 | unstable | Leu (9.7%), Se r 9.7%) | 84.32 | Nuclear |
|  | CL16046.1 | 156 | 18.17 | hydrophilic | 9.59 | C_787_H_1284_N_232_O_243_S_9_ | 47.11 | unstable | Lys (12.8%) | 64.49 | Nuclear, mitochondrion |
|  | CL60621.1 | 126 | 15.10 | hydrophilic | 10.05 | C_678_H_1114_N_186_O_180_S_11_ | 16.94 | stable | Lys (14.3%) | 87.46 | Nuclear |
|  | CL48296.1 | 82 | 9.73 | hydrophilic | 9.79 | 436H_706_N_124_O_122_S_3_ | 39.05 | stable | Lys (13.4%) | 84.51 | Chloroplast |
|  | CL53412.1 | 145 | 16.51 | hydrophilic | 8.62 | C_708_H_1158_N_206_O_227_S_10_ | 40.63 | unstable | Leu (11.7%) | 76.76 | Nuclear |
|  | CL62862.1 | 209 | 24.30 | hydrophilic | 9.25 | C_1080_H_1760_N_296_O_318_S_10_ | 42 | unstable | Lys (12.4%) | 89.62 | Nuclear |
|  | CL63618.1 | 113 | 13.43 | hydrophilic | 10.04 | C_609_H_987_N_169_O_166_S_3_ | 42.97 | stable | Lys (15.0%) | 78.67 | Chloroplast |
|  | CL06132.1 | 156 | 18.03 | hydrophilic | 9.02 | C_794_H_1289_N_229_O_241_S_4_ | 45.61 | unstable | Lys (10.3%) | 91.35 | Cytosol, Nuclear |
|  | CL66364.1 | 136 | 15.73 | hydrophilic | 9.63 | C_699_H_1152_N_202_O_201_S_4_ | 38.02 | stable | Leu (11.0%), Lys (11.0%) | 102.57 | Cytosol |
|  | CL69708.1 | 150 | 17.57 | hydrophilic | 10.22 | C_780_H_1306_N_232_O_219_S_4_ | 43.14 | unstable | Leu (12.7%) | 106 | Cytosol |
|  | CL20227.1 | 115 | 13.45 | hydrophilic | 9.49 | C_604_H_975_N_169_O_172_S_3_ | 52.97 | unstable | Lys (13.0%) | 86.61 | Cytosol |
|  | CL29689.1 | 158 | 18.37 | hydrophilic | 9.2 | C_811_H_1319_N_233_O_242_S_5_ | 47.21 | unstable | Lys (11.4%) | 88.29 | Cytosol |
|  | CL52637.1 | 93 | 10.96 | hydrophilic | 6.58 | C_492_H_795_N_133_O_143_S_3_ | 45.86 | unstable | Ile (11.8%), Lys (11.8%) | 97.63 | Nuclear |
| NLPs | CL55847.1 | 1008 | 114.33 | hydrophilic | 5.85 | C_5065_H_7956_N_1362_O_1552_S_49_ | 45.47 | unstable | Ser (8.9%) | 81.42 | Nuclear |
|  | CL20718.1 | 609 | 68.31 | hydrophilic | 6.3 | C_3016_H_4722_N_828_O_933_S_25_ | 45.68 | unstable | Ser (11.7%) | 77.45 | Nuclear |
|  | CL16033.1 | 698 | 78.68 | hydrophilic | 7.27 | C_3459_H_5454_N_964_O_1071_S_31_ | 44.44 | unstable | Ser (12.0%) | 77.21 | Nuclear |
|  | CL70290.1 | 722 | 81.85 | hydrophilic | 5.93 | C_3601_H_5650_N_992_O_1112_S_38_ | 44.35 | unstable | Ser (10.0%) | 78.01 | Nuclear |
|  | CL20158.1 | 783 | 89.05 | hydrophilic | 5.36 | C_3924_H_6139_N_1077_O_1216_S_37_ | 43.61 | unstable | Ser (9.1%) | 80.14 | Nuclear |
|  | CL85994.1 | 654 | 74.26 | hydrophilic | 5.48 | C_3274_H_5130_N_896_O_1016_S_30_ | 41.21 | unstable | Leu (8.4%) | 80.61 | Nuclear |
|  | CL89122.1 | 1169 | 133.22 | hydrophilic | 5.64 | C_5943_H_9227_N_1585_O_1786_S_55_ | 41.7 | unstable | Leu (8.8%) | 83.11 | Nuclear |
|  | CL66641.1 | 717 | 82.78 | hydrophilic | 6.45 | C_3695_H_5724_N_1002_O_1096_S_33_ | 54.16 | unstable | Leu (8.8%) | 78.05 | Nuclear |
|  | CL78296.1 | 688 | 78.73 | hydrophilic | 6.26 | C_3509_H_5426_N_934_O_1055_S_36_ | 41.24 | unstable | Ser (9.7%) | 77.17 | Nuclear |
|  | CL27852.1 | 1021 | 116.66 | hydrophilic | 5.84 | C_5221_H_8102_N_1394_O_1553_S_44_ | 43.94 | unstable | Leu (9.1%) | 85.14 | Cytosol |
|  | CL72293.1 | 654 | 74.19 | hydrophilic | 5.44 | C_3264_H_5118_N_892_O_1021_S_31_ | 39.12 | stable | Leu (8.7%) | 79.11 | Nuclear |
|  | CL05308.1 | 644 | 73.20 | hydrophilic | 5.57 | C_3218_H_5028_N_878_O_1002_S_36_ | 39.31 | stable | Ser (8.4%) | 75.82 | Cytosol |
|  | CL70883.1 | 688 | 79.21 | hydrophilic | 6.65 | C_3547_H_5516_N_942_O_1046_S_35_ | 45.33 | unstable | Leu (9.4%) | 80.6 | Nuclear |
|  | CL52612.1 | 602 | 69.23 | hydrophilic | 6.37 | C_3083_H_4774_N_836_O_938_S_21_ | 40.95 | unstable | Ser (9.5%) | 78.21 | Nuclear |
|  | CL37580.1 | 654 | 74.10 | hydrophilic | 5.78 | C_3253_H_5131_N_901_O_1015_S_31_ | 41.12 | unstable | Leu (8.4%), Ser (8.4%) | 80.31 | Nuclear |
|  | CL18769.1 | 743 | 84.90 | hydrophilic | 5.49 | C_3774_H_5865_N_1005_O_1156_S_34_ | 47.95 | unstable | Leu (8.6%), Ser (8.6%) | 8.2 | Nuclear |
|  | CL38693.1 | 754 | 84.82 | hydrophilic | 6.69 | C_3748_H_5916_N_1030_O_1149_S_32_ | 49.6 | unstable | Ser (11.3%) | 81.68 | Nuclear |
|  | CL40458.1 | 456 | 51.23 | hydrophilic | 5.32 | C_2286_H_3550_N_608_O_686_S_22_ | 44.73 | unstable | Ser (8.3%) | 85.7 | Cytosol |
|  | CL73932.1 | 612 | 68.55 | hydrophilic | 6.29 | C_3015_H_4828_N_826_O_938_S_28_ | 58.77 | unstable | Ser (10.1%) | 82.92 | Nuclear |
|  | CL12565.1 | 1100 | 125.73 | hydrophilic | 5.93 | C_5603_H_8691_N_1497_O_1686_S_54_ | 45.04 | unstable | Leu (9.0%) | 80.38 | Nuclear |
|  | CL75974.1 | 770 | 86.26 | hydrophilic | 6 | C_3814_H_5992_N_1030_O_1177_S_36_ | 46.58 | unstable | Ser (11.2%) | 78.99 | Nuclear |
|  | CL45481.1 | 733 | 80.36 | hydrophilic | 5.32 | C_3489_H_5534_N_982_O_1127_S_34_ | 54.53 | unstable | Ser (10.6%) | 75.78 | Nuclear |
|  | CL34639.1 | 822 | 90.66 | hydrophilic | 5.85 | C_3956_H_6247_N_1117_O_1245_S_40_ | 53.08 | unstable | Ser (10.3%) | 77.42 | Nuclear |
|  | CL57857.1 | 666 | 76.02 | hydrophilic | 7.26 | C_3408_H_5365_N_915_O_994_S_30_ | 46.36 | unstable | Leu (11.3%) | 89.28 | Cytosol |
|  | CL60370.1 | 730 | 83.33 | hydrophilic | 7.25 | C_3724_H_5872_N_1008_O_1100_S_30_ | 49.63 | unstable | Leu (11.5%) | 90.11 | Nuclear |
|  | CL75744.1 | 941 | 102.86 | hydrophilic | 5.22 | C_4471_H_7016_N_1236_O_1448_S_50_ | 46.42 | unstable | Ser (11.9%) | 71.31 | Nuclear |
|  | CL45182.1 | 178 | 20.23 | hydrophilic | 9.48 | C_894_H_1438_N_254_O_268_S_6_ | 38.2 | stable | Leu (10.7%), Ser (10.7%) | 84.89 | Mitochondrial |
|  | CL71826.1 | 622 | 69.91 | hydrophilic | 6.58 | C_3073_H_4884_N_844_O_942_S_37_ | 50.99 | unstable | Ser (10.8%) | 84.89 | Nuclear |
|  | CL08782.1 | 686 | 77.90 | hydrophilic | 6.41 | C_3490_H_5462_N_926_O_1022_S_36_ | 42.97 | unstable | Leu (12.0%) | 90.79 | Nuclear |
|  | CL70365.1 | 818 | 92.72 | hydrophilic | 7.04 | C_4097_H_6516_N_1142_O_1249_S_30_ | 42.85 | unstable | Leu (9.9%) | 88.14 | Nuclear |
|  | CL60901.1 | 715 | 0.77 | hydrophilic | 7 | C_3573_H_5643_N_1001_O_1078_S_28_ | 45.72 | unstable | Leu (10.2%) | 86.53 | Nuclear |
|  | CL33172.1 | 827 | 92.12 | hydrophilic | 5.64 | C_4047_H_6344_N_1120_O_1275_S_32_ | 46.39 | unstable | Ser (9.9%) | 75.55 | Nuclear |
|  | CL54961.1 | 767 | 85.73 | hydrophilic | 6.1 | C_3783_H_5959_N_1023_O_1177_S_35_ | 41.94 | unstable | Ser (11.2%) | 80.34 | Nuclear |
|  | CL69746.1 | 703 | 79.88 | hydrophilic | 0.35 | C_3530_H_5554_N_982_O_1073_S_30_ | 41.89 | unstable | Ser (10.2%) | 78.45 | Nuclear |
|  | CL23223.1 | 820 | 92.74 | hydrophilic | 5.85 | C_4109_H_6447_N_1119_O_1264_S_31_ | 51.82 | unstable | Ser (9.6%) | 81.26 | Nuclear |
|  | CL20265.1 | 690 | 77.42 | hydrophilic | 5.11 | C_3424_H_5388_N_922_O_1068_S_27_ | 47.87 | unstable | Leu (10.1%) | 86.81 | Nuclear |
|  | CL64321.1 | 730 | 82.36 | hydrophilic | 5.06 | C_3604_H_5665_N_987_O_1147_S_37_ | 58.54 | unstable | Ser (10.0%) | 77.03 | Nuclear |
|  | CL46011.1 | 418 | 47.41 | hydrophilic | 5.78 | C_2119_H_3340_N_584_O_629_S_1_ | 39.13 | stable | Leu (11.7%) | 92.78 | Nuclear |
|  | CL49345.1 | 637 | 70.35 | hydrophilic | 5.25 | C_3078_H_4847_N_853_O_984_S_25_ | 48.82 | unstable | Ser (9.4%) | 83.08 | Nuclear |
|  | CL06657.1 | 693 | 78.88 | hydrophilic | 5.64 | C_3494_H_5516_N_944_O_1071_S_31_ | 52.86 | unstable | Ser (10.1%) | 83.23 | Nuclear |
|  | CL04888.1 | 273 | 31.22 | hydrophilic | 9.48 | C_1381_H_2241_N_391_O_403_S_14_ | 65.22 | unstable | Ser (10.6%) | 85.71 | Nuclear |
|  | CL25480.1 | 743 | 84.30 | hydrophilic | 5.13 | C_3710_H_5804_N_1020_O_1161_S_32_ | 57.06 | unstable | Ser (9.2%) | 80.13 | Nuclear |
|  | CL58703.1 | 464 | 52.46 | hydrophilic | 6.4 | C_2324_H_3677_N_655_O_703_S_13_ | 40.51 | unstable | Leu (10.3%) | 85.91 | Nuclear |
|  | CL30787.1 | 419 | 46.33 | hydrophilic | 5.43 | C_2054_H_3200_N_552_O_639_S_15_ | 40.82 | unstable | Ser (9.5%) | 78.16 | Cytosol |
|  | CL36831.1 | 787 | 88.66 | hydrophilic | 6.4 | C_3920_H_6184_N_1078_O_1201_S_32_ | 46.45 | unstable | Leu (11.4%) | 87.66 | Nuclear |
|  | CL17046.1 | 741 | 83.74 | hydrophilic | 5.98 | C_3688_H_5865_N_1007_O_1134_S_40_ | 54.09 | unstable | Ser (10.4%) | 84.4 | Nuclear |
|  | CL03294.1 | 420 | 47.26 | hydrophilic | 6.82 | C_2105_H_3284_N_572_O_620_S_23_ | 43.86 | unstable | Leu (8.3%) | 83.31 | Chloroplast |
|  | CL48008.1 | 757 | 85.41 | hydrophilic | 8.21 | C_3797_H_5999_N_1039_O_1138_S_31_ | 47.88 | unstable | Leu (9.6%) | 82.99 | Nuclear |
|  | CL86177.1 | 458 | 51.31 | hydrophilic | 4.97 | C_2262_H_3509_N_603_O_706_S_27_ | 39.29 | stable | Ser (8.1%) | 78.34 | Nuclear |
|  | CL16452.1 | 395 | 44.50 | hydrophilic | 5.11 | C_1957_H_3059_N_527_O_608_S_25_ | 43.4 | unstable | Ser (7.8%) | 78.25 | Chloroplast |
|  | CL30657.1 | 174 | 19.83 | hydrophilic | 6.37 | C_874_H_1408_N_244_O_263_S_9_ | 72.03 | unstable | Leu (10.3%) | 94.66 | Nuclear |
|  | CL12968.1 | 608 | 68.31 | hydrophilic | 4.98 | C_3005_H_4696_N_814_O_943_S_31_ | 59.06 | unstable | Ser (10.7%) | 80.26 | Chloroplast |
|  | CL86366.1 | 392 | 43.28 | hydrophilic | 5.18 | C_1905_H_2972_N_514_O_602_S_18_ | 48.57 | unstable | Ser (9.9%) | 75.1 | Chloroplast |
|  | CL39566.1 | 833 | 93.20 | hydrophilic | 5.73 | :C_4095_H_6485_N_1139_O_1281_S_32_ | 47.41 | unstable | Leu (9.7%) | 87.02 | Nuclear |
|  | CL75074.1 | 226 | 25.79 | hydrophilic | 8.74 | C_1135_H_1849_N_325_O_347_S_6_ | 38.54 | stable | Leu (11.1%), Ser (11.1%) | 94.47 | Nuclear |
|  | CL61688.1 | 172 | 19.12 | hydrophilic | 10.46 | C_820_H_1387_N_257_O_251_S_8_ | 66.29 | unstable | Ser (12.2%) | 84.42 | Nuclear |
|  | CL61441.1 | 764 | 86.48 | hydrophilic | 8.97 | C_3861_H_6078_N_1076_O_1130_S_26_ | 45.29 | unstable | Leu (10.1%), Ser (10.1%) | 85.21 | Nuclear |
|  | CL29674.1 | 609 | 68.82 | hydrophilic | 6.56 | C_3016_H_4780_N_834_O_944_S_31_ | 53.44 | unstable | Ser (11.5%) | 76.96 | Nuclear |
|  | CL62000.1 | 789 | 8.92 | hydrophilic | 8.62 | C_3929_H_6254_N_1098_O_1199_S_27_ | 48.64 | unstable | Leu (10.3%), Ser (10.3%) | 84.01 | Nuclear |
|  | CL16257.1 | 639 | 72.32 | hydrophilic | 8.53 | C_3223_H_5095_N_879_O_962_S_24_ | 54.3 | unstable | Ser (10.8%) | 87.23 | Nuclear |
|  | CL27512.1 | 771 | 87.31 | hydrophilic | 8.33 | C_3893_H_6130_N_1056_O_1164_S_30_ | 53.77 | unstable | Ser (10.9%) | 85.69 | Chloroplast |
|  | CL72809.1 | 815 | 91.78 | hydrophilic | 5.51 | C_4048_H_6449_N_1097_O_1267_S_32_ | 51.34 | unstable | Leu (10.8%) | 88.92 | Nuclear |
|  | CL05335.1 | 358 | 39.91 | hydrophilic | 5.59 | C_1740_H_2784_N_476_O_560_S_18_ | 34.61 | stable | Ler (11.2%) | 82.23 | Nuclear |
|  | CL73266.1 | 687 | 78.01 | hydrophilic | 8.64 | C_3477_H_5445_N_959_O_1015_S_34_ | 37.13 | stable | Leu (9.9%) | 85.27 | Nuclear |
|  | CL29715.1 | 530 | 59.58 | hydrophilic | 5.86 | C_2630_H_4181_N_715_O_808_S_26_ | 55.36 | unstable | Ser (10.8%) | 84.36 | Nuclear |
|  | CL88516.1 | 805 | 89.41 | hydrophilic | 5.17 | C_3933_H_6222_N_1078_O_1248_S_26_ | 49.75 | unstable | Leu (10.2%) | 87.17 | Nuclear |
|  | CL37107.1 | 326 | 37.26 | hydrophilic | 9.66 | C_1653_H_2657_N_469_O_482_S_14_ | 49.9 | unstable | Ser (10.4%) | 84.66 | Nuclear |
|  | CL84042.1 | 646 | 72.40 | hydrophilic | 8.2 | C_3198_H_5196_N_884_O_983_S_20_ | 47.52 | unstable | Leu (9.3%) | 88.85 | Chloroplast |
|  | CL43309.1 | 246 | 27.82 | hydrophilic | 9.79 | C_1239_H_1990_N_350_O_355_S_11_ | 48.13 | unstable | Ser (11.0%) | 82.52 | Nuclear |
|  | CL63434.1 | 256 | 28.92 | hydrophilic | 9.91 | C_1293_H_2087_N_365_O_360_S_13_ | 48.93 | unstable | Leu (10.2%), Ser (10.2%) | 87.66 | Nuclear |
|  | CL76263.1 | 844 | 95.32 | hydrophilic | 5.7 | C_4226_H_6728_N_1130_O_1300_S_36_ | 47.57 | unstable | Leu (10.9%) | 90.26 | Nuclear |
|  | CL03881.1 | 328 | 36.96 | hydrophilic | 5.12 | C_1590_H_2544_N_466_O_522_S_13_ | 47.22 | unstable | Leu (9.1%) | 7.23 | Nuclear |

**Table S3** *ClRWP-PKs* members identified in transcriptome of *C. lavandulifolium*

| **Number** | **Subfamily** | **Gene ID** | **Rename** | **FPKM values** | | |
| --- | --- | --- | --- | --- | --- | --- |
|  |  |  |  | **S1** | **S2** | **L** |
| 1 | RKD | EVM0051164.1 | Cl51164.1 | 0 | 0 | 0 |
| 2 |  | EVM0015835.1 | Cl15835.1 | 0.014530667 | 0 | 0.005258667 |
| 3 |  | EVM0023593.1 | Cl23593.1 | 0 | 0 | 0 |
| 4 |  | EVM0042240.1 | Cl42240.1 | 0 | 0 | 0 |
| 5 |  | EVM0033400.1 | Cl33400.1 | 0 | 0 | 0 |
| 6 |  | EVM0038225.1 | Cl38225.1 | 0 | 0 | 0 |
| 7 |  | EVM0042819.1 | Cl42819.1 | 0 | 0 | 0 |
| 8 |  | EVM0060621.1 | Cl60621.1 | 0 | 0 | 0 |
| 9 |  | EVM0053412.1 | Cl53412.1 | 0 | 0 | 0 |
| 10 |  | EVM0006132.1 | Cl06132.1 | 0 | 0 | 0 |
| 11 |  | EVM0029689.1 | Cl29689.1 | 0 | 0 | 0 |
| 12 | NLP | EVM0055847.1 | Cl55847.1 | 2.815594667 | 2.273683 | 4.410663667 |
| 13 |  | EVM0020718.1 | Cl20718.1 | 5.288110333 | 3.713394667 | 5.694100667 |
| 14 |  | EVM0027852.1 | Cl27852.1 | 0.008297 | 0.005457333 | 0.016074 |
| 15 |  | EVM0052612.1 | Cl52612.1 | 1.643119 | 1.272206667 | 5.135923 |
| 16 |  | EVM0037580.1 | Cl37580.1 | 0 | 0.17683 | 0 |
| 17 |  | EVM0038693.1 | Cl38693.1 | 1.584625 | 0.563066 | 7.154488667 |
| 18 |  | EVM0040458.1 | Cl40458.1 | 1.166638 | 0.624204667 | 4.035379667 |
| 19 |  | EVM0012565.1 | Cl12565.1 | 0.297830333 | 0.034044333 | 1.702011333 |
| 20 |  | EVM0034639.1 | Cl34639.1 | 12.94906033 | 12.62020833 | 28.52885833 |
| 21 |  | EVM0060370.1 | Cl60370.1 | 0.31082 | 0.148848 | 32.91578033 |
| 22 |  | EVM0045182.1 | Cl45182.1 | 0 | 0 | 0 |
| 23 |  | EVM0060901.1 | Cl60901.1 | 0.125712 | 0.081219333 | 0.161846 |
| 24 |  | EVM0033172.1 | Cl33172.1 | 4.781869333 | 6.420448 | 6.834572 |
| 25 |  | EVM0054961.1 | Cl54961.1 | 0 | 0 | 0 |
| 26 |  | EVM0023223.1 | Cl23223.1 | 2.226570333 | 1.627097667 | 4.940301333 |
| 27 |  | EVM0020265.1 | Cl20265.1 | 3.828993333 | 3.554879 | 8.141905667 |
| 28 |  | EVM0064321.1 | Cl64321.1 | 0.734364333 | 0.417418333 | 0.663009333 |
| 29 |  | EVM0046011.1 | Cl46011.1 | 0.271257667 | 0.231282667 | 0.360773667 |
| 30 |  | EVM0006657.1 | Cl06657.1 | 10.77527067 | 9.392497333 | 12.93643667 |
| 31 |  | EVM0004888.1 | Cl04888.1 | 0.209463667 | 0.025848333 | 13.97100367 |
| 32 |  | EVM0025480.1 | Cl25480.1 | 0.267588667 | 0.042735667 | 0.56465 |
| 33 |  | EVM0058703.1 | Cl58703.1 | 0.165904333 | 0 | 0.137606333 |
| 34 |  | EVM0030787.1 | Cl30787.1 | 3.173140333 | 2.953818 | 3.445486 |
| 35 |  | EVM0017046.1 | Cl17046.1 | 1.113188667 | 1.239834667 | 1.071979 |
| 36 |  | EVM0048008.1 | Cl48008.1 | 15.39288567 | 8.521635333 | 5.133613667 |
| 37 |  | EVM0039566.1 | Cl39566.1 | 0.194876 | 0.308586333 | 0.116634667 |
| 38 |  | EVM0061441.1 | Cl61441.1 | 5.132668667 | 4.760982333 | 3.370027333 |
| 39 |  | EVM0029674.1 | Cl29674.1 | 1.312840333 | 0.693963333 | 3.254265667 |
| 40 |  | EVM0062000.1 | Cl62000.1 | 22.04855767 | 32.942404 | 6.171990667 |
| 41 |  | EVM0016257.1 | Cl16257.1 | 0 | 0.030629333 | 3.066010667 |
| 42 |  | EVM0027512.1 | Cl27512.1 | 0.072093667 | 0.137003333 | 6.079059667 |
| 43 |  | EVM0005335.1 | Cl05335.1 | 3.065301667 | 1.465527333 | 2.696806667 |
| 44 |  | EVM0029715.1 | Cl29715.1 | 1.365751333 | 1.53416 | 1.644909 |
| 45 |  | EVM0037107.1 | Cl37107.1 | 4.841167333 | 1.707560667 | 2.941618 |

Note: Leaf represents leaves of *C. lavandulifolium*, S1 and S2 represent the apical meristem before and under floral transition in *C. lavandulifolium*, respectively.

**Table.S4** List of primers

| **Gene** | **L primer** | **R primer** |
| --- | --- | --- |
| Cl20718 | CCTCCTGCTCGAGTATTTCG | GCACCTTGTGAGCTTGTTGA |
| Cl34639 | ACCTTCAAAAAGGCCAAGGT | TGCTGATCCTTGCAGTCAAC |
| Cl60370 | CTTGCTATTGGCAGGCTTTC | CTTTGTGCCACTTGGGTTTT |
| Cl06657 | AGCTCGAATGAGTGGGCTAA | CACTACGTGCTGGCAAGAAA |
| Cl04888 | TATACCCAGTTGGCCAAAGC | TTGCTTGAACAACTCCATCG |
| Cl17046 | TATTGGCATGGTCCTGATGA | GTTTGTTCGTGCTGCTGTGT |
| Cl48008 | CACGCATGAGTGGGTTAATG | GGTGTTGGCAGTTGTGATTG |
| Cl61441 | GTCGGGAAGCCAATAGATGA | TGAGTCGGTAACACGAGCAG |
| Cl62000 | TGTGGCCAGTGCTATCTCAG | GTCAGCATCACAAGCAAGGA |
| Cl37107 | AAAACCATTGGCTGTTCCAC | GACGAAAGCGGAAAATGAAA |
| *ClPHYA* | ATTCCCCCAATCTTTGGAAC | CTTTTCCGCTTCTTCACTGG |
| *ClPHYB* | ATCACACCAACCGAGTCACA | TGATCTCTTTCGCAGTGTGG |
| *ClPHYC* | GAAGCTGTTGGTGTCCCATT | GCGTTTGCAACAAGGGTAAT |
| *ClGI* | GCACAGCTTCATGCTGGTAA | GAAGACTCGATTGCCTTTGC |
| *ClCOL1* | CGATGCAAACGTTCATTCAG | ATCGAAACACCTTCGTCACC |
| *ClCOL4* | TTATCCGGCGACTTTCAAAC | AATCCAGTGGGTGAAGTTGG |
| *ClCOL5* | CTAAGTCCGCCAGCTAATCG | CTCATGACCCTAGCCTCTCG |
| *ClFT* | TTGCATTGGCTGGTTACTGA | GCTGGGGCATACACAGTTTT |
| *ClTFL* | ACAGGACGGGAGATTGTGAG | GGCATTTTCTCGTTGAGCAT |
| *ClLFY* | CCTGATGCACTTTCAGCAAA | CGTAAACCCAAGCTCCGATA |
| *ClFLC* | GGAAATTGGTGACGTTGCTT | GTCCCGGAGAAAACAAAACA |
| *ClSOC1* | GAAGATGCTGAGTGGCAACA | TTCCGAGCTCGAATAATGCT |

Note: The sequences come from the *C. lavandulifolium* transcriptome database.
